# Look What You Made Me Do: Discerning Feature for Classification of Endocrine-Disrupting Chemical Binding to Steroid Hormone Receptors

**DOI:** 10.1021/acs.jcim.4c02288

**Published:** 2025-04-09

**Authors:** Azam Rashidian, Sini Pitkänen, Vinicius Goncalves Maltarollo, Ulrich Schoppmeier, Ekaterina Shevchenko, Prasanthi Medarametla, Antti Poso, Jenni Küblbeck, Paavo Honkakoski, Thales Kronenberger

**Affiliations:** 1 Department of Pharmaceutical and Medicinal Chemistry, Institute of Pharmaceutical Sciences, Eberhard-Karls-Universität Tübingen, Auf der Morgenstelle 8, 72076 Tübingen, Germany; 2 Tübingen Center for Academic Drug Discovery & Development (TüCAD2), 72076 Tübingen, Germany; 3 Interfaculty Institute of Microbiology and Infection Medicine (IMIT), University of Tübingen, Tübingen, Germany; Partner-site Tübingen, German Center for Infection Research (DZIF), 72076 Tübingen, Germany; 4 A.I. Virtanen Institute for Molecular Sciences, University of Eastern Finland, P.O. Box 1627, 70210 Kuopio, Finland; 5 Departamento de Produtos Farmacêuticos, Faculdade de Farmácia, Universidade Federal de Minas Gerais, Av. Presidente Antônio Carlos, 6627, Pampulha, 31270-901 Belo Horizonte, MG, Brazil; 6 School of Pharmacy, Faculty of Health Sciences, University of Eastern Finland, 70211 Kuopio, Finland

## Abstract

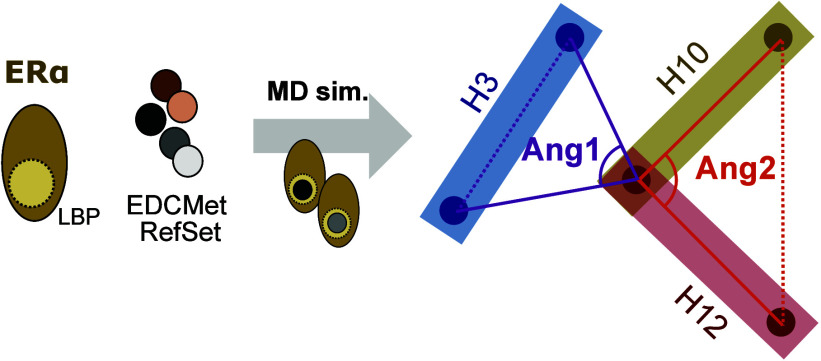

Exposure to metabolism-disrupting chemicals, which are a specific type of endocrine-disrupting chemical (EDC), is linked to metabolic problems such as dyslipidemia, insulin resistance, and hepatic steatosis. Steroid hormone receptors (SHRs) within the nuclear receptor superfamily are well-known targets for EDCs in reproductive tissues and, to a lesser extent, in liver. In this study, we investigated how five well-established SHR ligands and eight EDCs including pesticides, plasticizers, pharmaceuticals, flame retardants, industrial chemicals, and their metabolites affect estrogen (ERα in reproductive tissues) and glucocorticoid (GR in liver) receptors. We investigated the utility of structural molecular modeling to classify EDC binding to ERα and GR. To this end, we modeled a set of EDC binding to ER and GR using unbiased all-atom long-time scale molecular dynamics (MD) simulations and compared them against known established SHR agonists and antagonists. We systematically evaluated MD-derived variables such as protein–ligand interactions and binding energy, folding secondary structure elements, distances, and angles as relevant parameters. Our findings suggest that the well-established H12 folding and conformational angles can be discerning features for binding of EDCs to SHRs. Although SHR activation often involves changes in H12 folding and geometry, GR displayed less flexibility in this region, suggesting that protein–ligand interaction and binding energy are more relevant for its classification. We show that MD simulations combined with experimental assays can be a useful tool for studying novel EDCs by providing relevant structural features for their classification.

## 1 Introduction

Endocrine-disrupting chemicals (EDCs) are structurally diverse, often lipophilic substances capable of bioaccumulation. Examples of such persistent organic pollutants include fungicides, pesticides, plasticizers, and various polyhalogenated organic compounds, which are present in consumer products, the environment, and industrial emissions.^1,2^ EDCs can mimic endocrine hormones and disrupt the functions of the endocrine system by binding to nuclear receptors (NRs), which leads to adverse hormonal or metabolic health effects at the organism level.^1−4^ Because EDCs tend to accumulate in the liver and adipose tissue, insulin resistance and alteration of lipid, cholesterol, and bile acid metabolism are common consequences of EDC exposure^1^ and can contribute to type 2 diabetes and metabolic dysfunction-associated steatosis liver disease.^5,6^ Therefore, some EDCs have been coined as metabolism-disrupting chemicals. The European Commission has funded several European Cluster projects to improve the EDC identification.^2^

**Figure 1:**
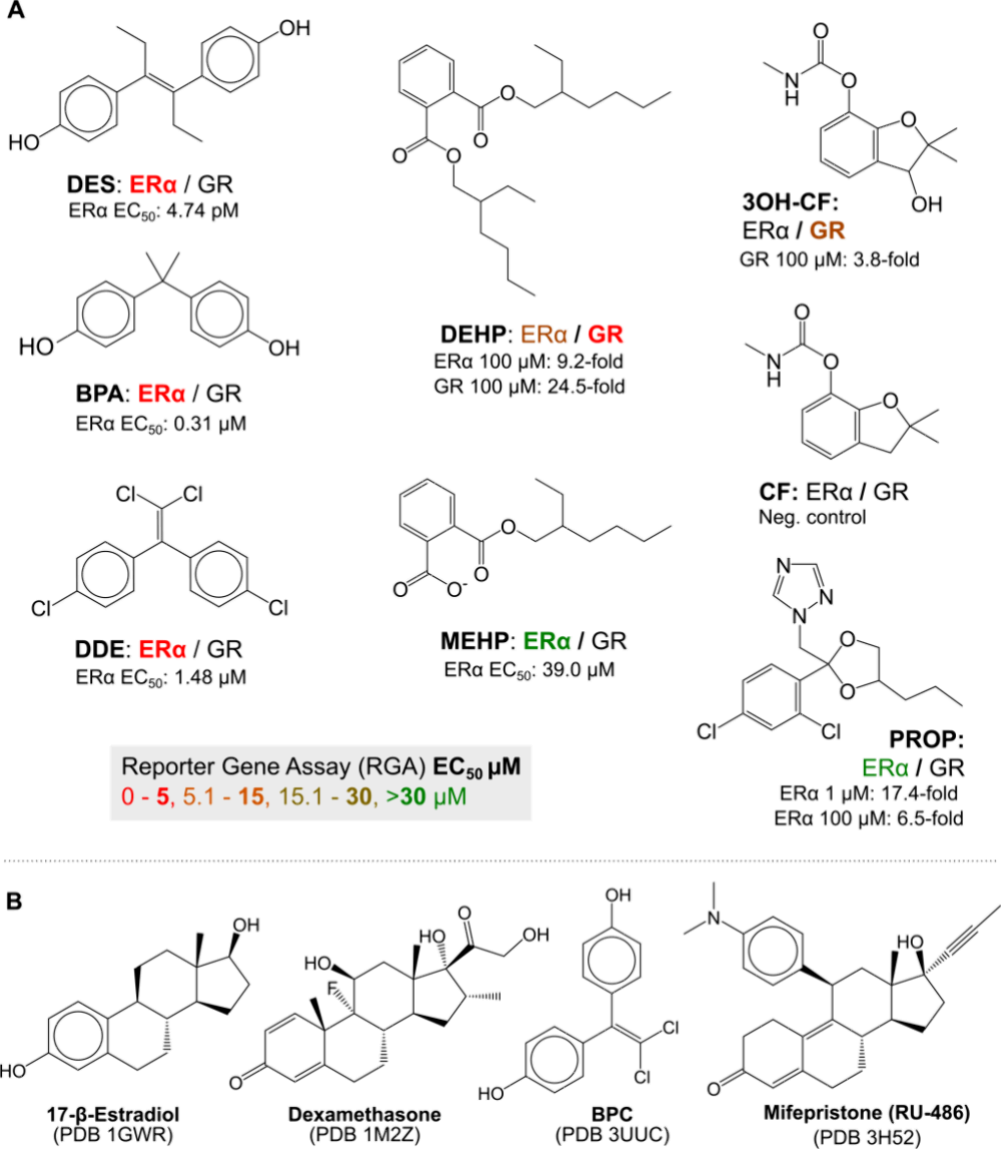
Summary of EDCs activating the ERα and GR receptors. (A) Metabolic/endocrine disruptor compounds with activity on glucocorticoid receptor (GR) and estrogen receptor (ERα) measured with a reporter gene assay. Compounds are colored by potency according to Pitkänen et al. (*in revision*). (B) Known ERα and GR agonists (17β-estradiol^7^ and dexamethasone,^8^ respectively), ERα partial agonist (BPC^9^), and GR antagonist (RU-486^10^). DES: diethylstilbestrol, DEHP: bis(2-ethylhexyl) phthalate, MEHP: mono(2-ethylhexyl) phthalate, DDE: dichlorodiphenyldichloroethylene, BPA: bisphenol A, BPC: bisphenol C, PROP: propiconazole, CF: carbofuran, 3OH–CF: 3OH-carbofuran. All CAS-Numbers can be found in the Supporting Information, Table S1.

Our group recently prevalidated SHR reporter gene assays and determined the activation of steroid hormone receptors (SHRs) by 17 suspected EDCs in HepG2 hepatoma cells (Pitkänen et al., *submitted*). EDCs that activated the estrogen (ERα) and glucocorticoid (GR) receptors at picomolar-to-low micromolar concentrations (Figure 1A) were selected for further studies in terms of their potential binding mode.

The glucocorticoid receptor regulates inflammation, immune response, and glucose metabolism^11^ and is activated by the classical anti-inflammatory drug dexamethasone (DEX, Figure 1B). ERα is regulated by 17β-estradiol (E2, Figure 1B), and it plays a major role in the growth and maintenance of the mammary gland, uterus, bone, and cardiovascular and central nervous systems. In early 2010, the ToxCast project^12^ reported that 12.2% of the investigated chemicals interacted with and/or modulated ERα activity. The later inclusion of androgen and glucocorticoid receptor assays^13^ in the ToxCast panel consolidated this triad of SHRs as a driver for further EDC studies. Regardless of the large consortia-derived studies, EDC binding to NRs was addressed on an individual basis,^14^ especially for common compounds such as bisphenols (*i.e.*, BPA, BPC, and BPS) and diethylstilbestrol (DES).

SHRs have three functional domains: (i) the unstructured N-terminal A/B domain containing the transcriptional activation function (AF-1), (ii) the DNA-binding domain, and (iii) the C-terminal ligand-binding domain (LBD), which is responsible for interactions with chemicals. The LBD exhibits a conserved three-layered α-helical sandwich fold, composed of 11 helices (referred to as H1 and H3–H12 – see Supporting Information Figure S1 for details) and up to four β-sheets.^15^ The residues forming the ligand-binding pocket (LBP) show variation among SHRs that enables them to bind diverse endogenous and exogenous ligands.^15,16^ In a simplified mechanistic model, on agonist binding in the LBP, the LBD undergoes large conformational changes, leading to the stabilization of H12 on the interface formed by H3 and H5 and generating an active state of the activation function-2 domain (AF-2) surface. The AF-2 surface mediates ligand-dependent SHR transactivation by allowing coregulator binding.^17,18^ The current mechanistic models for NR regulation are multifaceted, taking into account the diverse quaternary complexes that can appear from typical/atypical multimerization and cooperative regulatory pathways (extensively reviewed by De Bosscher and colleagues^19^). These, however, are not explored in our current work.

Different SHR-binding ligands exhibit diverse interaction profiles. For the ERα receptor, which is the focus of the study, several crystal structures, cocrystallized with agonists, antagonists, and even EDCs such as the partial agonist bisphenol, are available.^9,20−22^ They highlight the position of H12 and its helical content as key features in ERα modulation. In contrast, remarkably fewer GR structures exist that are bound to antagonist and partial agonist conformations. Only one study to date reported exploring the correlation between the ligand type and H12 behavior using molecular dynamics (MD) simulations.^23^ They generated relevant GR-LBD models in the so-called agonist and antagonist states, simulating those systems in the presence of DEX or GR antagonist RU-486. In this study, the dynamic ensemble of H12 conformations is influenced by the interacting ligand: simulations of agonistic GR-DEX display a deep minimum potential energy surface favoring a specific conformation, while antagonistic RU-486-bound GR shows a flatter landscape of potential energy and a wider amplitude of H12 conformations.^23^

Previous computational models corroborated the SHR-mediated endocrine activity based on the presence and interaction pattern of specific chemical fragments.^13,24^ However, these models relied on multiple static and short MD simulations to enhance their predictions. Our recent work on NR dynamics^25−27^ has shown that dynamic models can generate improved representations of potential ligand binding modes, which are important to consider given the flexibility of the SHR’s LBPs. Here, we focused on elucidating the binding features of selected EDCs into ERα- and GR-LBD using extended all-atom MD simulations. We compared this data set against simulations of well-known SHR ligands (Figure 1). Our findings suggest that predicted binding energy along the MD trajectories and the well-established H12 folding can be discerning features for ED binding to SHRs. We also investigated the H12-distance to the receptor core along the simulations and observed that they are highly biased toward the initial chosen protein conformation.

## 2 Materials and Methods

### 2.1 Model Generation and Structure Preparation

We modeled the systems with Maestro (Schrödinger Release 2023.2 Maestro, Schrödinger, LLC, New York, NY, 2023) and OPLS4 force-field,^28^ unless otherwise stated. Models were generated as individually specified below, and the missing side chain of inserted residues was placed using Prime, followed by loop refinement using the same software.^29^ The N-terminus, but not the C-terminus, of each model was capped. The proteins were prepared using the Protein Preparation Wizard (Schrödinger LLC, New York, NY, 2023). Missing hydrogen atoms were added, bond orders were assigned using the CCD database, and protonation states of amino acids were optimized with PROPKA (Schrödinger, LLC, New York, NY, 2023) at pH 7.4 in the Protein Preparation Wizard tool of Maestro, to select the most likely protonation states and tautomer for the histidine residues. We agreed with the software suggestions followed by optimizing the generated H-bonding species. Finally, each structure was globally minimized using the steepest descent method (cutoff: 0.5 Å for all atoms). For each combination of NR-LBD and the ligand, one representative model structure was selected for further analysis.

#### 2.1.1 ERα Models

**Figure 2:**
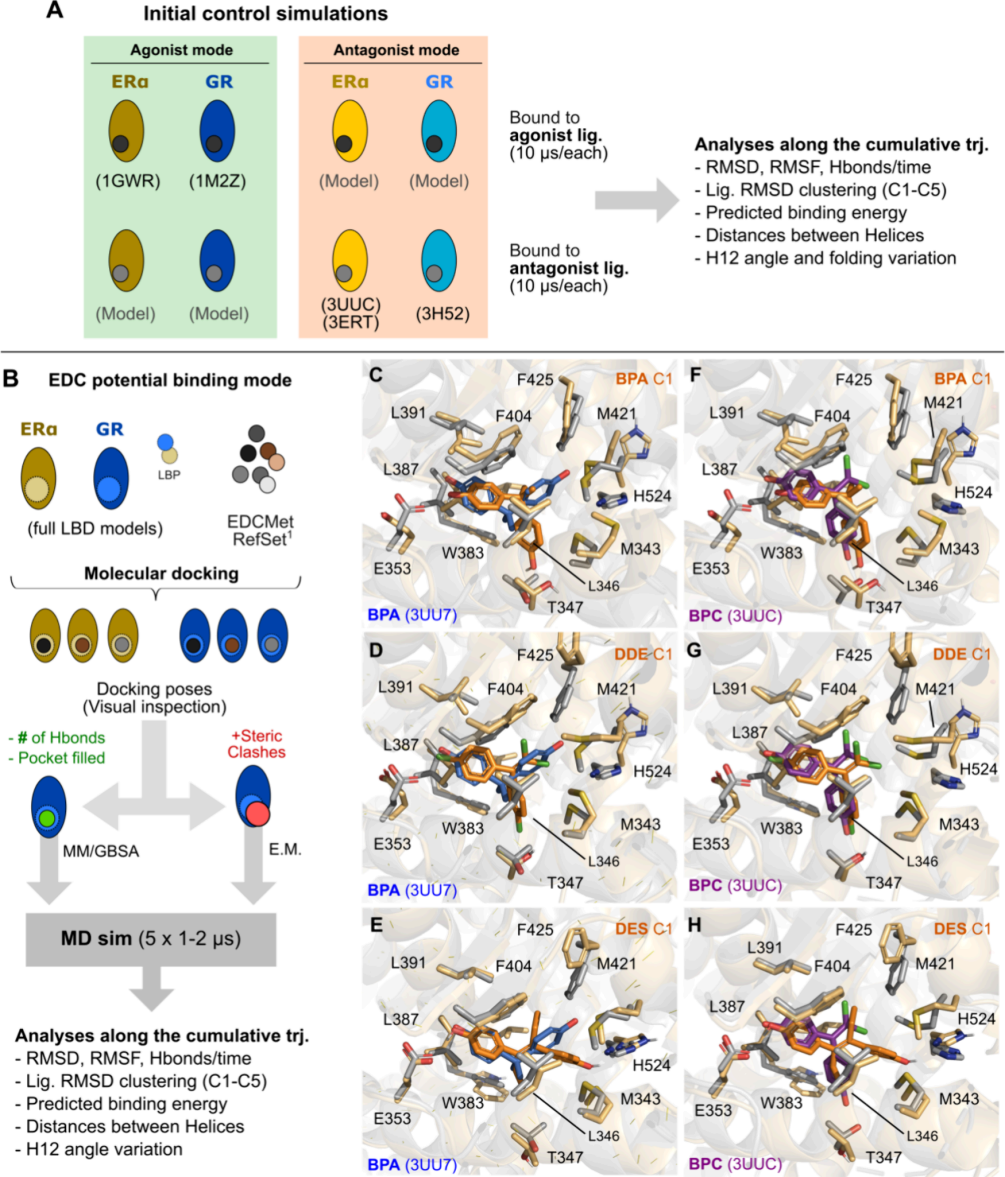
Potential binding mode generation for ERα and GR simulations. Schematic cartoon of our experiment and generated analyses for our initial control simulations (A) and EDC-prediction screening (B). Selected EDCs, illustrated in Figure 1A, belong to the so-called RefSet determined by the EDCMet consortia.^2,58^ (C–H) Representative structures (in orange) from the simulations obtained from hierarchical clustering of the trajectories (all clusters are available in Supporting Information Figures S5–S10) for BPA (C,F), DDE (D,G), and DES (E,H) in comparison to available crystal structures of known EDCs: BPA (blue, C–E) and BPC (violet, F–H).

ERα-LBD (UniProt accession code: P03372, residues: Ser301-Leu544) models were mainly generated using their agonist-bound structure (PDB ID: 1GWR(7)) as templates utilizing the Advanced homology modeling tool in Schrödinger suite (v2023.2). Other crystal structures were employed to mimic the partial agonists ERα-BPC (PDB ID: 3UUC, chain B) and ERα-BPA (PDB ID: 3UU7, only for comparison with the predicted ERα-BPA model shown in Figure 2D). The endoxifen-bound structure (PDB ID: 3ERT) was used as reference for the antagonist bound form.

H12 is often described as stable in ligand-bound state but as a flexible helix in the ligand-free state, which could be located away from the helix bundle forming the LBD.^30,31^ However, time-resolved fluorescence anisotropy experiments showed that, despite the flexibility and conformational diversity of H12 (Supporting Information, Figure S1C,D), the LBD would retain its globularity in the ligand-free state.^32^ This suggests that the complete displacement of H12 in some antagonist structures could be a result of a crystal artifact. The comparison between ERα crystal structures bound to true agonists (E2, PDB ID: 1GWR), partial agonists such as BPC (PDB ID: 3UUC; see Supporting Information, Figure S1C), or antagonists (ENDO, derived from the structural model available under the PDB ID: 3ERT) shows that distinct H12 conformations, which are still unable to bind coactivators, remain close to the LBD. The loop connecting H11 and H12 (L:H11–H12) is poorly resolved in the structures deposited under PDB IDs: 3UUC and 3ERT, containing substitutions and deletions along the H11’s C-terminal end and part of H12. We reconstructed this region as helix–loop–helix for the agonist binding conformation while keeping an extended loop for the antagonist conformation.

#### 2.1.2 GR Models

GR (UniProt P04150, residues: Ala523-Lys777) LBD models were generated using their agonist-bound structures (PDB ID: 1M2Z(8)) as templates utilizing the Advanced homology modeling tool in the Schrödinger suite (v2023.2). There are a few GR antagonist crystal structures that are only complexed with RU-486^10,33^ (Supporting Information, Figure S1A,D). In these structures, RU-486’s dimethylaniline group prevents H12 from adopting an agonistic conformation. We based our choice of modeling GR’s antagonistic conformation on the PDB ID: 3H52 (chain C, the so-called Ant3; Supporting Information, Figure S1A,D) among the other H12 conformations based on the literature.^23^ The unfolded N-terminal end (H1 and H1–H3 loops) packs itself with other monomers of the unit cell and therefore was remodeled. Previous studies with single-molecule force spectroscopy show that the H1 movement toward the LBD core is the last step in the LBD folding process, which is triggered exclusively on ligand binding.^34,35^ We used this finding to base our chimeric model where the H1–H3 helix/loop was retrieved from an agonist structure PDB ID: 1M2Z (Val258 - Arg569) while keeping the rest from the Ant3 conformation. Previous short GR simulations^23^ also showed that this designed H1–H3 loop did not suffer any substantial structural deformations.

### 2.2 Molecular Docking and Pose Selection

Before docking, ligands were prepared using LigPrep (Schrödinger, LLC, New York, NY, 2023) to assign the protonation state (Epik; at pH 7.4 ± 1.0) and the partial charges. Isomers’ chiral center configurations were retrieved from the literature using their respective CAS numbers for bis(2-ethylhexyl) phthalate (i.e., three DEHP isomers, CAS: 117–81–7) and propiconazole (CAS: 60207–90–1, which we chose to simulate *R*,*S* and *R*,*R* isomers). The other ligands were considered in terms of single isomers (Table S1).

The starting configuration for LBD-bound systems was generated using docking (Glide v7.7^36,37^), with default settings. Docking was calculated within pocket defined residues with 10 Å around E2 for ERα and DEX for GR crystallographic structures for the binding site. Docking was conducted using extra precision (XP), without any interaction restriction and keeping other options, such as van der Waals interactions, penalties for unsatisfied hydrogen bonds, and grid size as their default values. Redocking results were satisfactory displaying RMSD values of <1.5 Å. Docking of antagonist ligands in the agonist conformation of receptors, as expected, yields poor results with high scoring poses presenting several clashes, in particular with regions (i.e., H11 and H12) that would otherwise be unfolded in the experimentally determined antagonist conformations. In those cases, the best ranking scoring pose that presented a similar binding mode to the original antagonist crystal structure (i.e., positioning of the phenyl ring systems) was selected despite the clashes and underwent energy minimization using steepest descent with a large cutoff (4.5 Å).

The representative pose was selected not only based on the ranking of the best Glide docking score but also in terms of visual inspection and consistency among the different receptors and ligands, aiming for similar poses for congener ligands in both GR and ERα. Whenever possible, poses with polar groups interacting with the histidine from H12 were prioritized, given the relevance of this interaction for the mechanism. Given the length of our intended simulations, the simulation of multiple binding poses of the same ligand was not performed. Poses underwent energy minimization using the MM/GBSA prime approach (Schrödinger Maestro 2023v2^38^), allowing side chains within 4.5 Å in order to refine the energy estimates and create a relevant initial conformation to submit to MD simulations.

### 2.3 MD Simulations

We simulated the pre-equilibrated monomeric GR or ERα without the coactivator peptide in the AF-2 region with a similar protocol as described before.^25,39^ We used the Desmond MD simulation engine^40^ and the OPLS4 force-field.^28^ Ligand charges and parameters were generated for the OPLS4 directly during the system preparation using their respective force-field builder tool (Maestro2023v2). The prepared systems were solvated in a cubic box with the size of the box set as a 13 Å minimum distance from the box edges to any atom of the protein. The TIP3P water model^41^ was used to describe the solvent, and the net charge was neutralized using Na^+^ ions. The RESPA integrator timesteps of 2 fs for bonded and near and 6 fs for far were applied. Short-range Coulombic interactions were treated using a cutoff value of 9.0 Å, whereas long-range Coulombic interactions were estimated using the Smooth Particle Mesh Ewald (PME) method.^42^ Before the production simulations, systems were relaxed using the default Desmond relaxation protocol. Briefly, Maestro′s Desmond implementation has a default relaxation protocol that starts with two stages of energy minimization (backbone restrained and unrestrained) followed by four stages of MD runs with gradually diminishing those restraints and those compose an automated multistage equilibration process. It minimizes solute atoms with restraints, while solvent molecules and ions are relaxed around the solute. Next, it minimizes the full system without restraints to remove residual strain. With the NVT ensemble (constant volume and constant temperature), a short MD simulation runs at low temperature (10 K) with solute restraints. This helps to gradually heat the solvent without disrupting the solute structure. It continues gradual heating to the target temperature (310 K) under an NPT (constant pressure, constant temperature) ensemble while maintaining positional restraints. This step allows the solvent density to adjust to controlled conditions. Full relaxation under NPT conditions without restraints happens next (see www.deshawresearch.com’s Desmond manual for the details). For production, simulations were run in the NPT ensemble with a temperature of 310 K (using the Nosé–Hoover thermostat^43,44^) and pressure of 1.01325 bar (Martyna–Tobias–Klein barostat^45^). For each system, five independent simulations of at least 1 μs were carried out, with 2 μs being generated for relevant ligands, controls, and ERα systems (as mentioned in Table S1), resulting in 5–10 μs simulation data for each system. Exceptions include endoxifen systems, which ran for 25 μs (10 × 2.5 μs), and 17β-estradiol (E2), which ran for 14 μs (7 × 2 μs), aiming to allow for larger conformational changes. In total yielding ∼200 μs worth of simulation time for ERα and 100 μs for GR. Each replica was generated using the same initial coordinates but using randomly generated seed numbers for equilibration and production.

### 2.4 Analysis of MD Simulation Trajectories

#### 2.4.1 Protein–Ligand Interactions and Protein/Ligand Properties

The Maestro simulation interaction analysis tool (Schrödinger, LLC) was used for the analysis of RMSD, RMSF, and interaction analysis. We used default values for interactions that are H bonds: cutoff of 2.5 Å for donor and acceptor atoms, donor angle of 120°, and acceptor angle of 90°. For hydrophobic interactions, a cutoff of 3.6 Å was used between the ligand’s aromatic or aliphatic carbons and a hydrophobic side chain, and π–π interaction was defined as two aromatic groups stacked face-to-face or face-to-edge. For water bridge interactions, the following were used: default cutoff of 2.8 Å for donor and acceptor atoms, donor angle of 110°, and acceptor angle of 90°. For angle and distance calculations, the Maestro event analysis tool (Schrödinger, LLC) was used.

#### 2.4.2 Distances and Angle Calculations

Distances between specific secondary structure elements were calculated using their centers of mass with the Maestro script *trj_asl_distance.py* (Schrödinger LLC) trajectories. The angle between two α-helices was computed as reported earlier^23^ using a vector for each helix. The vector of an α-helix was defined by two points determined by the center of mass of four Cα atoms. For GR, residues 556–579 were chosen for the start and end points of the H3B vector, residues 727–740 were chosen for the start and end points of the H10 vector, and residues 751–764 were chosen for the start and end points of the H12 vector. For ERα, residues 538–544 represent the H12, 518–532 represent the H10, and residues 348–362 the H3B. For ERα, as an example, angles were calculated as follows:angle between H3b and the H10/H12 inflection point:
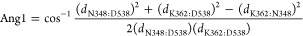
angle between H12 and H10:
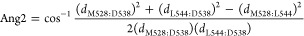
where *d* represents the Euclidean distance between the alpha carbons of the labeled amino acids.

#### 2.4.3 MM/GBSA Binding Energy Calculations

Molecular mechanics with generalized Born and surface area (MM/GBSA) predicts the binding free energy of protein–ligand complexes using Prime.^38^ The ranking of ligands based on free energy calculations can be correlated to the experimental binding affinities. However, due to the tendency of such methods to over- or underestimate absolute values,^46^ we used those predictions to observe qualitative trends and compare them against other MD-derived variables.

Our choice of using MM/GBSA instead of molecular mechanics energies combined with the Poisson–Boltzmann and surface area continuum solvation (MM/PBSA) is based on the available implementation. In addition, PB solution^47^ is suggested to be computationally time-consuming, in comparison to the more efficient approximation methods based on the GB models.^48^ Current guidelines for MM/PB(GB)SA^46^ warn about the occurrence of several uncommon conformational substates during simulations. Such cases can incur larger standard errors in the predicted binding free energy,^49^ which can be mediated by longer (maybe >10 ns) or several independent simulations, suggested to yield improved results by enhancing equilibration.^50^

In this context, the use of multiple frames from MD trajectories is encouraged for statistical convergence.^51^ However, unrealistic structures need to be eliminated to avoid incorrectly predicted binding affinities. In this sense, every 10^th^ frame from the simulations was considered for the calculations, meaning 500 and 1,000 frames for simulations of 5 and 10 μs, respectively (Supporting Information, Tables S5, S6). These were used as input files for the MM/GBSA calculations with thermal_mmgbsa.py script for the Schrödinger package. Calculated free-binding energies (kcal/mol) are represented by MM/GBSA and normalized by the number of heavy atoms (HAC), according to the following formula: ligand efficiency = (binding energy)/(1 + Ln(HAC)) for ligand efficiency. Hydrophobic terms, Coulombic terms, and hydrogen bond terms were also calculated. The hydrophobic interaction was defined based on a 3.6 Å (or shorter) distance between two hydrophobic atoms. The hydrophobic energy term from Schrödinger’s Prime MM/GBSA was calculated from a solvent-accessible surface area (SASA) calculation and the solvation model (VSGB2.1^38^).

#### 2.4.4 Chemometrics Analysis and Machine Learning Pattern Recognition

The calculated distances and other structural features from MD simulations were employed as input in principal component analysis (PCA) aiming to distinguish compounds according to their biological activity (agonist, antagonist, or inactive) following our previously published strategy.^52^ PCA was carried out using ChemoFace v.1.66 software.^53^ All descriptors were autoscaled prior to the PCA calculations. In parallel, unsupervised neural network Self-Organizing Maps (SOMs) using default parameters (10 neurons per axis and the Gaussian Neighborhood function) were carried out using DataWarrior 5.2.1 software^54^ and the same set of variables from PCA.

#### 2.4.5 Visualization and Plotting

Structural data visualization was conducted with PyMOL v.2.5.2 (Schrödinger LLC, New York, NY, USA). Data visualization was also completed by Python 3.7, seaborn (v0.12.2), matplotlib,^55^ and GraphPad Prism (v. 10.1 for Windows, GraphPad Software, San Diego, CA, USA).

### 2.5 Statistical Analyses and Image Generations

**Figure 3:**
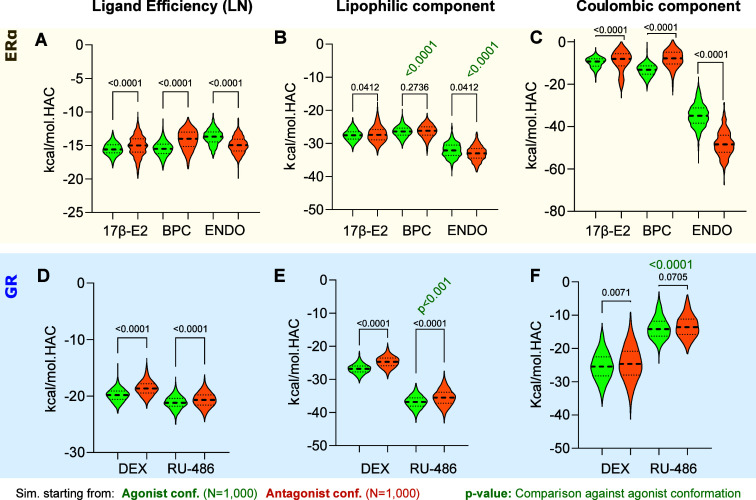
Prediction of ligand efficiency, lipophilic, and Coulombic component of binding free energy for the ERα and GR simulations. Quantification of the predicted binding energy - free energy binding calculation for each ligand along the simulated trajectory. (A, D) Violin plots depict the ligand efficiency (LN), *i.e.*, kcal/mol normalized by the heavy atom count (HAC) for ERα (A) and GR (D) as well as their lipophilic component (B, E) showing similar tendencies that are independent of the appointed normalization or method of LN calculation and their Coulombic terms (C, F). In all violin plot graphics, the median is indicated by a dashed line. Kruskal–Wallis H with Dunn’s post hoc tests were performed to compare between cumulative distribution of simulations starting from agonist conformations (green) against starting antagonist conformations (orange). Exact *p*-values are depicted when available in gray (comparisons between conformations) or in green (comparison between ligands bound to agonist conformations).

**Figure 4:**
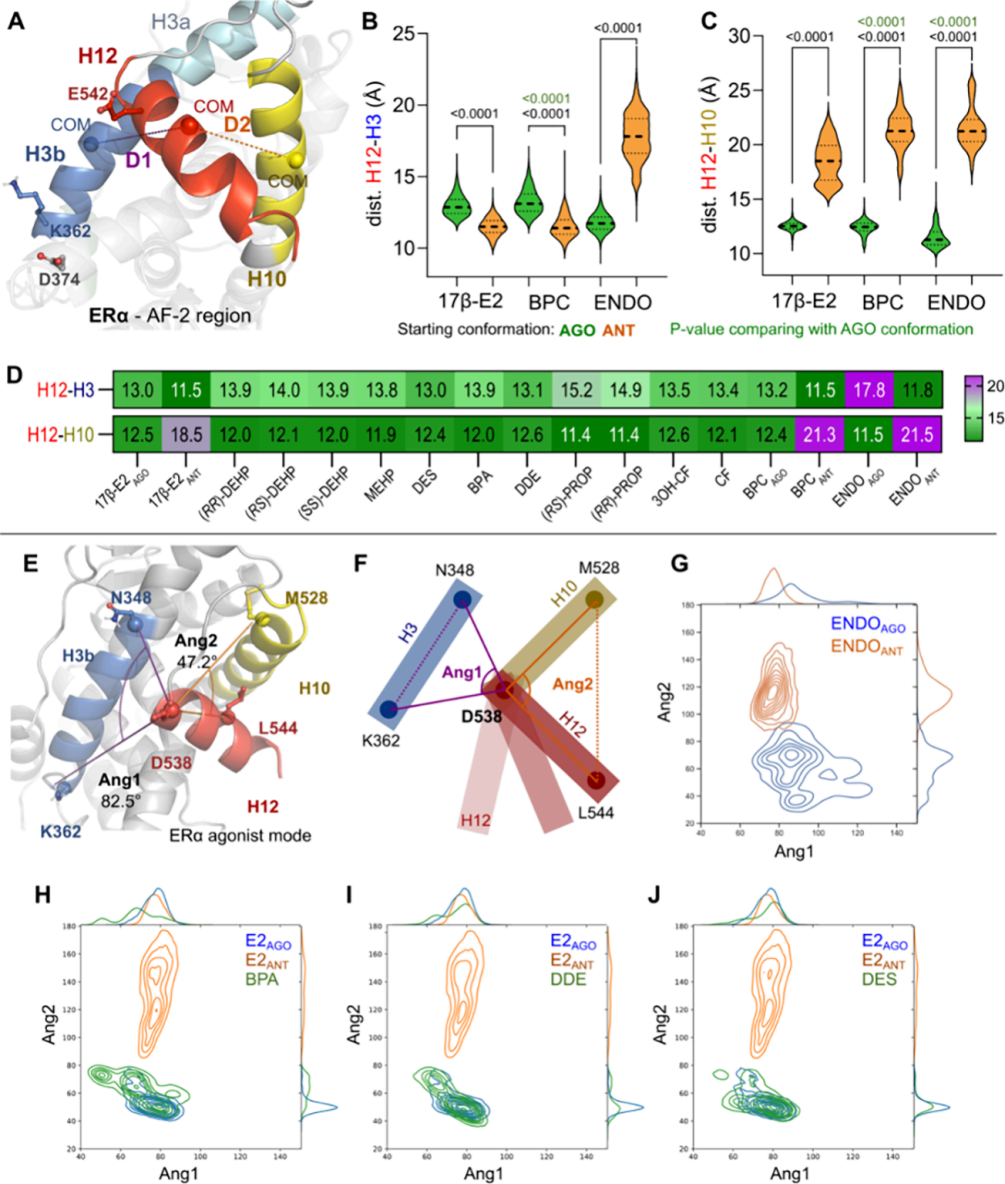
H12 folding and its distance to H10 and H3 play an important role in the SHR activation. (A) Cartoon illustration of ERα’s AF-2 region depicting the distance definitions measured in (B, C). Distance plots between relevant helices are depicted as violin plots for the H12–H3 (B) and H12–H10 (C) for ERα simulations (GR distance data are available as Supporting Information). Kruskal–Wallis tests (KW) with Dunn’s *post hoc* tests were performed to compare simulations starting from agonistic (green) and antagonistic conformations (orange) for each ligand; exact *p*-values are depicted when available as black numbers above the curves. KW tests were also performed for comparing ligands in the agonistic conformation (green vs green); exact *p*-values are depicted when available as green numbers above the curves. The median value of distances is shown as a heatmap (D). Cartoon representation (E) and illustration (F) of angles 1 and 2 (Ang1 and Ang2) monitoring the conformational changes in H12 (detailed residues and calculation formulas are described in the Supporting Information and Materials and Methods section). Variation of the Ang1 versus Ang2 along the simulations of ERα bound to endoxifen (ENDO, G) or estradiol (E2, H–J) in comparison to different EDCs (in green): BPA (H), DDE (I), and DES (J). In those angle distribution graphics, blue represents simulations starting from agonist conformations, while orange represents starting from the antagonist.

To compare the distributions of energies and distances calculated along the simulation trajectories, data was first plotted using a kernel density estimator in violin plots (Figures 3 and 4B, C). Second, the Kruskal–Wallis test followed by Dunn’s post hoc test was applied to the data. The general null hypothesis was that there is no difference. The test level adopted was α = 5%, so a null hypothesis was rejected if the *p*-value was lower than 5%. As the sample sizes go well beyond 1,000 items, the power of the statistical test is quite large, which causes even minor differences between the samples to generate statistically significant findings. This may appear to contradict the visual impression from the violin plots. However, the visualization may be biased by the smoothing properties of the kernel density estimation.

Additional insight into the data was gained by looking at box whisker plots that compare data from agonist/antagonist pairs. These show many items that are suspected to be outliers. We prepared quantile-quantile and empirical cumulative distribution functions plots (see Supporting Information for extended methods/results for statistics). These three explorative approaches revealed differences in the shape and locations for the distributions. These approaches were previously used to analyze molecular dynamics simulation data.^56,57^ GraphPad Prism 10.1 Software (GraphPad Software, Inc., San Diego, CA, USA) was used to perform statistical analysis, where p-values <0.05 were statistically significant.

## 3 Results

We aimed to investigate the influence of different EDCs in the LBD, their potential binding modes, and their affinities using a dynamic model. We generated a complete model for the ERα-LBD and GR-LBD. We systematically simulated each SHR-LBD, starting from a model with an agonist and antagonist/nonagonist conformations using established agonist, partial agonist, or antagonist ligands (Figure 2A depicts our experimental design).

These selected docking poses underwent all-atom MD simulations with explicit solvent for several microseconds. Herein, to compare the interactions and conformational dynamics of the EDCs with known SHR ligands, we conducted a total of 300 μs simulations distributed among the ERα and GR (Figure 2B and Table S1). Those trajectories underwent classical analyses such as calculating their protein backbone RMSD, RMSF, distances, and angles between relevant helices and structural elements, evaluating protein–ligand interactions and stability, and predicting NR-binding energy using generalized Born and surface area solvation (MM/GBSA).

### 3.1 SHR-EDC Stability Relies on Interactions with the Hydrophobic Subpocket and H3/H10 Anchoring Contacts

Docking of EDCs in the ERα generated poses that interacted either with Glu353 (H3) or His524 (H10), as the fragment-like ligands are either too small or too rigid to establish contacts with both or with Thr347 (H3). Figure 2C–I depicts relevant conformations in comparison with known ERα-EDC crystal structures. Whenever possible, poses with Glu353/Thr347 were prioritized. The highest-scoring docked poses often have hydrophobic contacts or pi-mediated interactions with Phe404 (β-sheets) and Trp383 (H5). This hydrophobic pocket is occupied by the phenolate moieties of BPA (PDB ID: 3UU7) and BPC (PDB ID: 3UUC), corroborating the accuracy of the initial docking poses.

Docking of antagonist ligands in the agonist conformation of receptors, as expected, yielded poor results, with high-scoring poses presenting several steric clashes, particularly in regions such as H11 and H12, which would otherwise be unfolded in experimentally determined antagonist conformations (Figure 2C). For removing the clashes, MM/GBSA was conducted on the selected docking poses.

Variation in the protein’s backbone, denoted by the RMSD, shows that most of the simulations stabilize after an initial spike (∼50 ns), suggesting that the system equilibrated over time (Supporting Information, Figures S2–S3). ERα simulations had stable RMSD_back_ < 4 Å, except for some replicas of ERα-MEHP. This suggests that despite simulating the receptors as monomers, the system can equilibrate within the evaluated time scale. Ligand RMSD values show little variation throughout the simulations (Supporting Information, Figures S2–S4), and large variations were observed in specific replicas where antagonist ligands bound to agonistic conformations, which are consistent with a longer equilibration. Among the simulated EDCs, only MEHP (in ERα) and (*R*,*R*)-propiconazole (in both receptors) had similar behavior.

Each simulation was clustered based on the ligands’ heavy atoms’ RMSD values, generating up to five clusters ranked by their occurrence within the trajectories (cutoff: 1 Å). Interestingly, the most populated cluster for most compounds does not fully match the original crystal/docking conformation (RMSD ∼ 2 Å), while the second most populated cluster (C2) does (RMSD < 1.5 Å, namely, C1, Supporting Information, Figures S5–S10 and Table S2). This observation suggests that the ligands slightly drift from their original conformation during the simulations but later find new stable conformations. For simulations displaying the most diverse ligand conformations (as seen in the clusters), we observed changes in key amino acid side chains, including flipping of Glu535 and His524 in the ERα. This was particularly evident in MEHP simulations for both receptors and in 3OH–CF for ERα, but not for GR.

Analysis of the protein–ligand interactions over the course of the simulation between ERα and EDCs or control ligands suggested that, on average, compounds formed two hydrogen bonds (H-bond; Supporting Information, Figures S11–S18 and Tables S3, S4). We confirmed ERα-E2’s key interactions with Glu353 (38% of the analyzed trajectory) and His524 (90%), when starting from agonistic conformations, whereas His524 interactions are absent in simulations starting from the antagonist conformations. ERα-ENDO simulations, starting with either agonist or antagonist conformations, form a larger number of H-bond interactions (up to 15 contacts; Supporting Information, Figure S14) but no contacts with His524. Instead, ERα-ENDO relies on stable H-bonds with Glu353 in the agonist conformation (94%) and antagonist conformation (∼70%). To a lesser extent, it also interacts with Phe404 (45–47%) in both AGO and ANT starting conformations.

In addition, ERα-BPC as our first EDC example also interacts with Glu353:33% and 53% for AGO and ANT starting conformations, respectively, with an additional stable H-bond with Thr347, exclusively on agonist conformations (81%). Among our simulated EDCs, only BPA (70%) and 3OH–CF (45%) favored interactions with Thr347. BPA also formed transient interactions with Glu353, Thr383, His524, and Leu540 for short periods during simulation (Supporting Information, Figure S12C). Interestingly, ERα-MEHP has an interaction pattern similar to ENDO simulations, where it binds to Glu353 (44%), Leu387 (23%), and Arg394 (47%), most likely due to the shared negative charge.

DEHP, DDE, CF, and propiconazole formed less stable H-bond interactions, likely due to their smaller number of HBA/HDB groups (Supporting Information, Tables S3, S4 and Appendix 1). These EDCs rely on multiple transient hydrophobic contacts (10–30% of the analyzed simulation time) such as Leu346, Ala350, Leu387, Phe404, and Leu525.

GR-EDC docking poses were selected to be as similar as possible to the ERα to ensure a direct comparison. GR simulations bound to EDCs or control molecules displayed stable RMSD_back_ < 3 Å (Figure S4), which suggests that the systems were equilibrated. RMSD inspection revealed that among the simulated EDCs only (*R*,*R*)PROP, in both receptors, had similar behavior. Clustering analysis of GR trajectories revealed flipping of Gln570, Arg611, and Tyr735 in GR-EDC simulations. We observed that this “instability” is particularly relevant for MEHP in ERα simulations.

Further, the H-bond analysis for the EDCs bound to GR suggested a higher average number of H-bonds, i.e., four to six H-bonds throughout the simulations (Supporting Information, Figures S15–S18, Table S3, Appendix 1). Notably, residue Arg611 shows up as a common interacting residue for BPA, MEHP, and RU-486 (both starting conformations), highlighting its critical role in the stability of such ligands within GR, particularly in collaboration with MEHP. Interestingly, BPA exhibited a preference for interaction with Asn564 (86%) and transient interactions with Gln570 and Phe623 for a short duration over simulation. Overall, our data provide insight into the molecular stability of EDC ligands within ERα-LBP and GR-LBP, highlighting their binding preferences and the induced conformational changes of LBP.

### 3.2 Predicted Binding Energy Supports High Binding Affinity of EDCs Comparable to Control Simulations

To investigate whether the proposed binding mode in different structures would be reflected in their predicted binding energy differences, we first evaluated our “control” simulations using MM/GBSA. MM/GBSA was chosen due to its faster speed, which allowed us to process many relevant frames from each simulation in a timely manner. We analyzed their ligand efficiency, which is the Δ*G* binding energy normalized by the number of heavy atoms, to properly compare between ligands.

For both SHRs and their respective reference ligand, ligand efficiency values are significantly different between simulations that started from agonistic conformations compared to antagonistic starting conformations (Figure 3A, D). The similarity between the hydrophobic component (Figure 3B, E; Supporting Information, Tables S5–S7) and the complete predicted binding energy not only confirms the relevance of hydrophobicity for the ERα receptor but also suggests that the hydrophobic ligand–protein interactions are not significantly affected by the conformational changes of ERα. One relevant exception is ENDO binding, which seems to be driven by Coulombic interactions (Figure 3C), consistent with its protein–ligand interaction pattern (Figure S14).

ERα simulations bound to the E2 agonist have almost indistinguishable energy levels, despite their statistical significance, for both agonist (LN: −15.5 ± 0.9 kcal/mol) and antagonist (LN: −15.1 ± 1.5 kcal/mol) conformations. Also, ERα-BPC simulations suggest that BPC would preferably bind to ERα_ANT_ (LN: −21.1 ± 1.1 kcal/mol, Table S5), with a gap of at least ∼6 kcal/mol to the ERα_AGO_ simulations (LN: −15.5 ± 1.1 kcal/mol). In GR (Supporting Information, Tables S6–S7), there are different patterns. In agreement with the reported DEX agonistic activity, GR-DEX simulations revealed a lower ligand efficiency (more negative) in the GR agonistic (LN: −20.1 ± 1.2 kcal/mol) conformation, suggesting a more stable binding when compared to its antagonist conformation (LN: −18.6 ± 1.3 kcal/mol, Δ*G* gap ∼ −1.47 kcal/mol). RU-486 lies somewhere in between (Δ*G* gap ∼ 0.48 kcal/mol) with agonistic and antagonist conformations displaying LN: ∼ −20 ± 1.2 kcal/mol. Comparison between the binding energy mean values (Supporting Information, Tables S5–S7 and Figure S19) shows that the GR agonist has lower energy and, therefore, better binding affinity for GR_AGO_ than GR_ANT_ (Δ*G* gap ∼ 5 kcal/mol), whereas RU-486 seems to bind equally effectively in either conformation.

Though it has been suggested that even small differences in binding energy may impact experimental binding constants,^59^ one cannot disregard the effects of the errors in our own predictions since MM/GBSA has been shown to trade-off accuracy by speed (see Materials and Methods). Despite that modest distinction observed in the SHR-“control” simulations, we proceeded with the EDC studies to classify them between those two classes. Most of the simulated ligands’ energy values fall into skewed multimodal distributions (Supporting Information, Figure S19), highlighting a higher frequency of lower energy values. For EDCs, the LNs also seem to be driven by their lipophilic component (Supporting Information, Tables S5, S6).

As an illustrative example, the contrasts between binding energies for ERα_AGO_ simulations bound to different EDCs allow comparisons among some congeners. The different DEHP isomers are indistinguishable in terms of binding energy. They, however, bind with higher affinity (LN: ∼ −19.6 ± 1.2 kcal/mol, values from ERα simulations) than MEHP (LN: ∼12 ± 1.9 kcal/mol). ERα-MEHP displays a large variance for the predicted binding energy and its Coulombic term (SD = 9 kcal/mol, Table S7), which is not only a result of large SD for each replica (ranging from 8.4 to 11.2 kcal/mol) but also a consequence of two outlier replicas with abnormal mean values (replicas #2 LN: 21.98 ± 11.19 kcal/mol and #3 LN: −4.84 ± 16.54 kcal/mol). Ignoring those replicas during the energy calculation results in LN: −12.92 ± 0.64, which is within the initial values’ range. Our cell-based ERα-RGA results point DEHP to be a moderate agonist (see Figure 1) and MEHP to be a poor agonist (ERα EC_50_ 39 μM). In addition, cell-free binding results show moderate and poor interactions with ERα by DEHP and MEHP, respectively,^14^ corroborating that MEHP’s poor agonism is not a result of cell-permeability problems.

As a second example, where MM/GBSA poorly correlates with biological data, we analyze the diphenyl series (DES, BPA, and DDE). Our ERα-DES simulations show the worst ligand efficiency values (LN: −15.1 ± 1.3 kcal/mol) within this series, being outperformed by DDE (LN: −17.7 ± 0.99 kcal/mol), which is inconsistent with ERα’s reporter gene assay results (Figure 1). Literature data on cell-free ERα’s [^3^H]-E2 competition assays confirm the synthetic estradiol analogue: DES as an exceptional binder (IC_50_ 2.25 nM) and BPA as a moderate binder (IC_50_ 11.7 μM).^14^ This is also consistent with literature results for glucocorticoid radioligand binding (ligand: [^3^H]-DEX, see DrugMatrix at CHEMBL1909046 documents) for DES (EC_50_ 10.6 μM) and less conclusive (unconfirmed) qHTS assays for DDE (EC_50_ 22.4 μM) and BPA (EC_50_ 398.1 nM).

Last, the fact that our negative control CF and its metabolite 3OH–CF show a higher predicted binding energy in GR than in ERα (Supporting Information, Figure S19) may be related to our finding that the 3OH–CF metabolite is active in the GR reporter gene assay but not in the ERα. In contrast, CF remains inactive in both reporter gene assays since it does increase coactivator recruitment (Pitkänen et al., *submitted*) and has poor binding on cell-free assays.^14^

### 3.3 H12 Folding, H12-Related Distances, and Angles Can Be Monitored as a Distinctive Feature for SHR Activation

Given H12’s crucial role in composing the coregulator binding surface, we investigated its stability in both ERα and GR to closely monitor the role of this helix in the systems’ structural transitions.

#### 3.3.1 ERα’s H12-Composed Movement Can Offer Insights into EDC Binding

Quantification of the ERα’s H12 secondary structure elements (SSE%; Supporting Information, Figure S20), which indicates the extent of H12 folding, confirms that H12 remains folded in a longer portion of simulations with agonists than with antagonists. On ERα, simulations with E2 and BPC starting from agonistic conformations retained H12 folding and, alternatively, recovered an agonist-like folding when starting with the antagonistic conformations. This prompted us to investigate the variation of H12 conformation, using distance and angle, toward LBD elements.

Simulations of both SHRs starting from agonistic conformations displayed low RMSF median values in the helices H3′s C-terminus (i.e., H3b) and H10 (Supporting Information, Figures S21–S24). Indeed, H3′s C-terminus (RMSF values between 0.86 ± 0.1 and 1.59 ± 0.7, for E2_AGO_ and E2_ANT_, respectively, as the lowest and highest observed values) and H10's C-terminus (RMSF: 0.90 ± 0.15 to 1.1 ± 0.15, for E2_AGO_ and BPC_ANT_, respectively, as the lowest and highest observed values) can be anchors for distance calculations toward H12 along the simulation. We then calculated changes in the distance among the relevant pairs of helices H3, H10, and H12 (Figure 4A–C; Supporting Information, Table S8 and Figure S25). Although all comparisons show significant differences between simulations starting from agonist and antagonistic conformations, only a couple of systems display meaningful changes in terms of their means and standard deviations.

On the one hand, the large H12–H3b distance in ERα (Figure 4B) recapitulates the well-established H12 flexibility on ENDO simulations that started from antagonistic conformations, but surprisingly, simulations starting from the agonist conformation did not show the same movement amplitude (Supporting Information, Figure S25A). On the other hand, the distance H12–H10 in ERα greatly depends on the starting conformation (Figure 4C). Specifically, H12 remains close to H10, independently from the bound ligand, when starting from agonistic conformations (Figure 4C; Supporting Information, Figure S25C; Table S8: SD ∼0.4–0.1 Å) but varies moderately when starting from antagonistic conformations (SD ∼ 1.8 Å) with a shift of 3–4 Å between those two populations. This lack of relevant changes is consistent in the ERα-EDC simulations, where we observe on average <2.5 Å variation between the different ligands for both H12–H3 and H12–H10. Last, the H10–H3 distance (Supporting Information, Figure 25E) is stable for ERα displaying little variation regardless of the ligand or starting conformation. The only exception is ERα-ENDO (dist. H10–H3 > 3 Å) with the antagonist starting conformation.

Inspired by the work of Alvarez et al.,^23^ we decided to monitor the angle variation between H12, H10, and H3b (Figure 4E,F; Supporting Information, Figure S26). Visual inspection of the angle 1 (Ang1) variation, represented by the shift of H12 in relation to H3b, in ERα-control ligands shows a clear separation between agonists (70°) versus antagonists (80–120°), independently from the initial starting conformation (Supporting Information, Figure S26E–G). Meanwhile, angle 2 (Ang2, representing the H12 opening away from H10) shows a more pronounced separation between the different starting conformations (Figure 4G–J). While agonist-starting simulations retain a stable and smaller angle (50–60°), simulations starting from antagonist conformations demonstrate a larger angle with broader variation (100–170°). The variation amplitude of those two angles is a distinctive feature for EDC classification, where our most potent ERα ligands displayed smaller variations comparable to ERα-E2_AGO_ simulations (Figure 4H–J and Figure S27). For instance, BPA, DDE, and DES’ Ang1 histograms display single stable peaks around 60°, whereas MEHP, CF, and propiconazole have multiple peaks ranging from 40 to 80°, suggesting H12-instability.

#### 3.3.2 GR’s H12 Conformation Flexibility Can Indicate Antagonism but Not Classify EDC Binding

GR’s H12 folding status (SSE%) during simulations, in contrast to ERα, is less variable, meaning simulations with either DEX or RU-486 retain their initial H12 folding according to the starting conformation and not the ligand activity (Supporting Information, Figure S20). All distance changes in GR simulations were not as expressive as for ERα; for instance, the H12–H3b distance in GR remains (Supporting Information, Figure S25B), on average, higher only in simulations bound to the RU-486 antagonist, which implicates a rearrangement to accommodate the ligand. Changes in H12–H10 and H3–H10 distances are independent of the bound ligand when starting simulations from the agonistic conformation, with the only changes observed in the RU-486_ANT_ simulations (Supporting Information, Figure S25D,F), demonstrating H12 collapse/displacement consistent with their high RMSF values.

Changes in the Ang1 and Ang2 in GR bound to DEX or EDC simulations are consistently modest (Supporting Information, Figures S26 and S28), displaying a tight distribution of less than 10° around their averages Ang1 ∼65–68° and Ang2 ∼45–50°. In contrast to ERα, this suggests that Ang1/Ang2 would not be a good distinctive feature for GR-EDC classification. RU-486 is an exception in either starting conformation, presenting large shifts in the H12 angles and great overlap between simulations starting from agonist or antagonist conformations. RU-486_AGO_ histograms for Ang1 present a major distribution around 60–70°, while RU-486 simulations starting from antagonist conformations have two main peaks at 45° and 70°. This suggests that GR antagonists would have a tighter angle between H12 and H3 (40°) in comparison to agonists (DEX Ang1: 60°).

### 3.4 Unbiased Selection of MD-Derived Metrics for EDC Classification

We then employed principal component analysis (PCA) to (i) evaluate which MD-derived variables could better distinguish agonists from antagonists independently from the starting conformations and (ii) classify potential EDCs accordingly. PCA was generated using variables derived from the MD trajectories (Supporting Information, Tables S3–S9) including protein–ligand interaction, RMSF, distances between secondary structure elements, folding, binding energy, and ligand properties (Supporting Information, Table S9).

**Figure 5:**
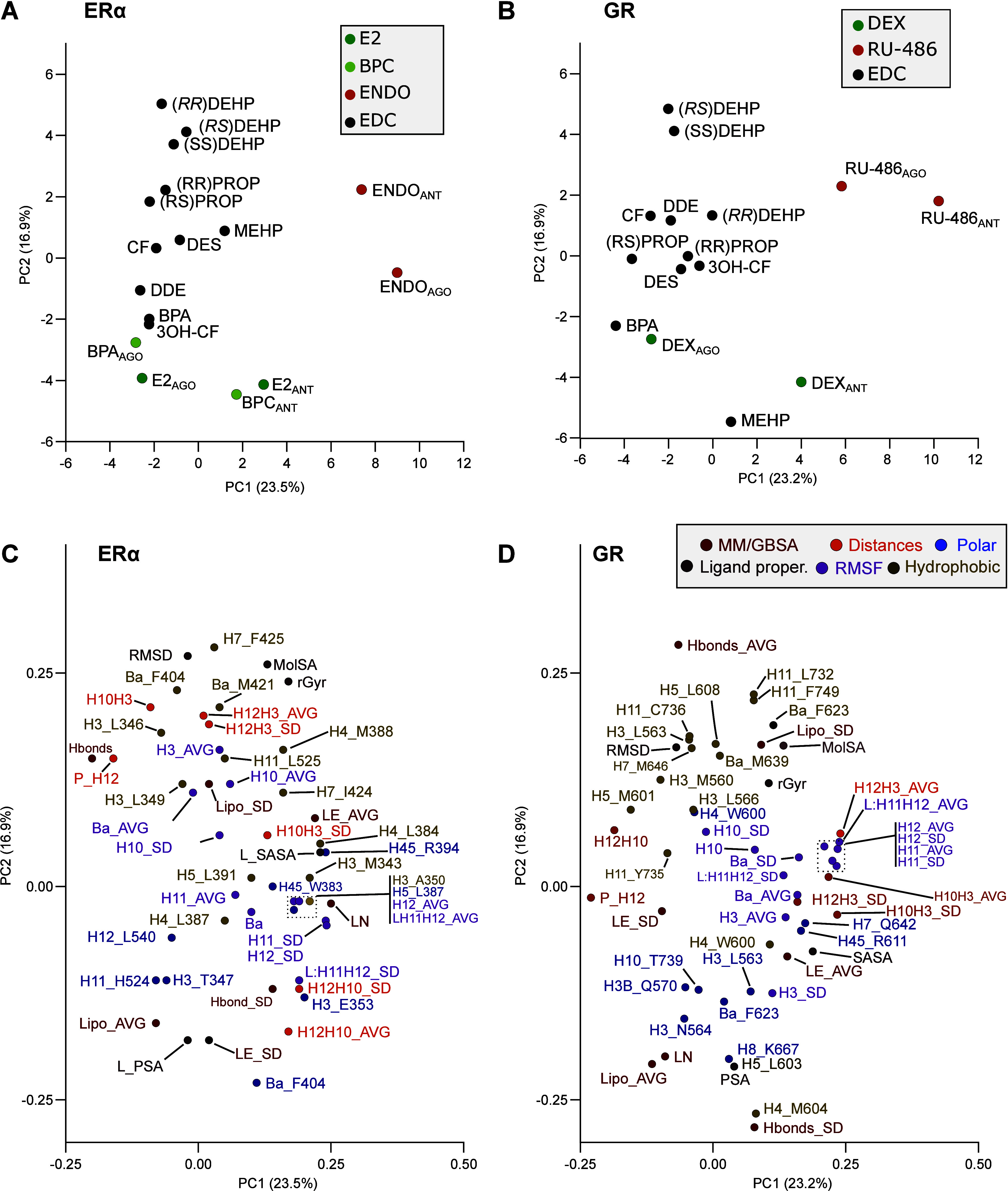
PCA classifies EDCs together with known SHR ligands. PCA classification of EDCs together with known ligands based on information derived from MD trajectories for ERα (A) and GR (B). PCA components’ variable relevance for ERα (C) and GR (D). Properties are described in the respective Supporting Information tables along with this document. βa denotes the folding of the beta-sheet alpha.

The first principal component (PC1:23.5%) for the ERα simulations separated the antagonists from a group containing the agonists, partial agonists, and potential EDCs (Figure 5A). PC1 is composed of contributions of H12’s folding (SSE%) and flexibility (RMSF) as well as hydrophobic and polar contacts (Supporting Information, Table S10) with Leu384 (H4, hydrophobic), Leu387 (H4, polar), and Arg394 (H5, polar). Reassuringly, the high degree of H12 folding and its low flexibility characterize agonists and partial agonists, while the high frequency of the hydrophobic contacts (Met343, Leu384, and Leu387) tends to describe antagonists (Figure 5C,D; Supporting Information, Table S10). The EDCs are mostly separated along the second PC axis. High values of Phe404 (β-sheet) and Phe425 (H7), together with ligand-derived molecular surface and radius of gyration, are important to classify the most potent ligands. Interestingly, according to this classification, MEHP seems to be the most “antagonist-like” compound behaving distinctly from DEHP and other EDCs.

Meanwhile, a similar approach using variables derived from the GR trajectories (Figure 5B) confirms that PC1 (23.2%) is responsible for distinguishing agonists from antagonists. The interpretation of the relevant variables for PC1 (Figure 5D and Table S10) recapitulates the relevance of H12, where the shorter distance between H12–H3 and the low flexibility of the H11, L:H11–H12, and H12 region (RMSF) characterize agonist binding. In agreement, PC2 (16.9%) accounts for the influence of hydrophobic interactions from Leu732 (H11), Cys736 (H11), and Phe749 (H11), which are more frequent among DEHP, RS, and SS isomer simulations.

Finally, self-organizing map (SOM) clustering for ERα data set corroborated the PCA classification (Supporting Information, Figure S29). Antagonists formed a single group, potentially because of the initial conformation of the receptor, whereas agonists, partial agonists, and EDCs were grouped within a high-probability cluster. For the GR data set, the interpretation of SOM clustering is similar to the PCA though less informative: agonist samples were predicted to be close to each other within a low probability barrier, while antagonist samples were separated.

## 4 Discussion

SHRs can interact with many EDCs.^2^ ERα has been experimentally shown to interact with several EDCs within our RefSet chemical list (Pitkänen et al., *submitted* and Figure 1), as indicated by reporter gene assay results, and with others reported in the literature.^60^ However, GR is understudied as a receptor for EDCs,^61^ and it was activated in our reporter gene assays by only two EDCs. In this work, we investigated the theoretical binding mechanism of selected EDCs to ERα and GR and compared their conformational changes with well-known agonists and antagonists. Previous QSAR studies,^24^ despite their low computational cost, could not effectively predict active versus inactive compounds from large chemical sets. Additional studies using docking to evaluate protein–ligand interactions suggest that highly polar EDCs would rely on specific H-bonds (Glu353, Arg394, and His524) for recognition by ERα.^24,62^ However, small fragment molecules (MW < 150 Da and <10 heavy atoms) with little to no H-bond donor/acceptor groups would fail in this docking classification setup.

We hypothesized that simulations could be used to evaluate the SHR-EDC recognition, which would be reflected not only in the protein–ligand interaction frequency and predicted binding energy but also in the dynamic conformational change. Our toxicologically relevant EDCs have a limited number of H-bond acceptors/donors (Supporting Information, Table S9) and are highly flexible (Table S9), which supports this distinct approach. We initially studied simulations of known agonists, partial agonists, and antagonists of ERα and GR to verify whether simulations could reproduce relevant conformational changes and structural features. We then proceeded to use those features for classifying the selected EDCs.

MD simulations have been applied to SHRs in the literature. Shorter simulations (20 ns) starting from a ligand-free conformation of ERα could establish the process of helix displacement, which was classified as disordering.^24^ In addition, previous work using steered dynamics for GR,^23^ oriented by the protein backbone carbon RMSDs within H3, H11, and H12 as collective variables, demonstrated that those helices work as conserved secondary structure elements, only changing orientation among themselves without deformation. Thus, those structural elements were reported as collective variables or as anchors to characterize the overall GR conformation,^23^ and therefore, we here expanded this approach to ERα.

In terms of classical distance measurements (H12 to H3 and H10), only a few of our “control” simulations display relevant changes. In some cases, the visual plots reveal that there is a shift in location (*e.g.*, H12–H3 in ERα), while in others, different shapes of distributions are revealed with almost identical median values (*e.g.*, H10–H3 in ERα). The application of statistical tests is problematic due to the large samples that render even small differences between samples to be statistically highly significant. On the one hand, the known SHR crystal structures illustrate that even modest changes in these distances contribute to larger conformational changes in simulations with longer time scales. On the other hand, we discourage the use of H12 distance measurements as the single metric for classifying new ERα ligands. Meanwhile, we advise the study of H12-related angle variation in comparison with relevant control ligands. In addition, we confirm that the helical folding of H12 is a distinctive factor for activation/inactivation simulations.^63^

One can argue that our EDC list does not contain sufficiently strong antagonists to trigger such conformational changes. This is also consistent with the low number of EDCs acting as GR agonists (Pitkänen et al., *submitted*). However, when considering the imposed conformational changes induced by ENDO and RU-486, in our control simulations starting from antagonistic conformations, we cannot disregard the bias introduced by the choice of initial structures.

The above observations are not limited to ERα or GR. For instance, the high-resolution structure of progesterone receptor (PR) complexed with the antagonist RU-486 shows a high flexibility but not a full displacement of the H12.^64^ Given the crystallization procedure that started from an agonist-bound crystal that was later soaked with RU-486, one can postulate that antagonist binding is a dynamic equilibrium process. This PR agonist-to-antagonist transition indeed could not be captured by unbiased MD simulations on a low microsecond (∼6 μs) scale.^65^ By contrast, in simulations of the ligand-free GR structure, H12 conformations are more flexible and resemble the GR_ANT_ bound to RU-486, presumably describing an intermediate state between the antagonistic and agonist states. This transition between distinct H12 structural conformations was experimentally observed with the peroxisome proliferator-activated receptor gamma (PPARγ) behaving as a dynamic ensemble of conformations.^66^ On the one hand, the coregulator surface of ligand-free and partial agonist-bound PPARγ is characterized by multiple thermodynamically accessible conformations. On the other hand, agonist- and inverse agonist-bound PPARγ displays only a few restricted conformations that favor coactivator or corepressor binding, respectively.^66^ Alternatively, previous SPR studies showed that the metabolites MEHP/MEOHP, but not the parental compound DEHP, could bind PPARγ.^67^ Their binding is explained by ligand engagement with H3–H4 and H6–H7, as monitored by HDX-MS, which is distinct from ERα/GR activation that is more reliant on the H11/H12.

In terms of predicted binding energy, our results show a clear gap for GR reference ligands, where agonists and antagonists can be distinguished by their mean binding energy. However, the same is not true for ERα, whose simulations with E2 have undistinguishable energy levels between starting conformations and poor correlation between the experimentally determined EC_50_s and energies (Supporting Information, Figure S30). Interestingly, our simulations suggest that BPC would bind with higher affinity to ERα_ANT_ (Δ*G* gap ∼ 6 kcal/mol), consistent with its proposed antagonism.^9^ In this sense, we report that MM/GBSA energy predictions strongly depend on the initial structure, even after long simulations.

In both GR and ERα, the use of all MD-derived descriptors could separate EDCs based on H12 folding and conformation (angles) and hydrophobic interactions, which is an important addition to the previously identified H bond-based interactions with Glu353, Leu387, Arg394, and His524 emerging from unsupervised HCA studies^24^ to classify agonists. Comparative analyses on SHRs explored the extrapolation between NRs.^68^ For instance, relevant interactions such as the polar contacts to ERα Glu353 are available for GR with a different H-bond nature due to its different amino acid composition (GR: Asn564, Gln570, Arg611, Glu642, Thr739, Supporting Information, Figures S5–S18 and S31). The GR-LBP is lined by residues with shorter side chains than that of ERα, rendering it more spacious. For instance, the deacylcortivazol, a potent glucocorticoid, binding to the GR-LBD results in the expansion of the pocket volume from 570 Å^3^ (in the presence of dexamethasone) to 1070 Å^3^, which shows the highly flexible and adaptable GR-LBP.^69^ This could be the result of fewer steric constraints and in turn more room for movement, which makes the LBP more flexible. This property allows GR to bind to structurally diverse ligands compared to ERα. In contrast, bulkier amino acids introduce steric hindrance but form more stable interactions.

## 5 Conclusions

Our findings suggest that purely biophysical MD-derived descriptors, such as hydrophobic contacts and geometrical changes, can identify novel SHR agonists. However, unless the ligands are highly potent, one might be at the mercy of the bias introduced by the initial protein templates. We suggest that a setup to identify and distinguish novel compounds should include different starting conformations (agonist- and antagonist-bound initial structures) and comparisons against established reference ligands. We also ill-advise the use of energy-based predictions (e.g., MM/GBSA or MM/PBSA) or distances between secondary elements (e.g., H12–H3b) as unique distinctive features, moving rather to composed variables such as protein–ligand interactions and helical movements (here represented by changes in angles). Since MD simulations are a high-end resource-intensive technique, they can be used in a pipeline when other ligand-based options, such as classical QSAR and ML-based classification models, are already exhausted to provide structural insights for known binders. Indeed, simulations of just LBD with different ligands represent a fraction of the entire process. Our experience comparing the predicted binding energy of MM/GBSA and the radiolabeled ligand displacement, retrieved from the literature, has yielded interesting observations within the congener’s pairs. Although our MD simulations enhance our understanding of ERα and GR conformational changes, it is indeed known that NR behaves in a tissue-specific manner, able to regulate different sets of genes in different organs based on the transcriptional complex they form. This leads to distinct physiological consequences. When studying an NR project, it is important to notice that NR also undergoes various modifications such as epigenetic, ubiquitination, etc., which should be considered when investigating NR mechanistic and dynamic behavior. Nevertheless, we highlight that combining computational technologies with experimental testing (present in our cosubmitted paper) and validation can help in understanding the molecular initiation events, which is one relevant aspect of the behavior of ERα and GR. We should be aware that, though computational approaches provide valuable insights, they do not ensure certainty in their predictions.

## Data Availability

Prepared structures, MD trajectories, MD simulation configuration and parameter files, as well as raw and processed data for SHR–ligand interactions, are available in the Zenodo repository (under the DOIs: 10.5281/zenodo.8188646, 10.5281/zenodo.8181634, 10.5281/zenodo.11142764, 10.5281/zenodo.14750710, and 10.5281/zenodo.8181630, available upon publication). Third-party software programs employed in the manuscript include GraphPad PRISM version 10.2 (https://www.graphpad.com/), Schrödinger Suite 2023.1–2024.1 (https://www.schrodinger.com), and PyMOL version 2.5.2 (https://pymol.org/), which are distributed under respective licenses. PCA was carried out using ChemoFace v.1.66 software, and Self-Organizing Map (SOM) analyses were carried out using DataWarrior software, both available free of charge.

## Supporting Information Available

Creating and interpreting plots for statistical analyses (PDF)
SHR–ligand H-bond interactions (ZIP)
Homology model, summary of simulated systems, RMSDs and RMSFs, potential binding mode studies, population frequency clusters, timeline visualization results, protein–ligand interactions, predicted binding energies, Coulombic energy terms, ligand efficiency prediction, H12 folding, distance plot between helices, H12 relevant angle variation, Ang1 and Ang2 variations, mean and standard deviation values, ligand properties averaged along the simulation time, fractional contribution, SOM, correlation results, amino acids with relevant interactions on SHRs-EDCs, and methods and statistical results (PDF)

## ABBREVIATIONS

EDC: endocrine-disruptor chemical
ER: estrogen receptor
FDA: Food and Drug Administration
GR: glucocorticoid receptor
EC_50_: half-maximal effective concentration
LN: ligand efficiency
LBP: ligand-binding pocket
LBD: ligand-binding domain
MDC: metabolism-disruptor chemical
MD: molecular dynamics simulations
MM/GBSA: molecular mechanics with generalized Born and surface area solvation
NMR: nuclear magnetic resonance
PCA: principal component analysis
RMSD: root-mean-square deviation
SHR: steroid hormone receptor
SD: standard deviation
