# Supplementary material for: Look What You Made Me Do: Discerning Feature for Classification of Endocrine-Disrupting Chemical Binding to Steroid Hormone Receptors
Source: J Chem Inf Model. 2025 Apr 9;65(8):4148–62. doi: 10.1021/acs.jcim.4c02288 (PMC12042260; doi:10.1021/acs.jcim.4c02288)
Supplement: Supplementary file 1 — ci4c02288_si_001.pdf [file ci4c02288_si_001.pdf]

Explorative Data Analysis of Data from *Structural features of endocrine-disrupting chemicals (...)*

Ulrich Schoppmeier

2024-08-13

Contents

1 Creating and Interpreting the Plots. 5

1.1 Creating the Plots. 5

1.2 Interpreting the Plots 8

Chapter 1

Creating and Interpreting the Plots.

1.1 Creating the Plots.

This short documentation gives a description of the additional plots used for the analysis of data. Three types of plots were made

- box whisker plots
- quantile-quantile plots
- empirical cumulative distribution plots

Data were read from an Excel file using the `readxls` routine from the `readxl` package (cf. Wickham and Bryan [2023]). All the *R* functions are from the `base` package (cf. R Core Team [2023]). Additional packages were used to create this document (cf. Xie [2023a], Xie [2023b], Allaire et al. [2023], Xie [2016], Xie [2015], Xie [2014], Xie et al. [2018] and Xie et al. [2020]).

We give her the R code used. It reads data from the Excel file and creates plot, which are saved to files.

```
library(readxl)

##wd <- "Q:\\NAS\\Drahel\\Thales\\automatedPlots\\"
wd <- "Q:\\Thales\\automatedPlots\\"
sheet_names <- excel_sheets(paste(wd,"summaryOfData.xlsx",sep = ""))
sheet_numbers <- 2:12

actual_sheet_number <- 2
for(actual_sheet_number in sheet_numbers){
```

```
sample_1 <- read_xlsx(paste(wd,"summaryOfData.xlsx",sep = ""),
                      sheet = actual_sheet_number,
                      range = "A6:A20000")

sample_1 <- as.vector(unlist(sample_1))
sample_1 <- sample_1[-which(is.na(sample_1))]


name_1   <- read_xlsx(paste(wd,"summaryOfData.xlsx",sep = ""),
                      sheet = actual_sheet_number,
                      range = "A1")


sample_2 <- read_xlsx(paste(wd,"summaryOfData.xlsx",sep = ""),
                      sheet = actual_sheet_number,
                      range = "B6:B20000")
sample_2 <- as.vector(unlist(sample_2))
sample_2 <- sample_2[-which(is.na(sample_2))]


name_2   <- read_xlsx(paste(wd,"summaryOfData.xlsx",sep = ""),
                        sheet = actual_sheet_number,
                        range = "B1")


actual_sheet_name <- sheet_names[actual_sheet_number]

pdf(paste(wd, actual_sheet_name, ".pdf", sep = ""),
    width = 4, height = 7)
par(mfrow = c(3,1))
 # qq-plot
  xlim_low  <- min(sample_1,sample_2, na.rm = TRUE)
  xlim_high <- max(sample_1,sample_2, na.rm = TRUE)
  qqplot(sample_1,sample_2, type = "l",
         main = "Q-Q-Plot\n diagonal in blue",
         xlim = c(xlim_low,xlim_high),
         ylim = c(xlim_low,xlim_high),
         xlab = colnames(name_1[1,1]),
         ylab = colnames(name_2[1,1]))
  abline(0,1, col = "blue")

  # Box-Whisker-Plot
  xy <- data.frame(data = c(sample_1,sample_2),
                   source = c(rep(colnames(name_1[1,1]),length(sample_1)),
                              rep(colnames(name_2[1,1]),length(sample_2))))
```

```
    boxplot(data ~ source,
            data = xy, xlab = "", ylab = "")

    # ecdf
    ecdf_1 <- ecdf(sample_1)
    ecdf_2 <- ecdf(sample_2)

    xlim_low  <- min(sample_1,sample_2, na.rm = TRUE)
    xlim_high <- max(sample_1,sample_2, na.rm = TRUE)
    plot(ecdf_1, verticals=TRUE, do.points=FALSE, col = "blue",
         main = "Empirical Density Functions", ylab = "edf(x)",
         xlim = c(xlim_low,xlim_high))
    plot(ecdf_2, verticals=TRUE, do.points=FALSE, add=TRUE, col='brown')
    legend(x = "topleft",
           legend = c(colnames(name_1[1,1]),
                      colnames(name_2[1,1])),
           text.col  = c("blue","brown"),
          lty = 1, col = c("blue","brown"),lwd = 2)
  dev.off()

  jpeg(paste(wd, actual_sheet_name, ".jpeg", sep = ""),
       width = 4, height = 7, units = "in", res = 600)
  par(mfrow = c(3,1))

  # qq-plot
  xlim_low  <- min(sample_1,sample_2, na.rm = TRUE)
  xlim_high <- max(sample_1,sample_2, na.rm = TRUE)
  qqplot(sample_1,sample_2, type = "l",
         main = "Q-Q-Plot\n diagonal in blue",
         xlim = c(xlim_low,xlim_high),
         ylim = c(xlim_low,xlim_high),
         xlab = colnames(name_1[1,1]),
         ylab = colnames(name_2[1,1]))
  abline(0,1, col = "blue")

  # Box-Whisker-Plot
  xy <- data.frame(data = c(sample_1,sample_2),
                   source = c(rep(colnames(name_1[1,1]),length(sample_1)),
                              rep(colnames(name_2[1,1]),length(sample_2))))
  boxplot(data ~ source,
          data = xy, xlab = "", ylab = "")

  # ecdf
  ecdf_1 <- ecdf(sample_1)
  ecdf_2 <- ecdf(sample_2)
```

```
  xlim_low  <- min(sample_1,sample_2, na.rm = TRUE)
  xlim_high <- max(sample_1,sample_2, na.rm = TRUE)
  plot(ecdf_1, verticals=TRUE, do.points=FALSE, col = "blue",
       main = "Empirical Density Functions", ylab = "edf(x)",
       xlim = c(xlim_low,xlim_high))
  plot(ecdf_2, verticals=TRUE, do.points=FALSE, add=TRUE, col='brown')
  legend(x = "topleft",
         legend = c(colnames(name_1[1,1]),
                    colnames(name_2[1,1])),
         text.col  = c("blue","brown"),
         lty = 1, col = c("blue","brown"),lwd = 2)
  dev.off()

}
```

1.2 Interpreting the Plots

1.2.1 Box Whisker Plots

Box Whisker plot are well established in the description of data from experiments:

- the bold line marks the *median* value (this is the 50% quantile or second quartile Q_2)
- the box shows
  - the 25% quantile or first quartile (lower line) Q_1 and
  - the 75% quantile or third quartile (upper line) Q_3
- the lower whisker ends either in minimum or $Q_1 - 1.5 \times (Q_3 - Q_1)$ whichever is higher.
  - in case that the minimum of the data is lower than $Q_1 - 1.5 \times (Q_3 - Q_1)$, all data in that range are regarded as *suspicious to be outliers*
- the upper whisker ends either in maximum or $Q_3 + 1.5 \times (Q_3 - Q_1)$ whichever is lower.
  - in case that the maximum of the data is higher than $Q_3 + 1.5 \times (Q_3 - Q_1)$, all data in that range are regarded as *suspicious to be outliers*

Looking at the Box Whisker plot of one sample, one might get an impression about symmetry of the data set (with respect to the median) and of existence of suspicious data with respect of outliers.

Comparing two Box Whisker plots one may infer about differences of medians at least in a qualitive manner.

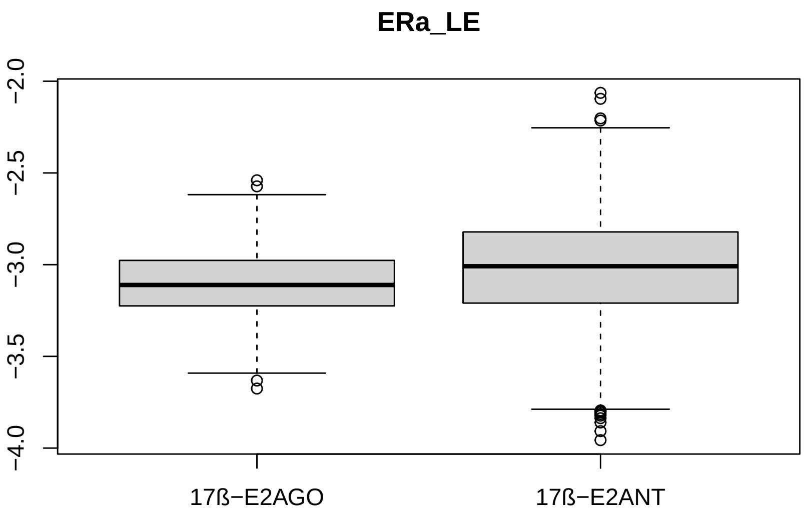
ERa_LE
−2.0
−2.5
−3.0
−3.5
−4.0
17ß−E2AGO
17ß−E2ANT

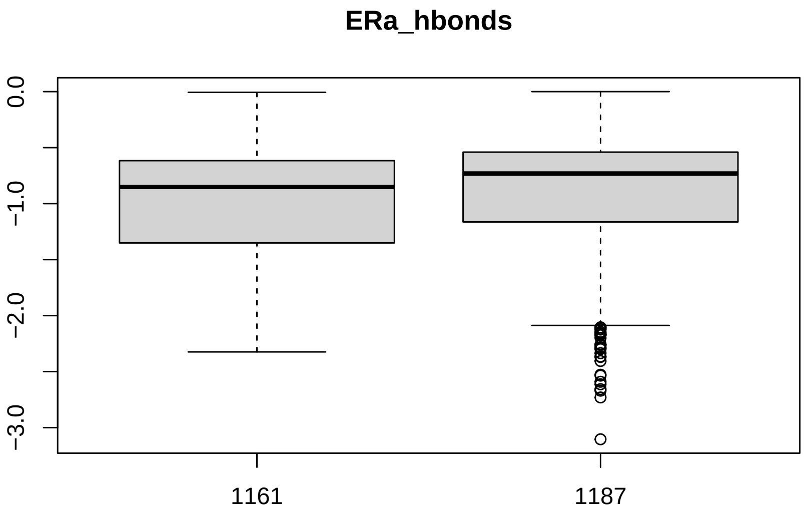
ERa_hbonds
0.0
−1.0
−2.0
−3.0
1161
1187

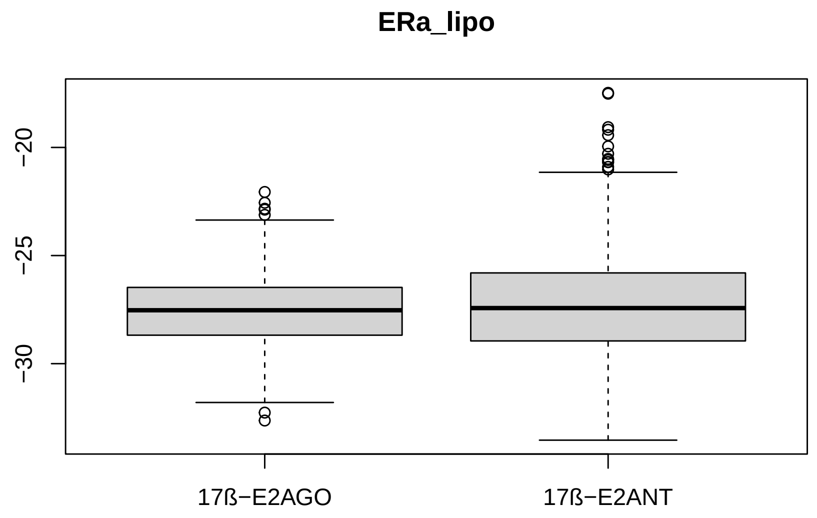
ERa_lipo
−20
−25
−30
17ß−E2AGO
17ß−E2ANT

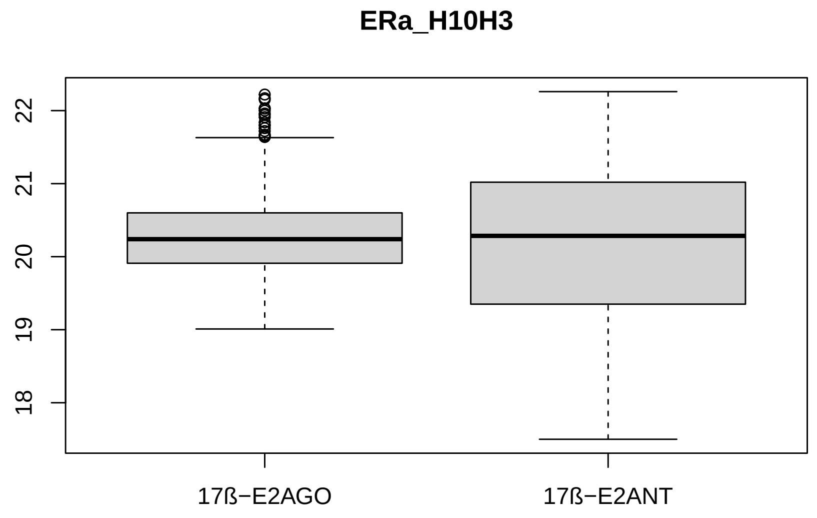
ERa_H10H3
22
21
20
19
18
17ß−E2AGO
17ß−E2ANT

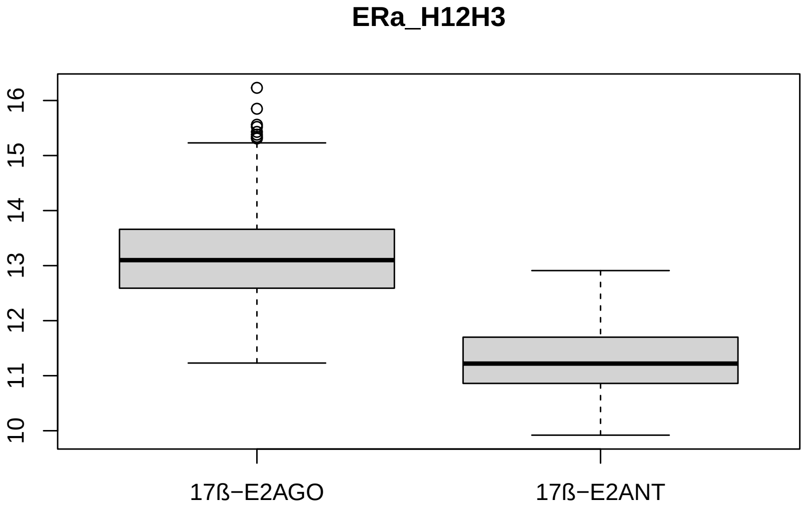
ERa_H12H3
16
15
14
13
12
11
10
17ß−E2AGO
17ß−E2ANT

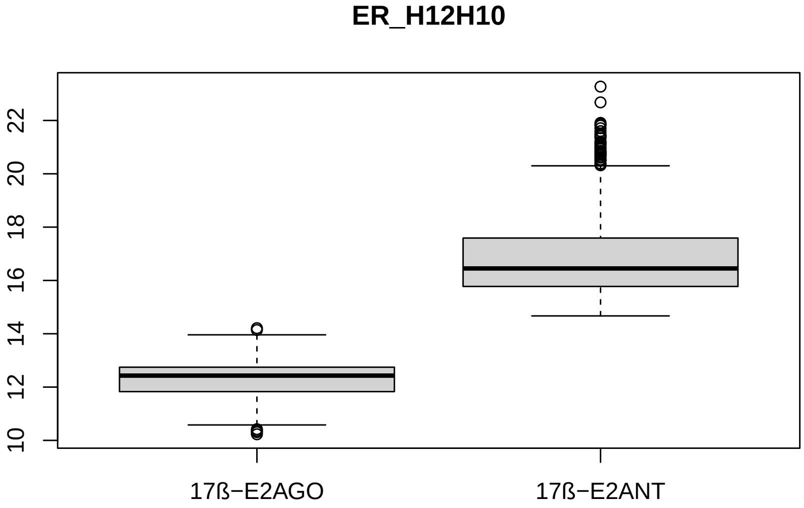
ER_H12H10
22
20
18
16
14
12
10
17ß−E2AGO
17ß−E2ANT

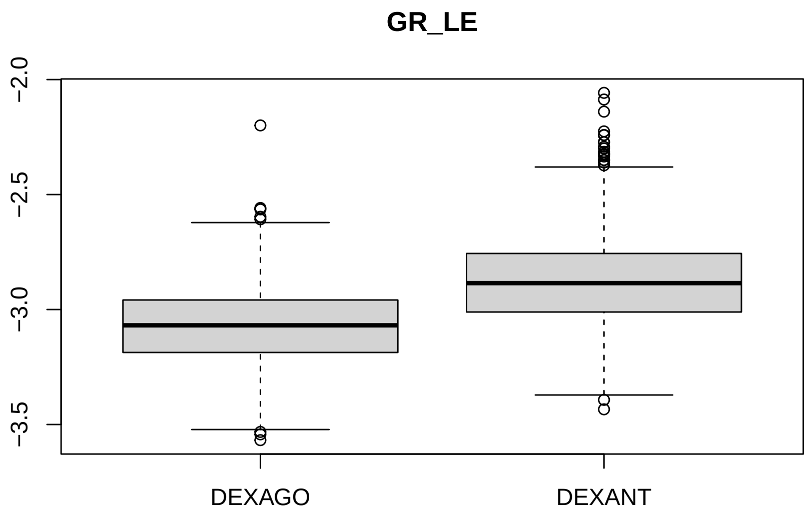
GR_LE
−2.0
−2.5
−3.0
−3.5
DEXAGO
DEXANT

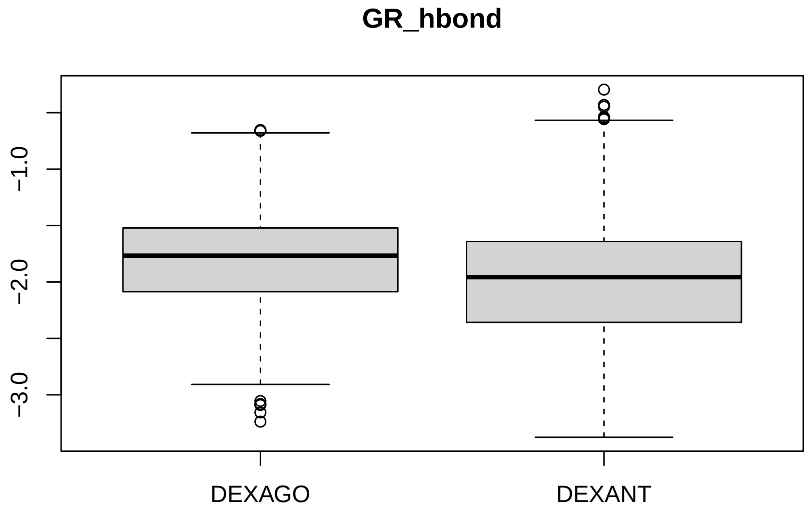
GR_hbond
−1.0
−2.0
−3.0
DEXAGO
DEXANT

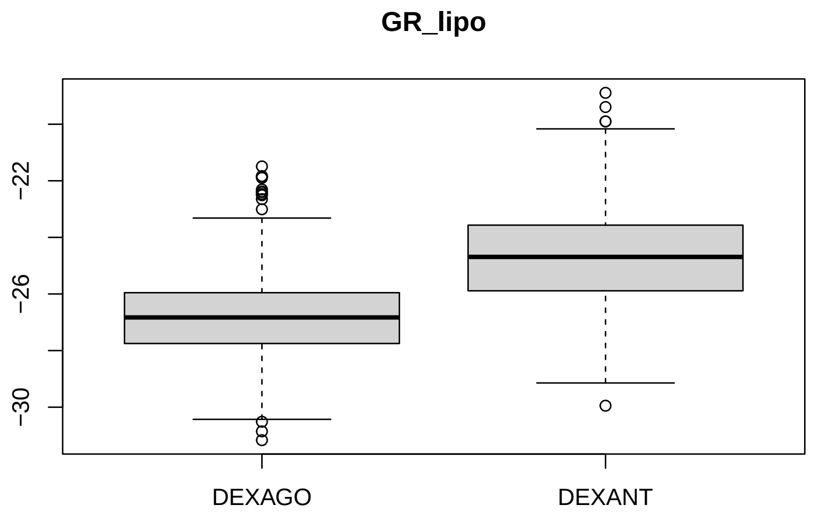
GR_lipo
−22
−26
−30
DEXAGO
DEXANT

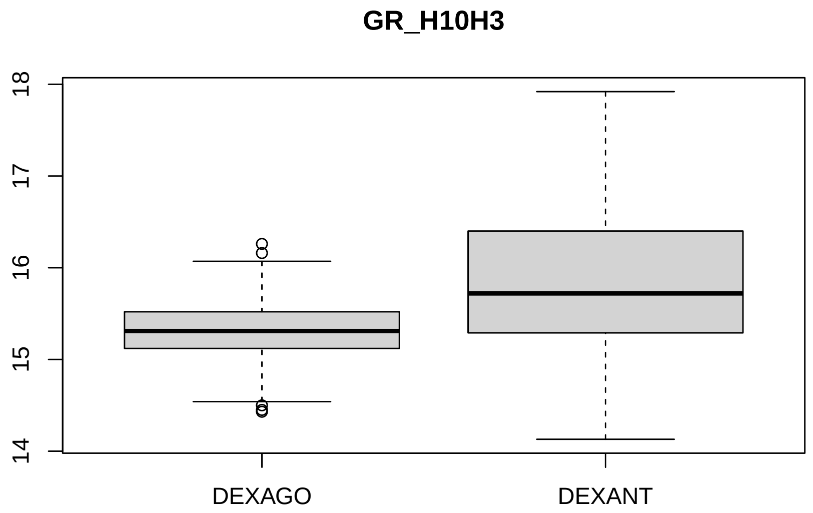
GR_H10H3
18
17
16
15
14
DEXAGO
DEXANT

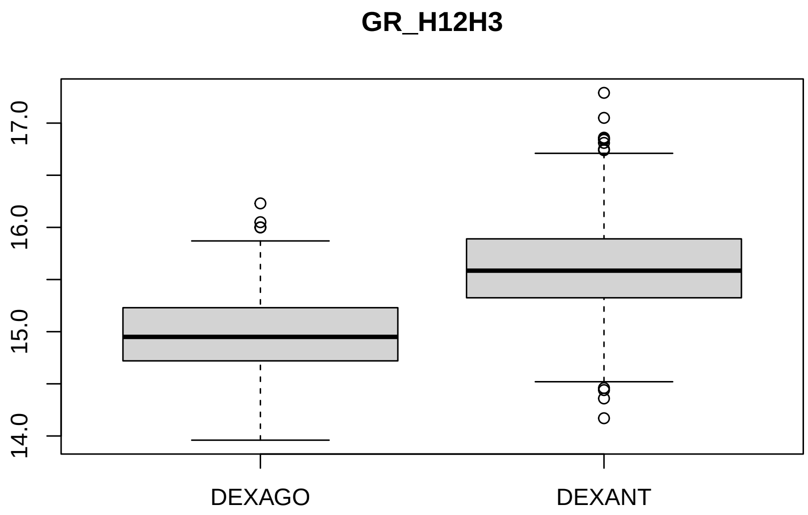

1.2.2 Empirical Cumulative Distribution Plots

Let $(x_n)_{n=1}^{N}$ be a sample measured on a continuous scale. The empirical (cumulative) distribution function is then defined by

$$ecfd(\xi) := \frac{\#\{n : x_n \leq \xi\}}{N} \tag{1.1}$$

of any real number ξ. That is one counts how many data points are less than ξ and divides the result by the number of items in the sample. Obviously the empirical distribution function is increasing and takes values in the range between 0 and 1 (both included).

We have the relations

- $ecdf(Q_1) = 0.25$
- $ecdf(Q_2) = 0.50$
- $ecdf(Q_3) = 0.75$

So we can identify the quartiles and other quantiles in ecdf plots. The general rule is: The flatter the ecdf the larger the variance.

Looking at the ecdf of one sample, we may get an impression about symmetry (again with respect to the median) and information about the general shape. When two ecdfs are compared, a parallel shift in curves indicates a difference in median and/or mean. If the curves cross at about the median, the variances may be different.

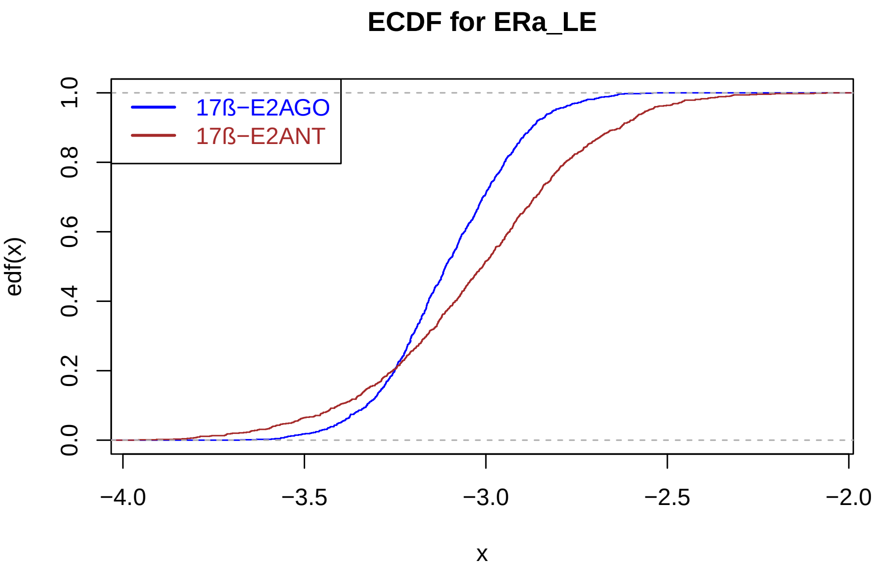
ECDF for ERa_LE
17ß−E2AGO
17ß−E2ANT
edf(x)
0.0
0.2
0.4
0.6
0.8
1.0
−4.0
−3.5
−3.0
−2.5
−2.0
x

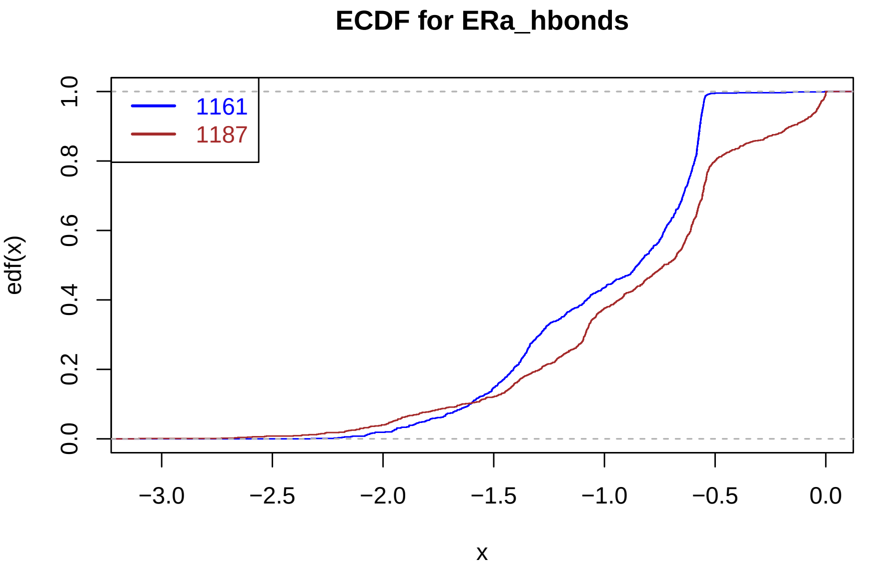
ECDF for ERa_hbonds
1161
1187
edf(x)
0.0
0.2
0.4
0.6
0.8
1.0
−3.0
−2.5
−2.0
−1.5
−1.0
−0.5
0.0
x

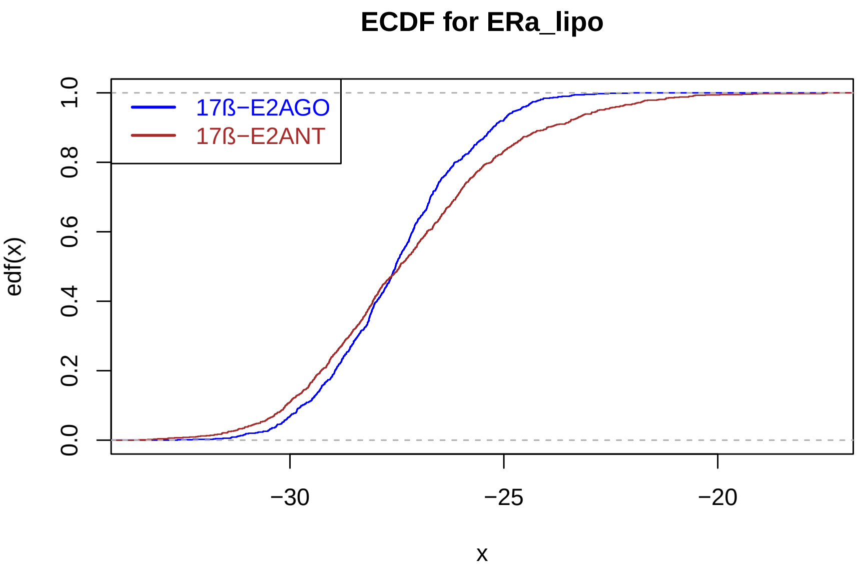
ECDF for ERa_lipo
17ß−E2AGO
17ß−E2ANT
edf(x)
0.0
0.2
0.4
0.6
0.8
1.0
−30
−25
−20
x

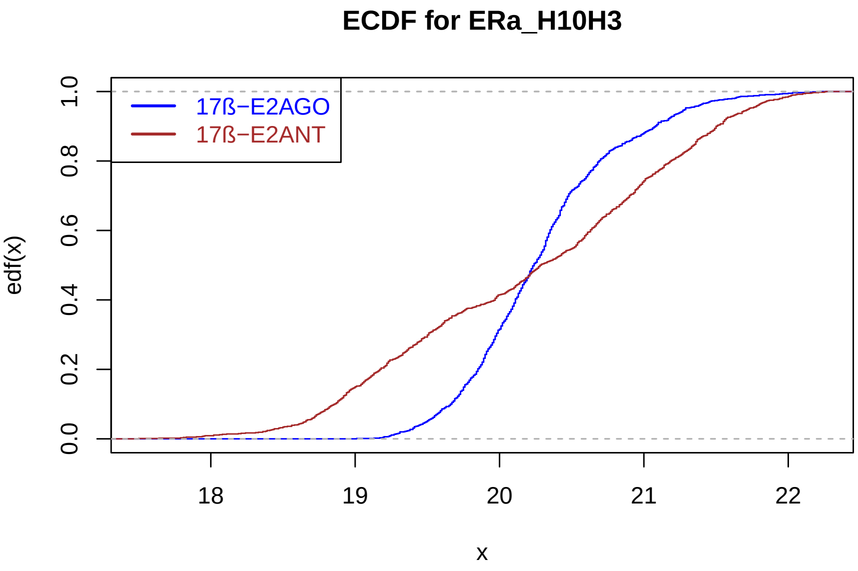
ECDF for ERa_H10H3
17ß−E2AGO
17ß−E2ANT
edf(x)
0.0
0.2
0.4
0.6
0.8
1.0
18
19
20
21
22
x

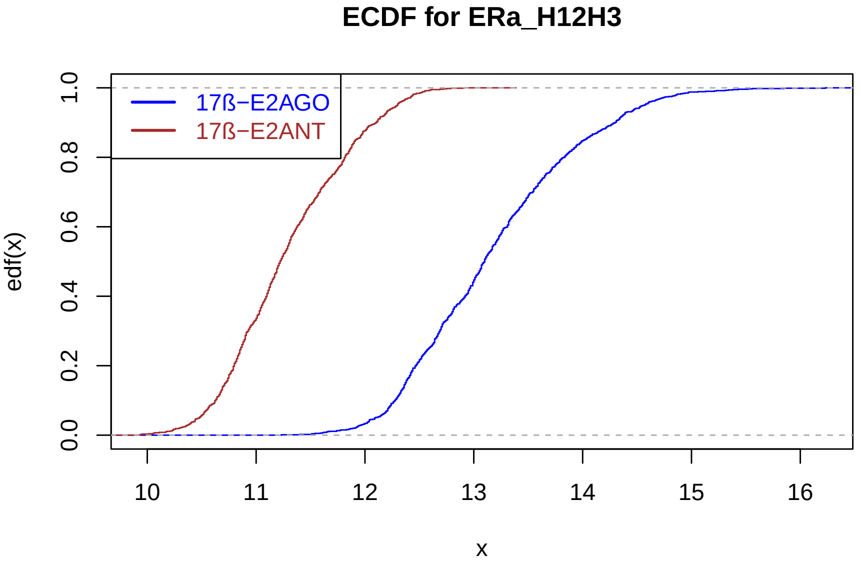
ECDF for ERa_H12H3
17ß−E2AGO
17ß−E2ANT
edf(x)
0.0
0.2
0.4
0.6
0.8
1.0
10
11
12
13
14
15
16
x

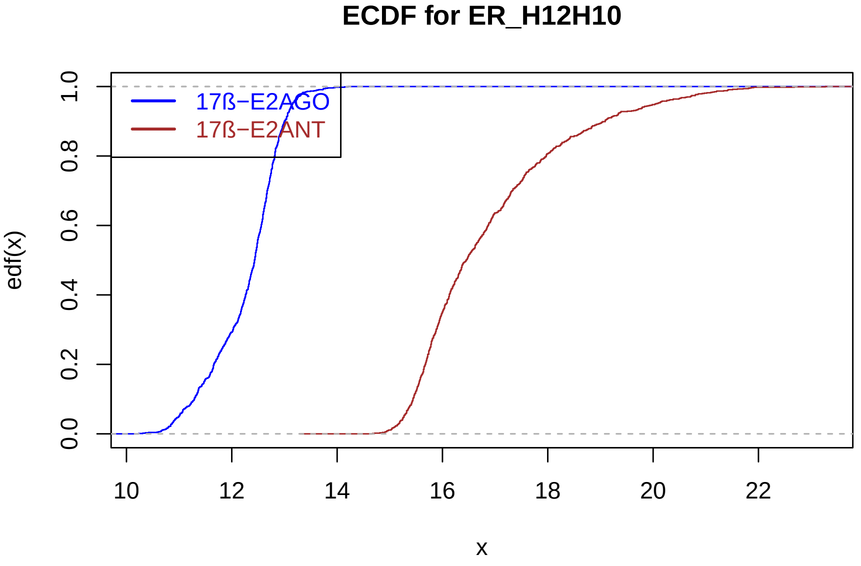
ECDF for ER_H12H10
17ß−E2AGO
17ß−E2ANT
edf(x)
0.0
0.2
0.4
0.6
0.8
1.0
10
12
14
16
18
20
22
x

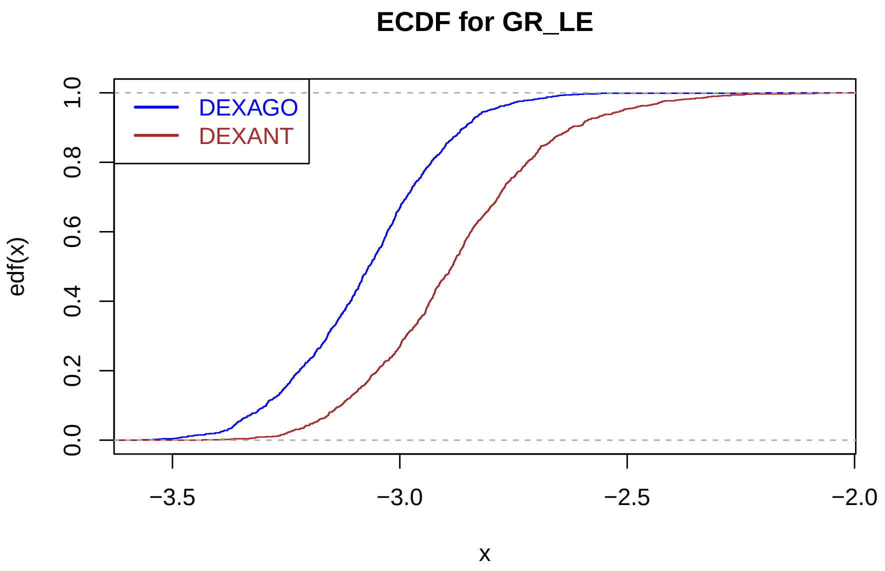
ECDF for GR_LE
DEXAGO
DEXANT
edf(x)
0.0
0.2
0.4
0.6
0.8
1.0
−3.5
−3.0
−2.5
−2.0
x

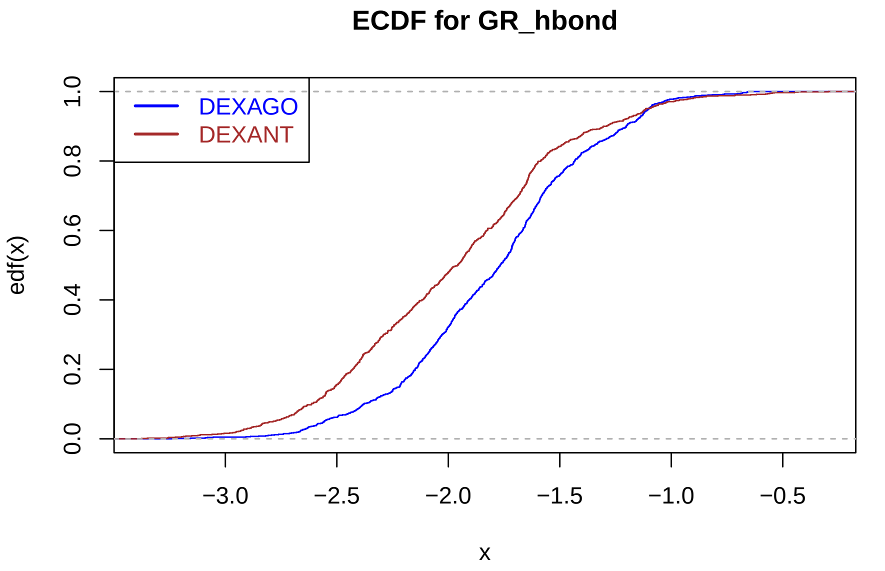
ECDF for GR_hbond
DEXAGO
DEXANT
edf(x)
0.0
0.2
0.4
0.6
0.8
1.0
−3.0
−2.5
−2.0
−1.5
−1.0
−0.5
x

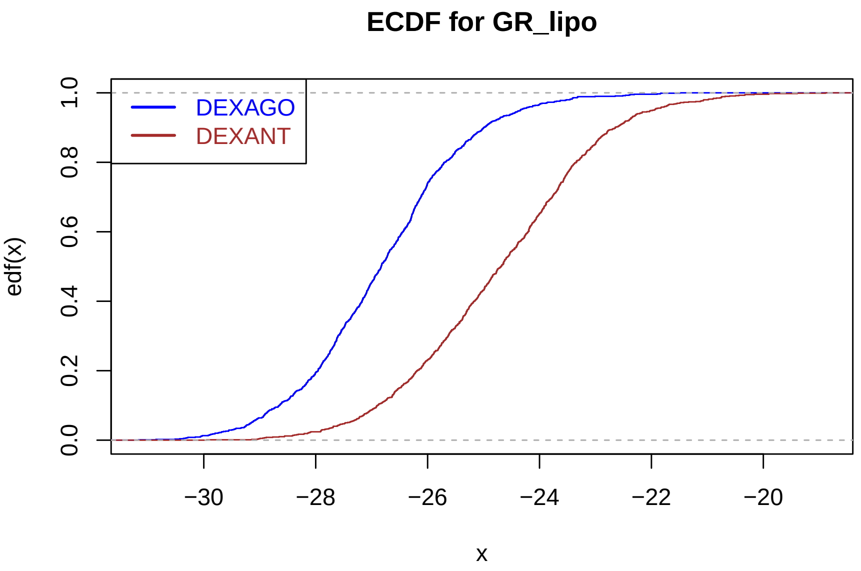
ECDF for GR_lipo
DEXAGO
DEXANT
edf(x)
0.0
0.2
0.4
0.6
0.8
1.0
−30
−28
−26
−24
−22
−20
x

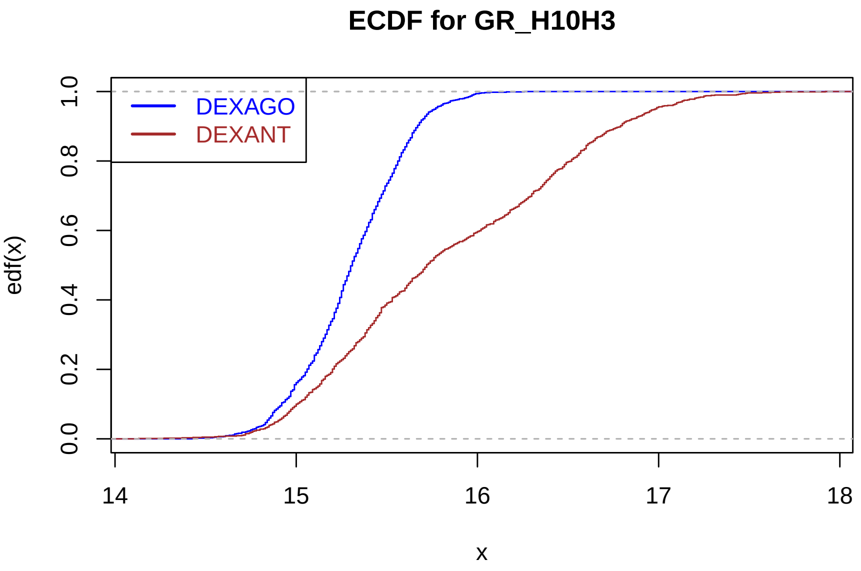
ECDF for GR_H10H3
DEXAGO
DEXANT
edf(x)
0.0
0.2
0.4
0.6
0.8
1.0
14
15
16
17
18
x

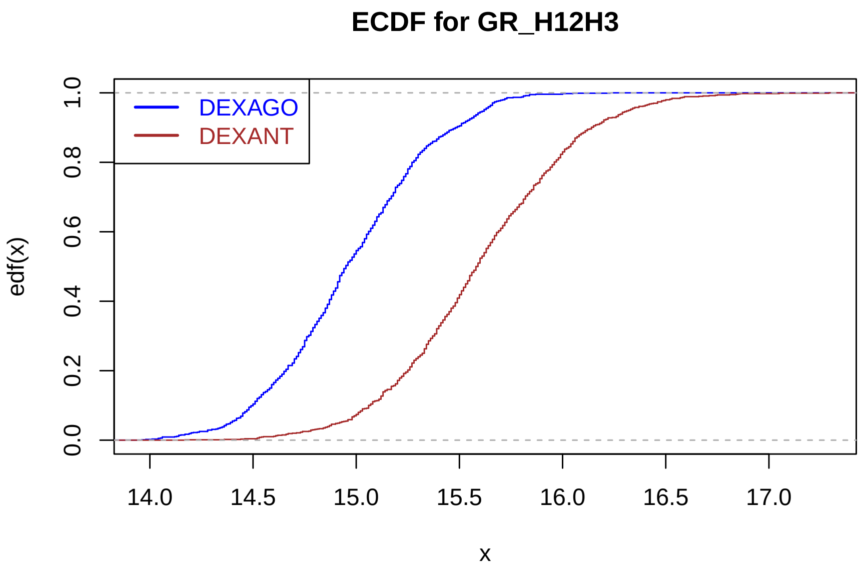

1.2.3 Quantile-Quantile Plots

A common way to check whether two samples are likely to follow the same distribution function is a quantile-quantile plot. Let $(x_m)_{m=1}^{M}$ and $(y_n)_{n=1}^{N}$ be two samples with the ecdfs $ecdf_X, ecdf_Y$ respectively. For some $0 < t < 1$ let ξ_X and ξ_X given by

$$\begin{aligned} ecds_X(\xi_X) &= t \\ ecds_Y(\xi_Y) &= t \end{aligned} \tag{1.2}$$

Then for a quantile-quantile plot ξ_Y is put against ξ_X into a diagram. If the two samples follow the same distributions, these plots are near to the diagonal.

If the quantile-quantile plot is parallel to the diagonal, this hints to the distributions being distinct but of the same shape with presumably different means or medians.

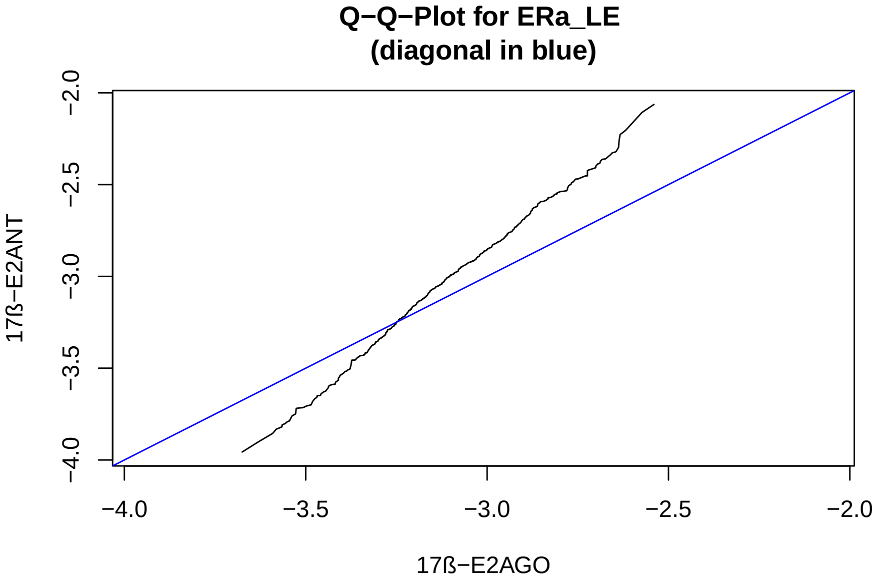
Q−Q−Plot for ERa_LE
(diagonal in blue)
−2.0
−2.5
−3.0
−3.5
−4.0
17ß−E2ANT
−4.0
−3.5
−3.0
−2.5
−2.0
17ß−E2AGO

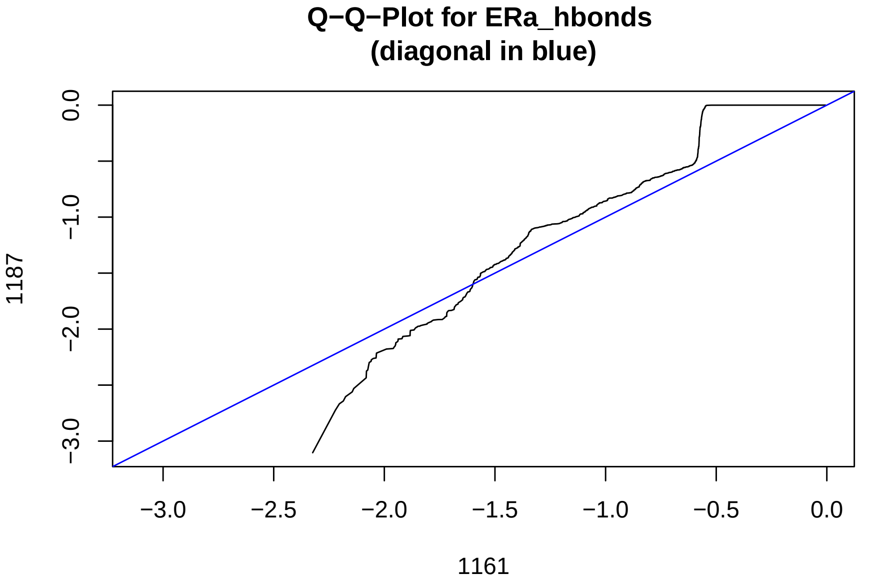
Q−Q−Plot for ERa_hbonds
(diagonal in blue)
0.0
−1.0
−2.0
−3.0
1187
−3.0
−2.5
−2.0
−1.5
−1.0
−0.5
0.0
1161

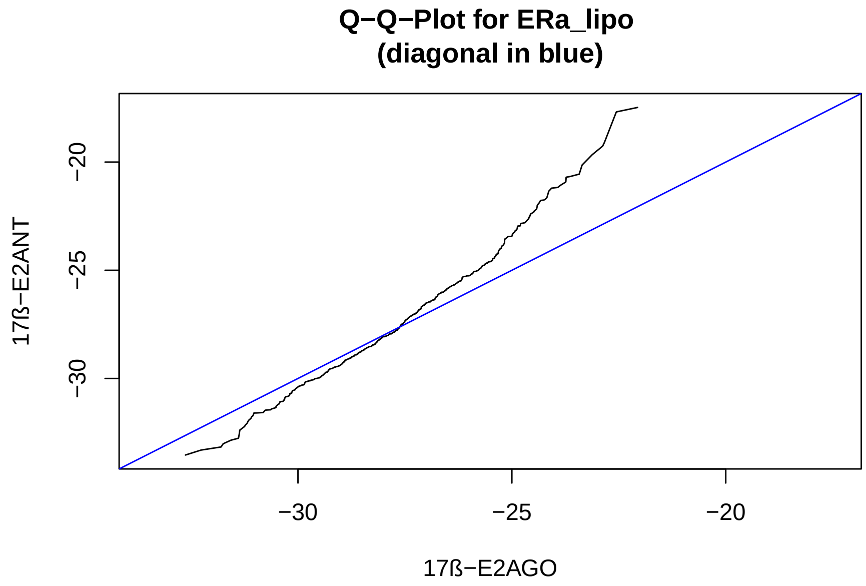
Q−Q−Plot for ERa_lipo
(diagonal in blue)
17ß−E2ANT
−20
−25
−30
−30
−25
−20
17ß−E2AGO

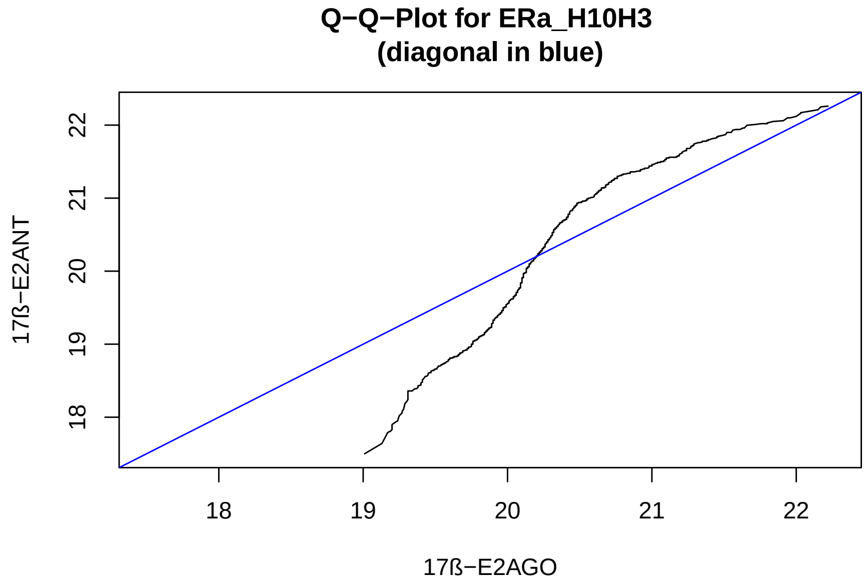
Q−Q−Plot for ERa_H10H3
(diagonal in blue)
17ß−E2ANT
22
21
20
19
18
18
19
20
21
22
17ß−E2AGO

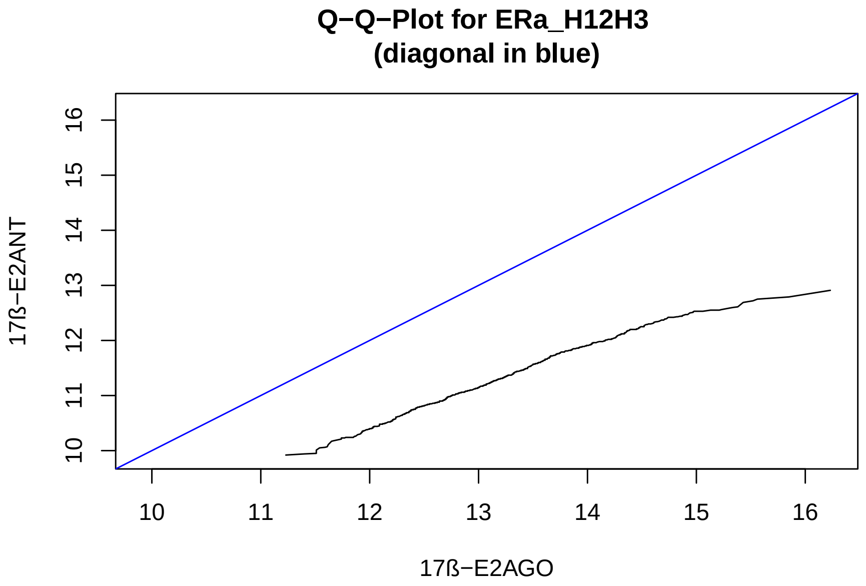
Q−Q−Plot for ERa_H12H3
(diagonal in blue)
17ß−E2ANT
17ß−E2AGO
10
11
12
13
14
15
16

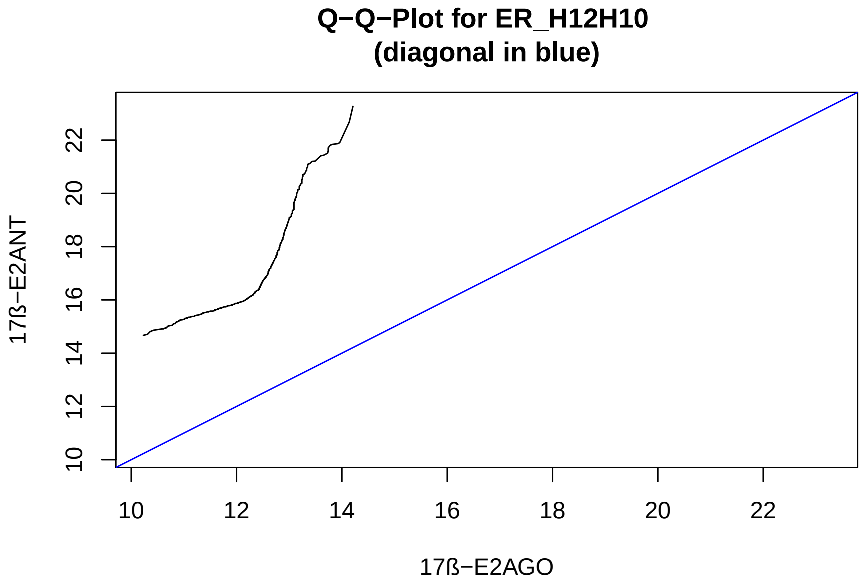
Q−Q−Plot for ER_H12H10
(diagonal in blue)
17ß−E2ANT
17ß−E2AGO
10
12
14
16
18
20
22

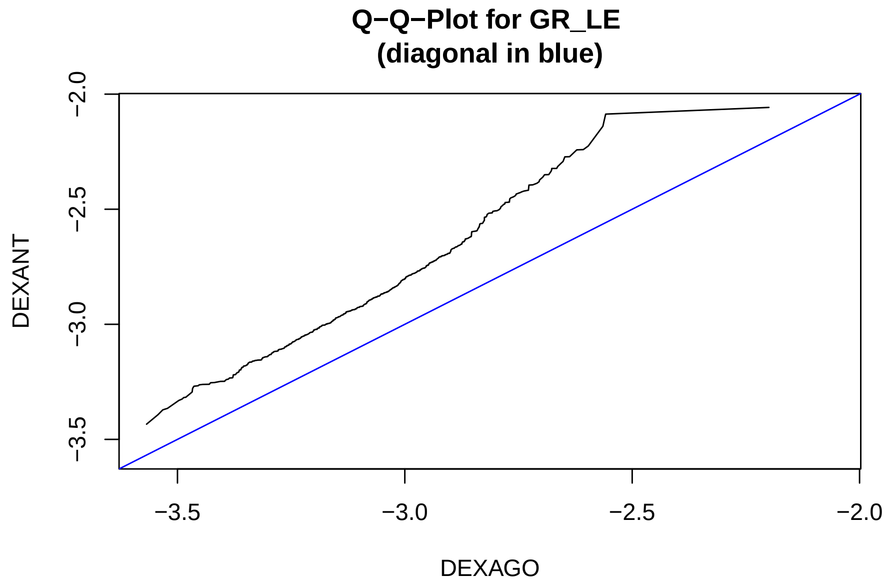
Q−Q−Plot for GR_LE
(diagonal in blue)
DEXANT
DEXAGO
−2.0
−2.5
−3.0
−3.5
−3.5
−3.0
−2.5
−2.0

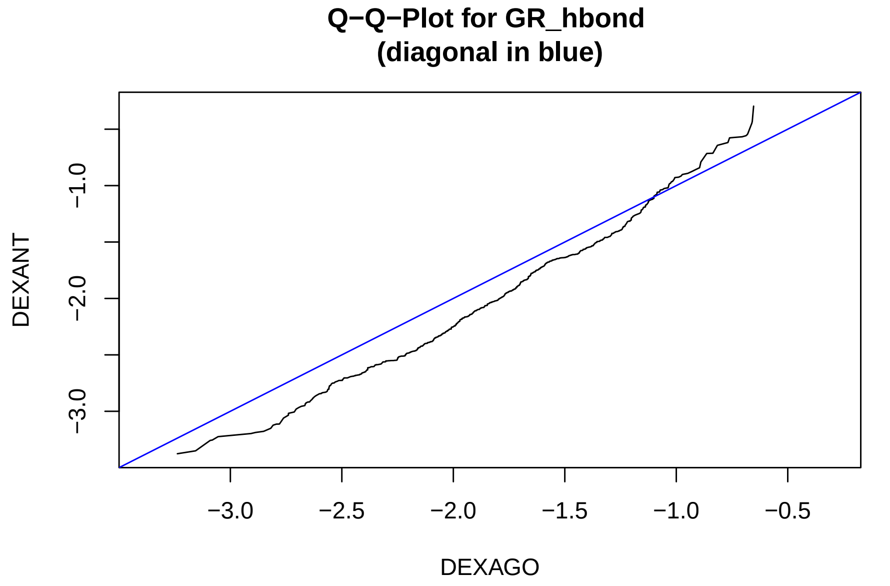
Q−Q−Plot for GR_hbond
(diagonal in blue)
DEXANT
DEXAGO
−1.0
−2.0
−3.0
−3.0
−2.5
−2.0
−1.5
−1.0
−0.5

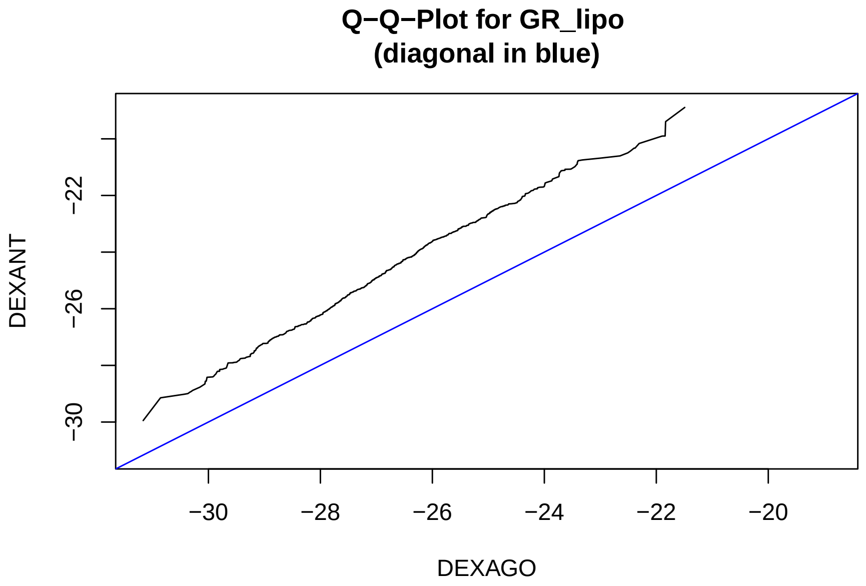
Q−Q−Plot for GR_lipo
(diagonal in blue)
DEXANT
−22
−26
−30
−30
−28
−26
−24
−22
−20
DEXAGO

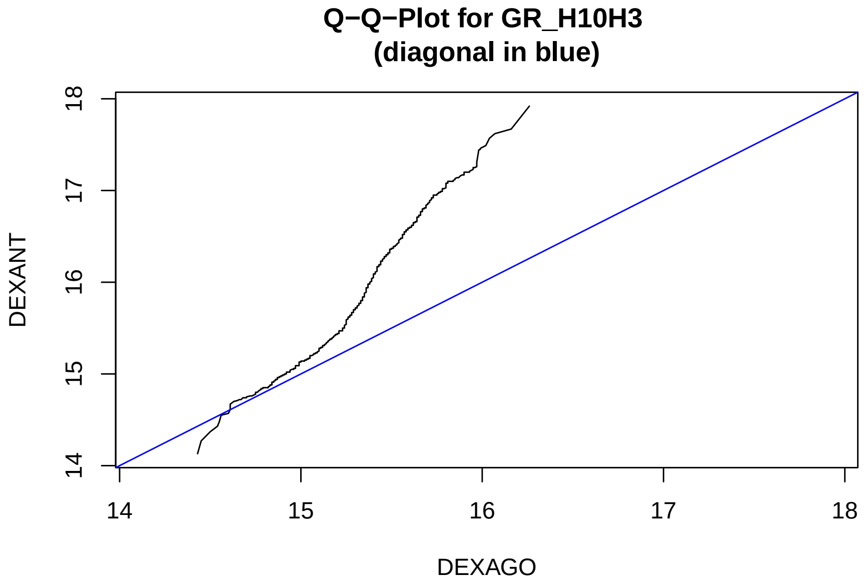
Q−Q−Plot for GR_H10H3
(diagonal in blue)
DEXANT
18
17
16
15
14
14
15
16
17
18
DEXAGO

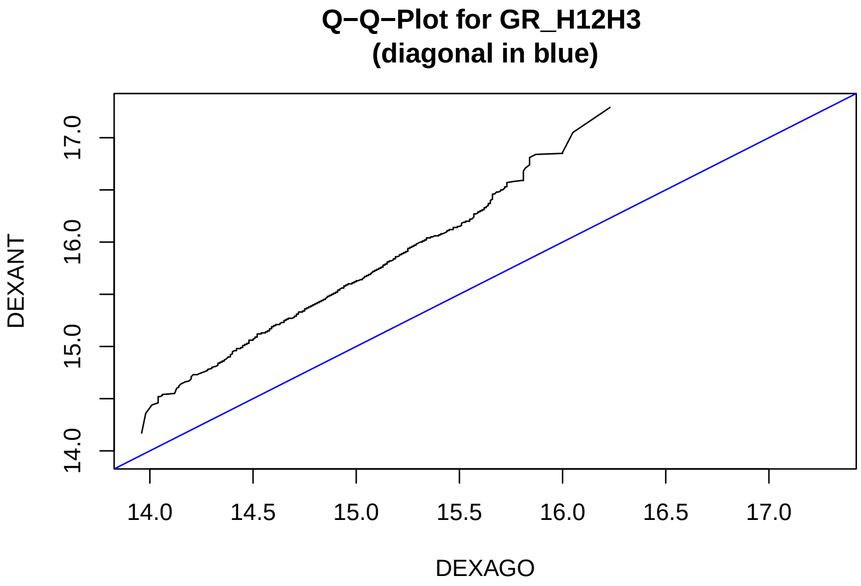
Q−Q−Plot for GR_H12H3
(diagonal in blue)
DEXANT
14.0
15.0
16.0
17.0
14.0
14.5
15.0
15.5
16.0
16.5
17.0
DEXAGO

Bibliography

JJ Allaire, Yihui Xie, Christophe Dervieux, Jonathan McPherson, Javier Luraschi, Kevin Ushey, Aron Atkins, Hadley Wickham, Joe Cheng, Winston Chang, and Richard Iannone. *rmarkdown: Dynamic Documents for R*, 2023. URL https://github.com/rstudio/rmarkdown. R package version 2.25.

R Core Team. *R: A Language and Environment for Statistical Computing*. R Foundation for Statistical Computing, Vienna, Austria, 2023. URL https://www.R-project.org/.

Hadley Wickham and Jennifer Bryan. *readxl: Read Excel Files*, 2023. URL https://readxl.tidyverse.org. R package version 1.4.3.

Yihui Xie. knitr: A comprehensive tool for reproducible research in R. In Victoria Stodden, Friedrich Leisch, and Roger D. Peng, editors, *Implementing Reproducible Computational Research*. Chapman and Hall/CRC, 2014. ISBN 978-1466561595.

Yihui Xie. *Dynamic Documents with R and knitr*. Chapman and Hall/CRC, Boca Raton, Florida, 2nd edition, 2015. URL https://yihui.org/knitr/. ISBN 978-1498716963.

Yihui Xie. *bookdown: Authoring Books and Technical Documents with R Markdown*. Chapman and Hall/CRC, Boca Raton, Florida, 2016. ISBN 978-1138700109. URL https://bookdown.org/yihui/bookdown.

Yihui Xie. *bookdown: Authoring Books and Technical Documents with R Markdown*, 2023a. URL https://github.com/rstudio/bookdown. R package version 0.35.

Yihui Xie. *knitr: A General-Purpose Package for Dynamic Report Generation in R*, 2023b. URL https://yihui.org/knitr/. R package version 1.44.

Yihui Xie, J.J. Allaire, and Garrett Grolemund. *R Markdown: The Definitive Guide*. Chapman and Hall/CRC, Boca Raton, Florida, 2018. ISBN 9781138359338. URL https://bookdown.org/yihui/rmarkdown.

Yihui Xie, Christophe Dervieux, and Emily Riederer. *R Markdown Cookbook*.

Chapman and Hall/CRC, Boca Raton, Florida, 2020. ISBN 9780367563837. URL https://bookdown.org/yihui/rmarkdown-cookbook.
