# Supplementary figures and images for: Look What You Made Me Do: Discerning Feature for Classification of Endocrine-Disrupting Chemical Binding to Steroid Hormone Receptors
Source: J Chem Inf Model. 2025 Apr 9;65(8):4148–62. doi: 10.1021/acs.jcim.4c02288 (PMC12042260; doi:10.1021/acs.jcim.4c02288)

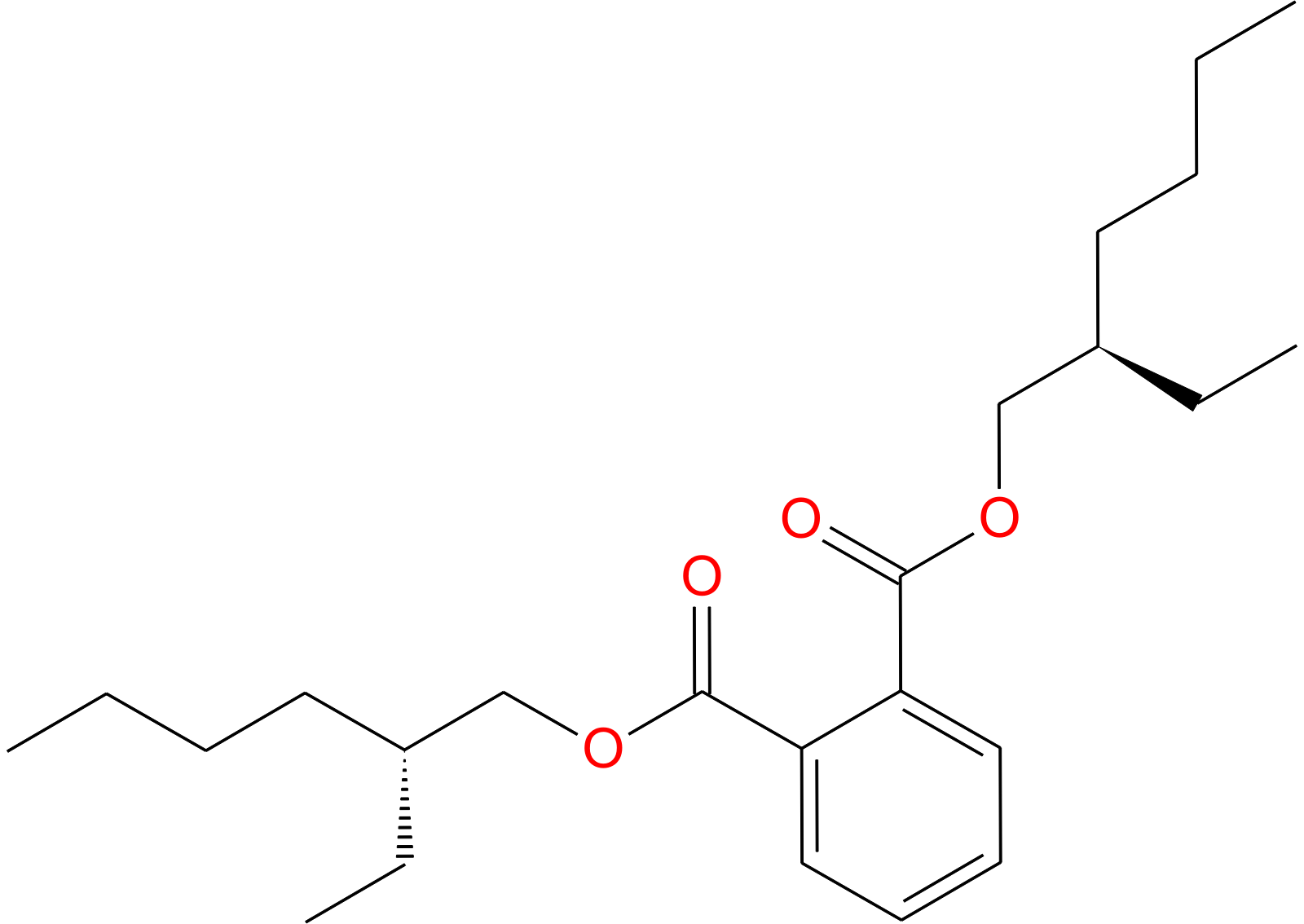

Supplement: Supplementary file 2 — ci4c02288_si_002.zip [file ci4c02288_si_002.zip › SI_v3_appendix/1162_RR_DEHP_ER/images/L_2d_main.png]

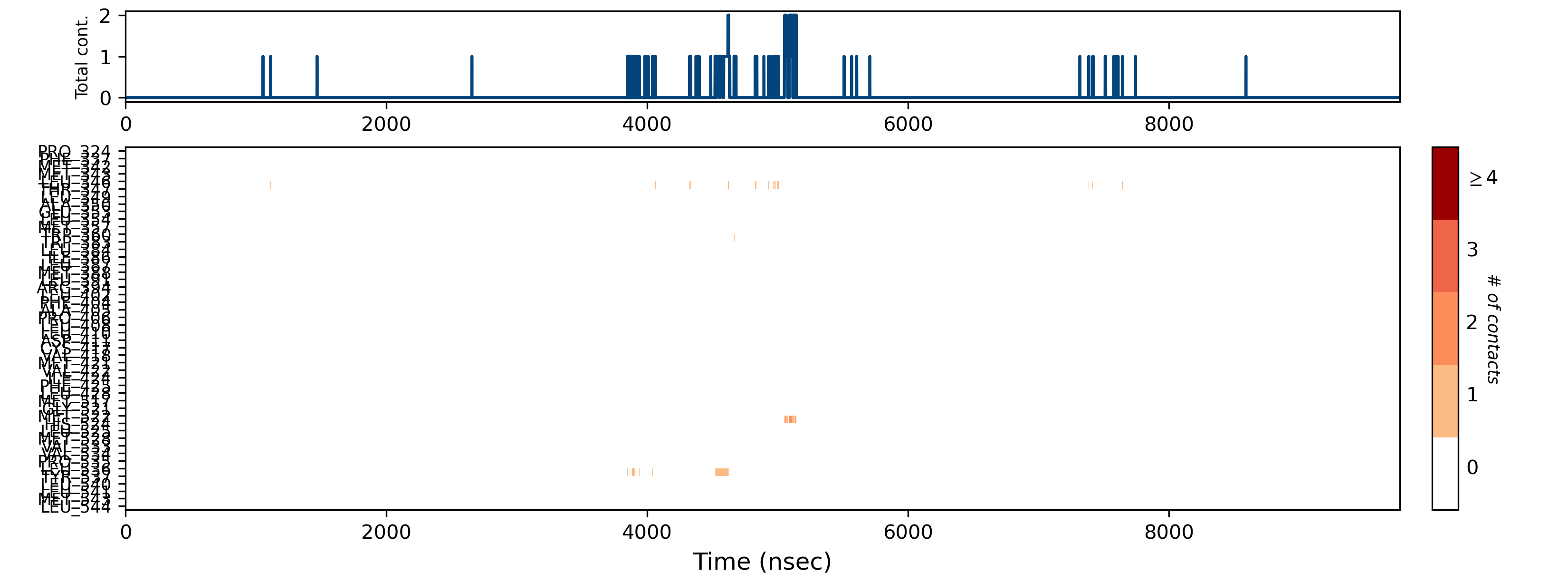

Supplement: Supplementary file 2 — ci4c02288_si_002.zip [file ci4c02288_si_002.zip › SI_v3_appendix/1162_RR_DEHP_ER/images/PL-Contacts_Timeline.png]

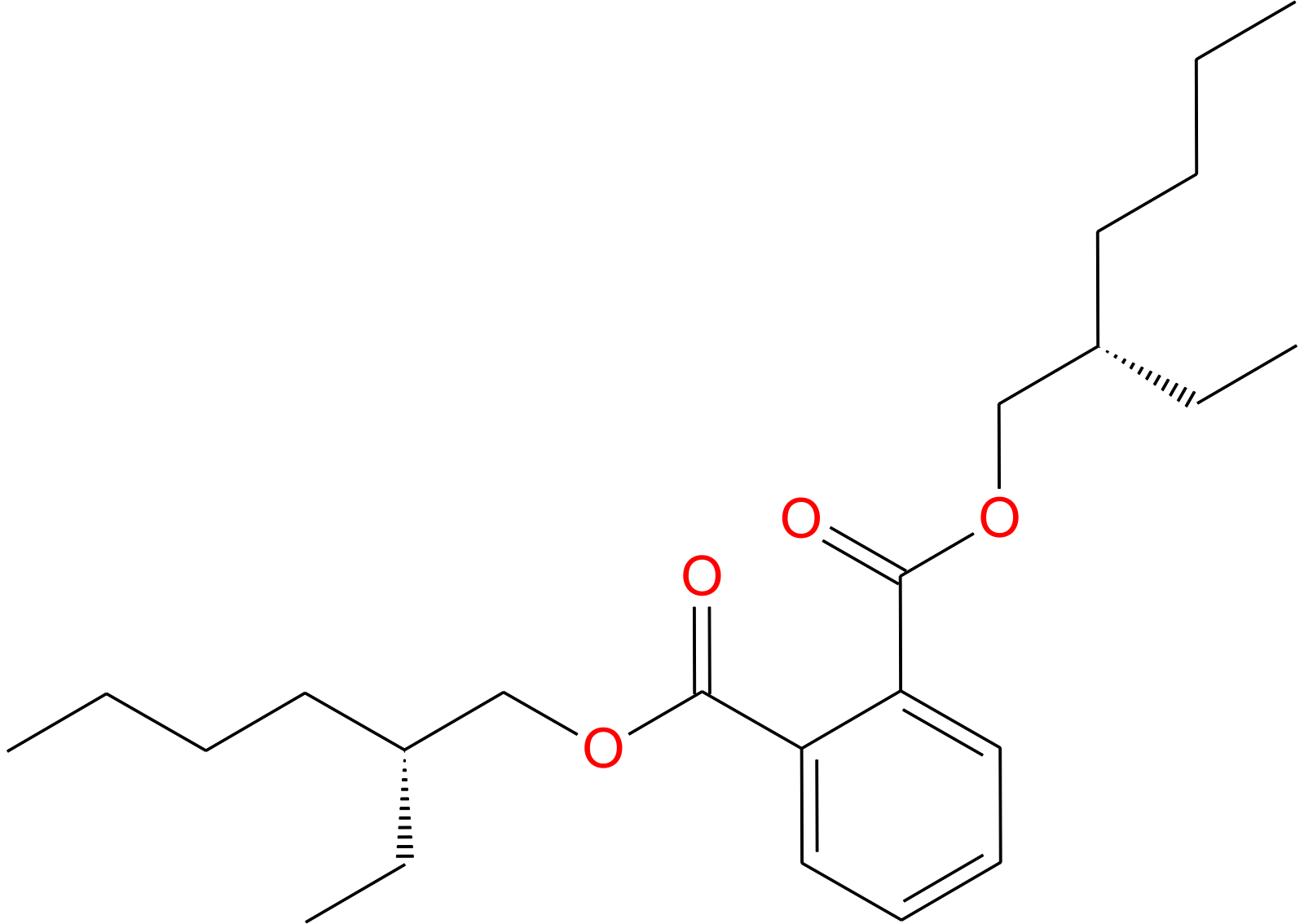

Supplement: Supplementary file 2 — ci4c02288_si_002.zip [file ci4c02288_si_002.zip › SI_v3_appendix/1163_RS_DEHP/images/L_2d_main.png]

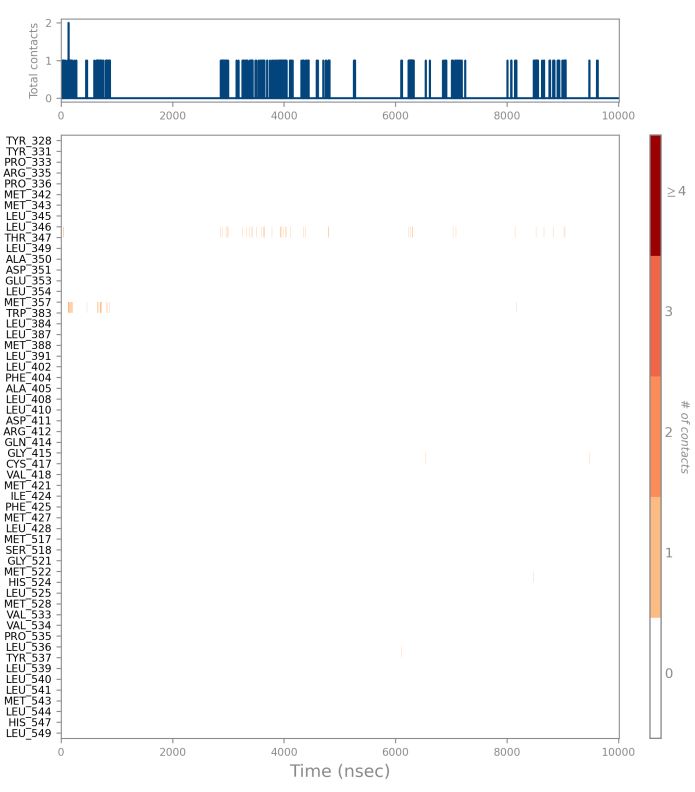

Supplement: Supplementary file 2 — ci4c02288_si_002.zip [file ci4c02288_si_002.zip › SI_v3_appendix/1163_RS_DEHP/images/PL_contact_Timeline.JPG]

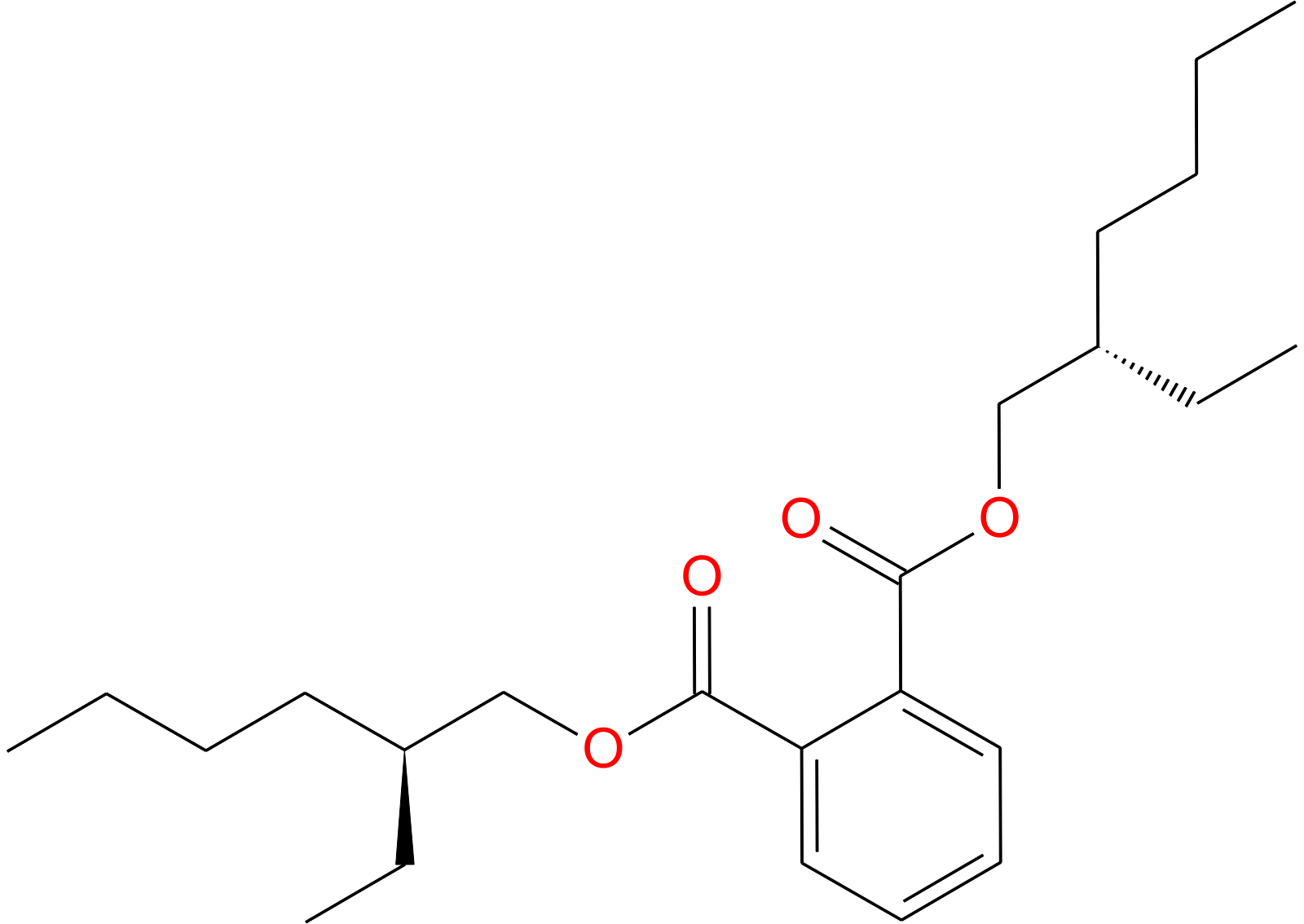

Supplement: Supplementary file 2 — ci4c02288_si_002.zip [file ci4c02288_si_002.zip › SI_v3_appendix/1164_SS_DEHP_ER/images/L_2d_main.png]

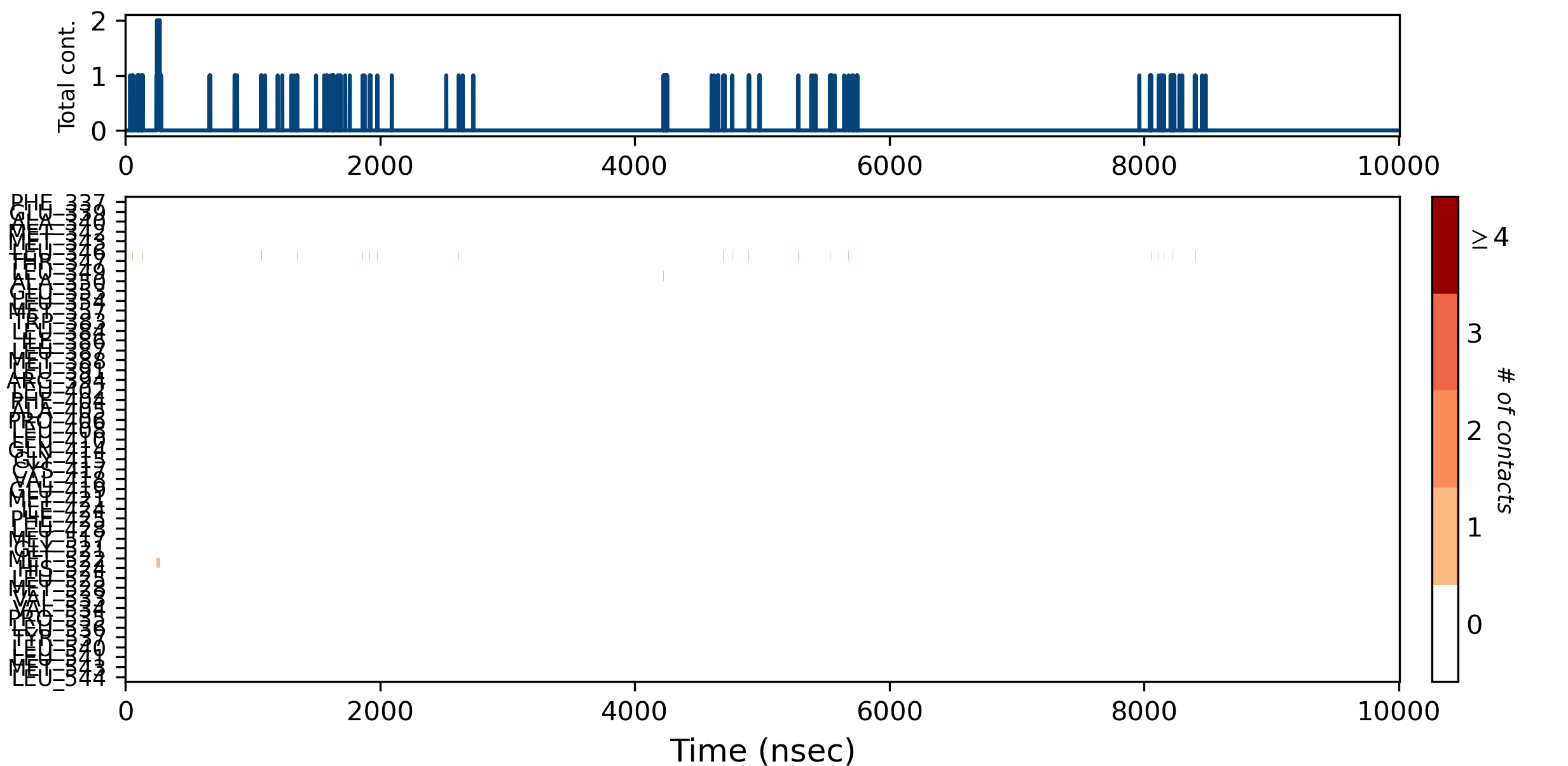

Supplement: Supplementary file 2 — ci4c02288_si_002.zip [file ci4c02288_si_002.zip › SI_v3_appendix/1164_SS_DEHP_ER/images/PL-Contacts_Timeline.png]

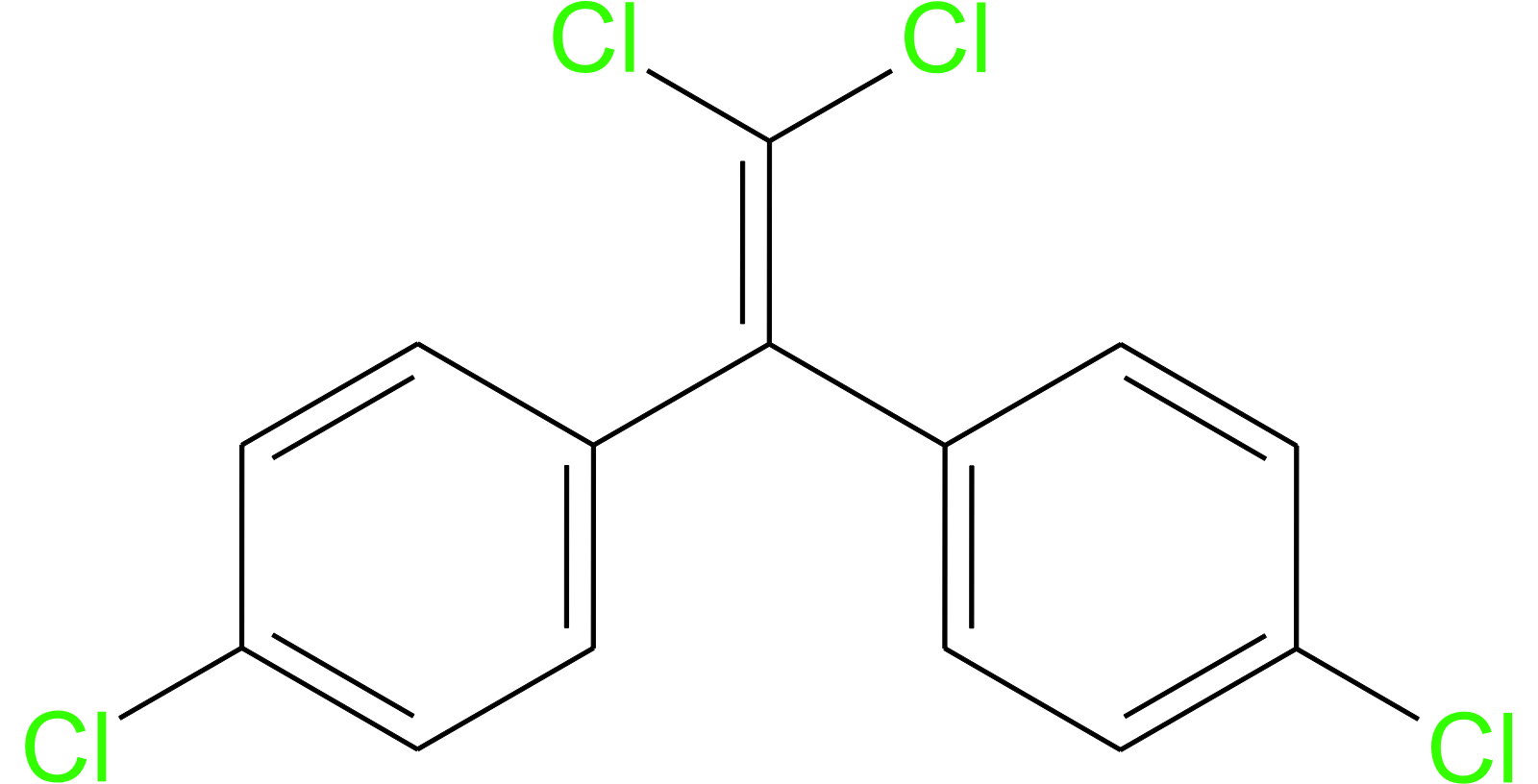

Supplement: Supplementary file 2 — ci4c02288_si_002.zip [file ci4c02288_si_002.zip › SI_v3_appendix/1167_DDE_ER/images/L_2d_main.png]

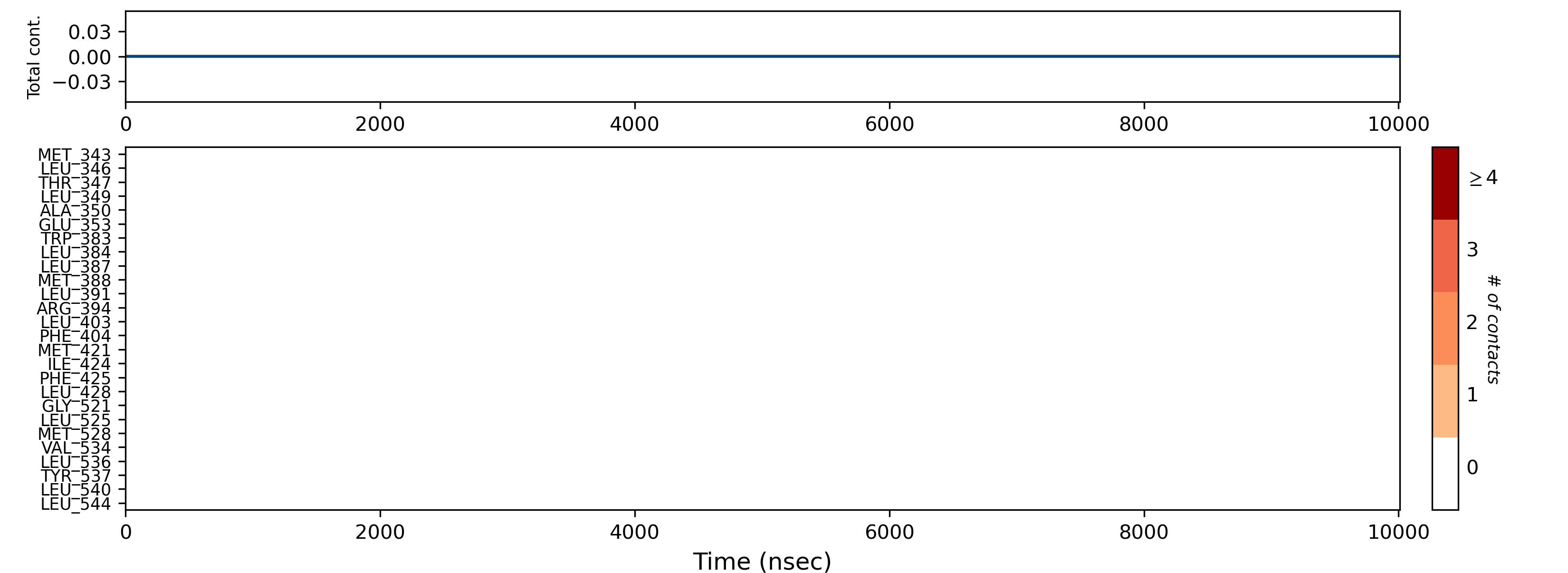

Supplement: Supplementary file 2 — ci4c02288_si_002.zip [file ci4c02288_si_002.zip › SI_v3_appendix/1167_DDE_ER/images/PL-Contacts_Timeline.png]

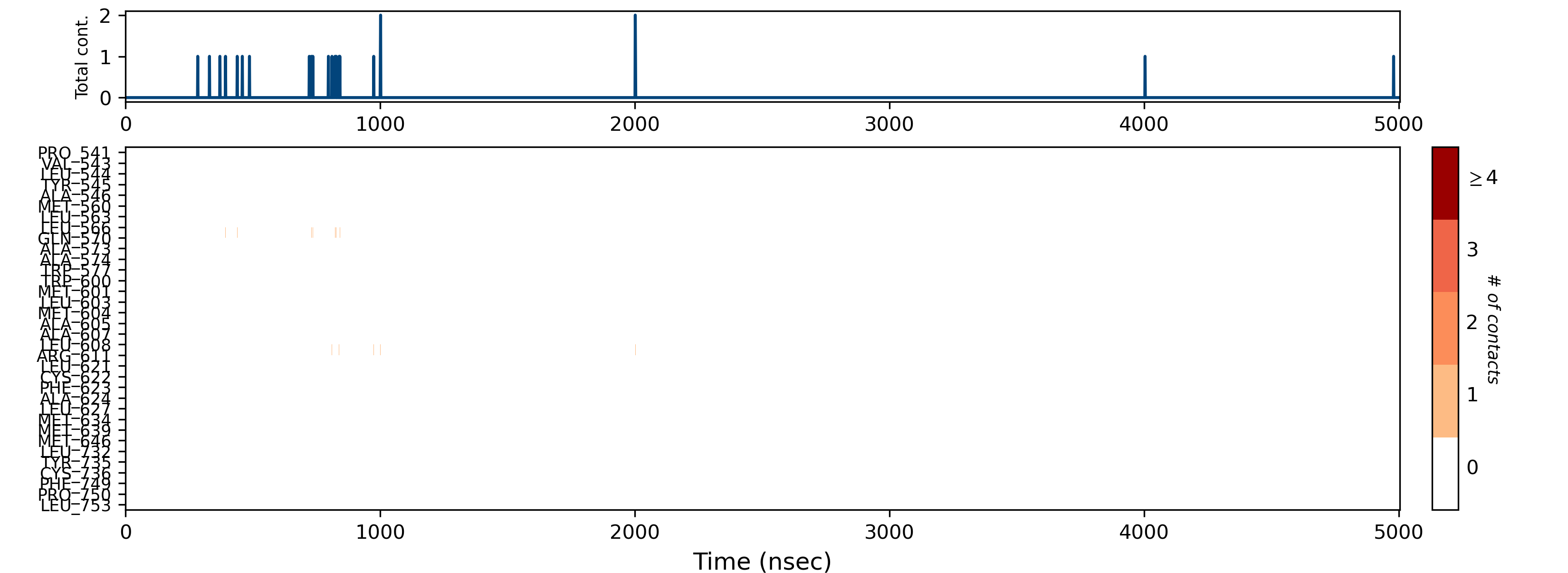

Supplement: Supplementary file 2 — ci4c02288_si_002.zip [file ci4c02288_si_002.zip › SI_v3_appendix/1170_RR_DEHP_GR/images/PL-Contacts_Timeline.png]

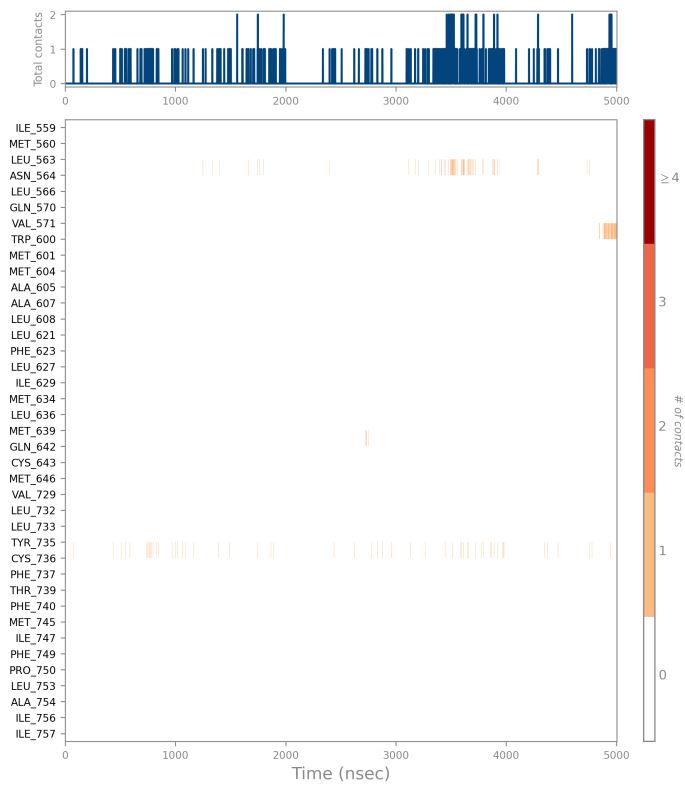

Supplement: Supplementary file 2 — ci4c02288_si_002.zip [file ci4c02288_si_002.zip › SI_v3_appendix/1171_RS_DEHP_GR/images/PL_contact_Timeline.JPG]

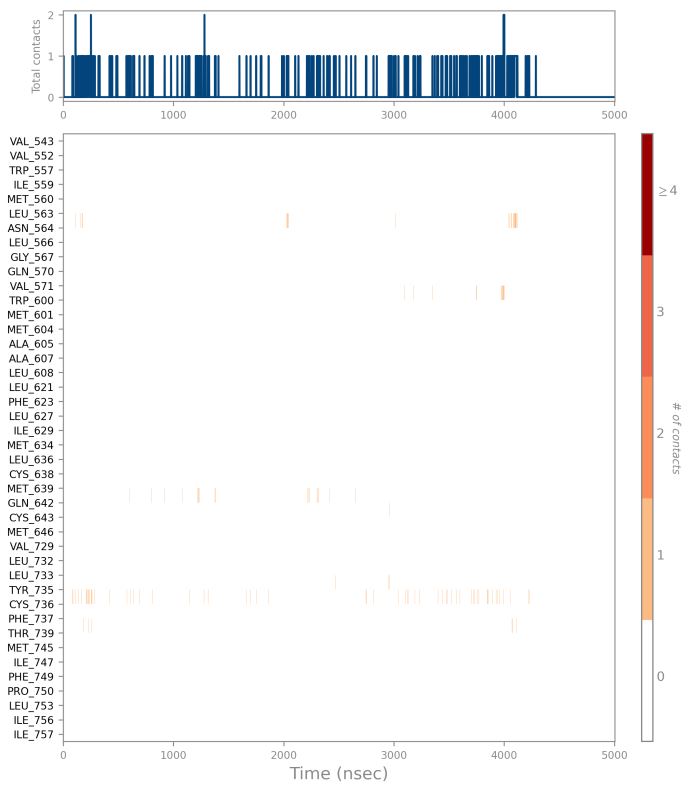

Supplement: Supplementary file 2 — ci4c02288_si_002.zip [file ci4c02288_si_002.zip › SI_v3_appendix/1172_SS_DEHP_GR/images/PL_contact_Timeline.JPG]

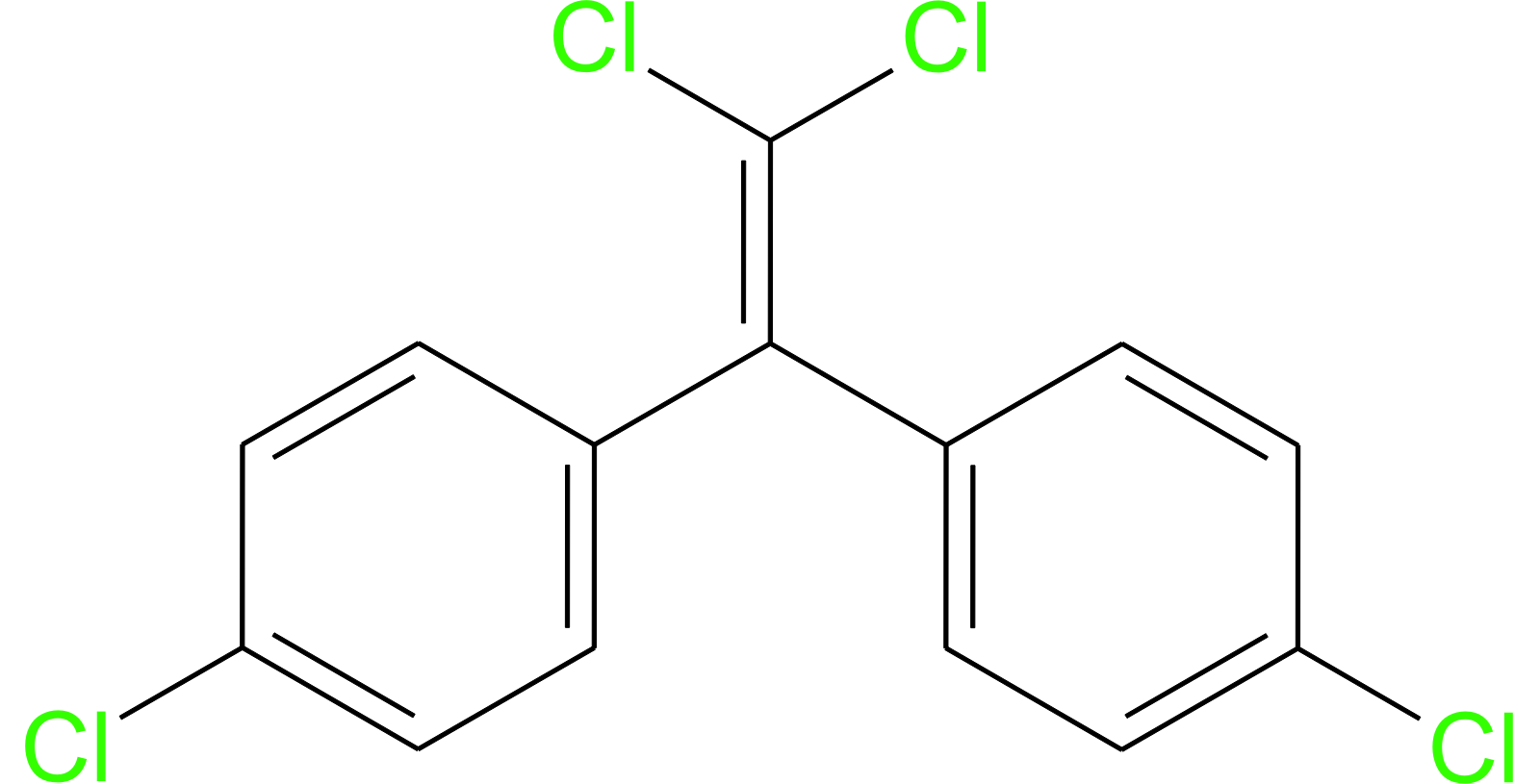

Supplement: Supplementary file 2 — ci4c02288_si_002.zip [file ci4c02288_si_002.zip › SI_v3_appendix/1184_DDE_GR/images/L_2d_main.png]

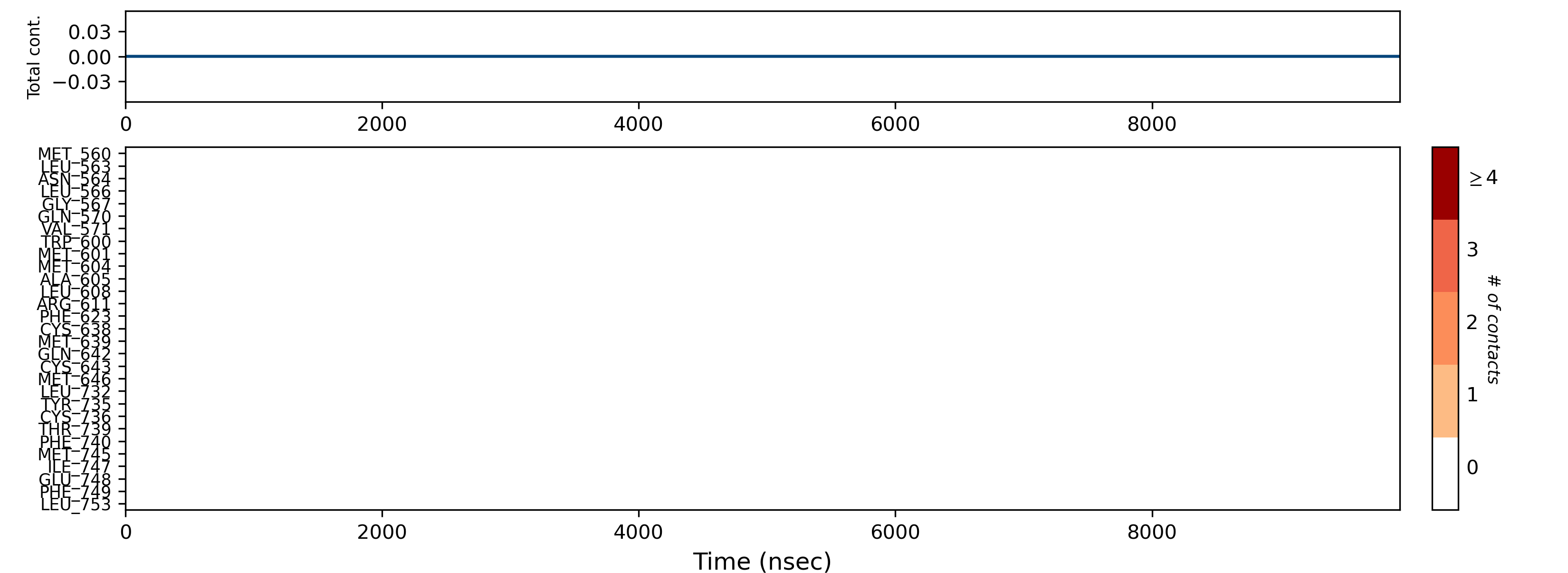

Supplement: Supplementary file 2 — ci4c02288_si_002.zip [file ci4c02288_si_002.zip › SI_v3_appendix/1184_DDE_GR/images/PL-Contacts_Timeline.png]

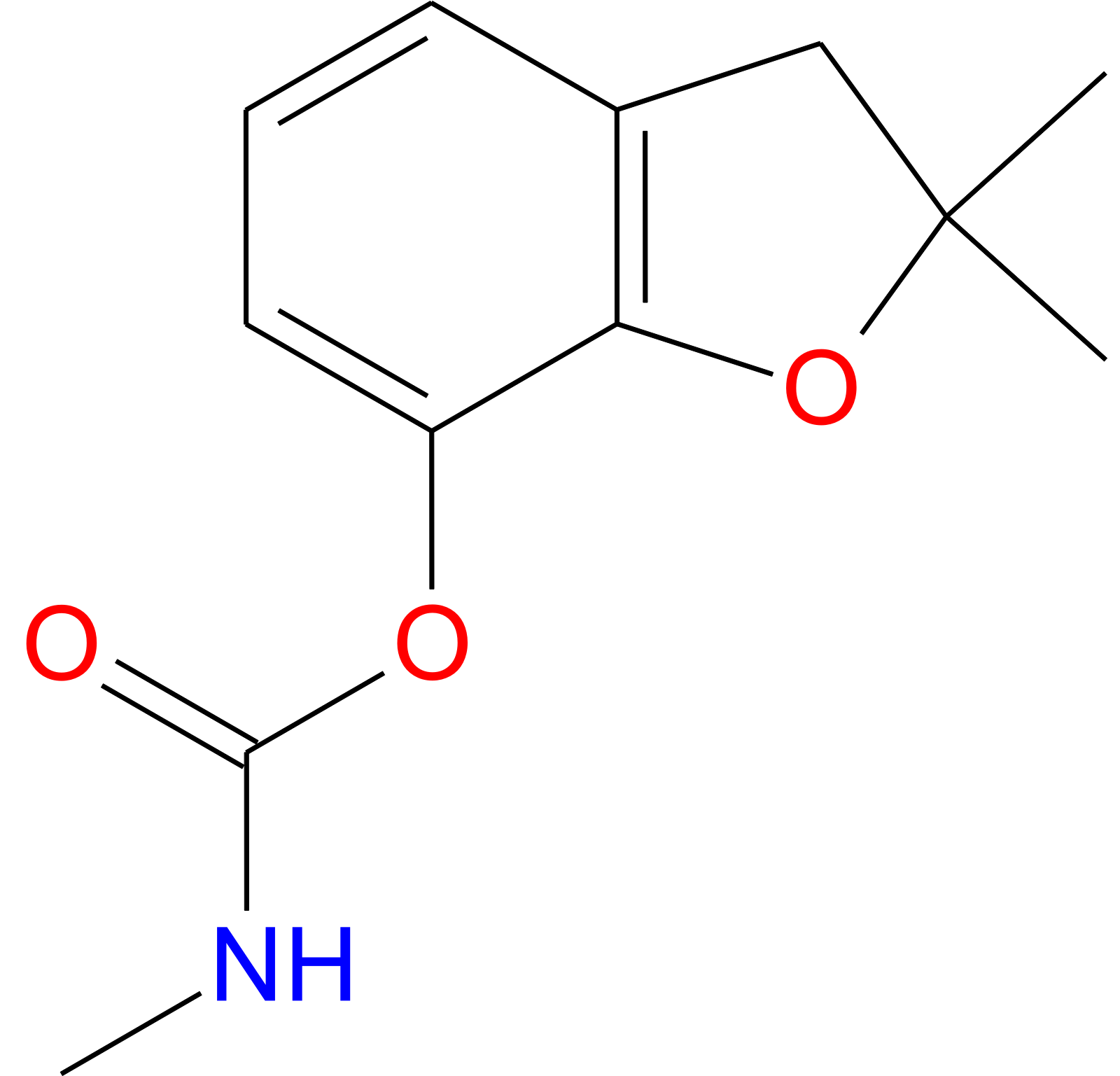

Supplement: Supplementary file 2 — ci4c02288_si_002.zip [file ci4c02288_si_002.zip › SI_v3_appendix/1210_CF_ER/images/L_2d_main.png]

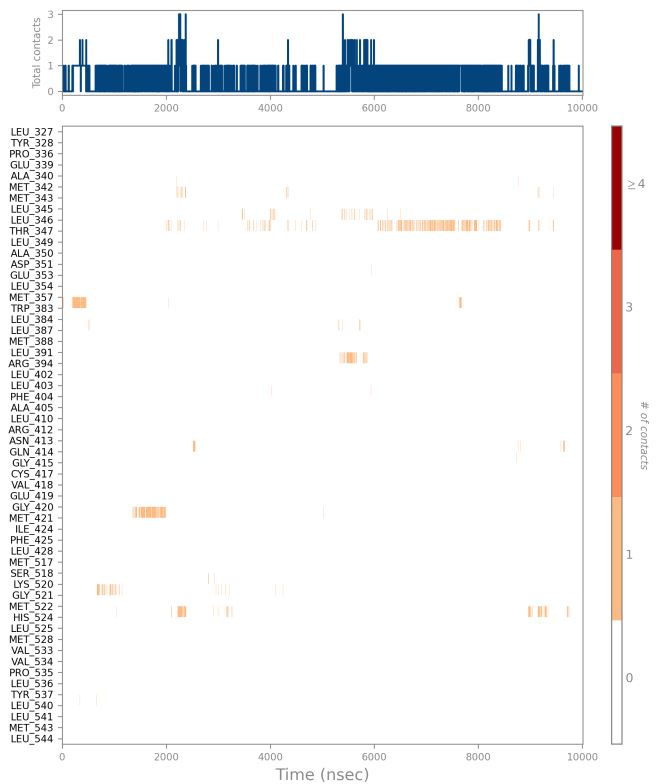

Supplement: Supplementary file 2 — ci4c02288_si_002.zip [file ci4c02288_si_002.zip › SI_v3_appendix/1210_CF_ER/images/PL_contact_Timeline.JPG]

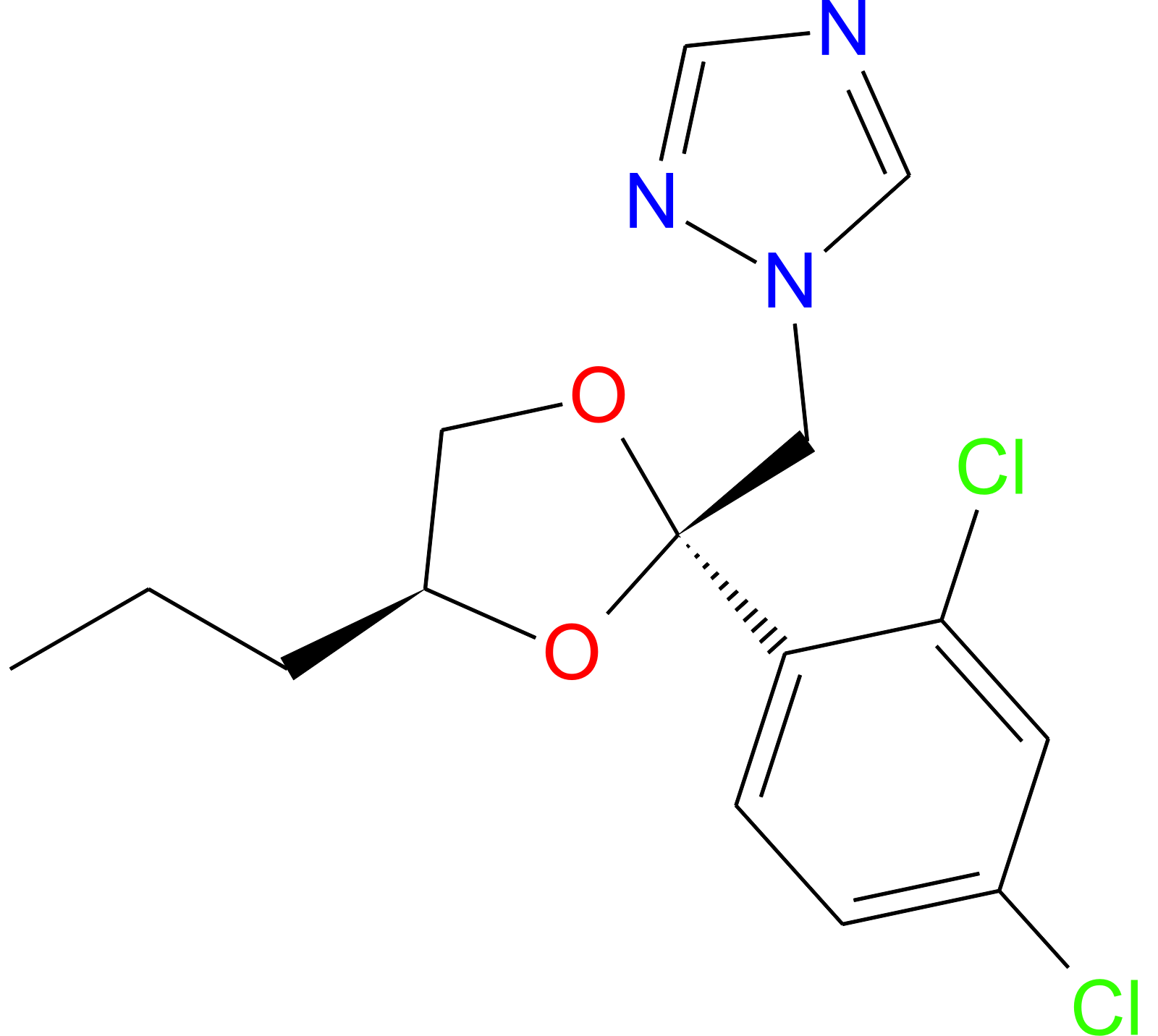

Supplement: Supplementary file 2 — ci4c02288_si_002.zip [file ci4c02288_si_002.zip › SI_v3_appendix/1211_RS_PROP_ER/images/L_2d_main.png]

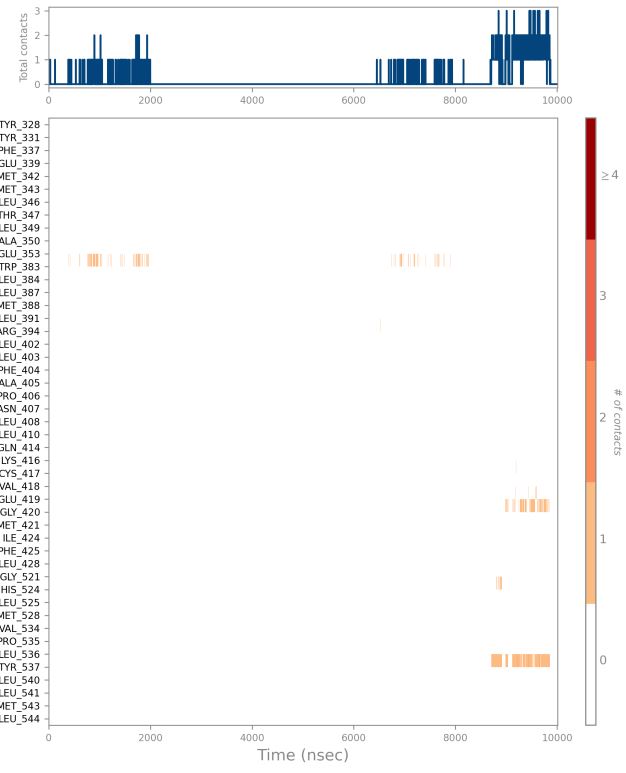

Supplement: Supplementary file 2 — ci4c02288_si_002.zip [file ci4c02288_si_002.zip › SI_v3_appendix/1211_RS_PROP_ER/images/PL_contact_Timeline.JPG]

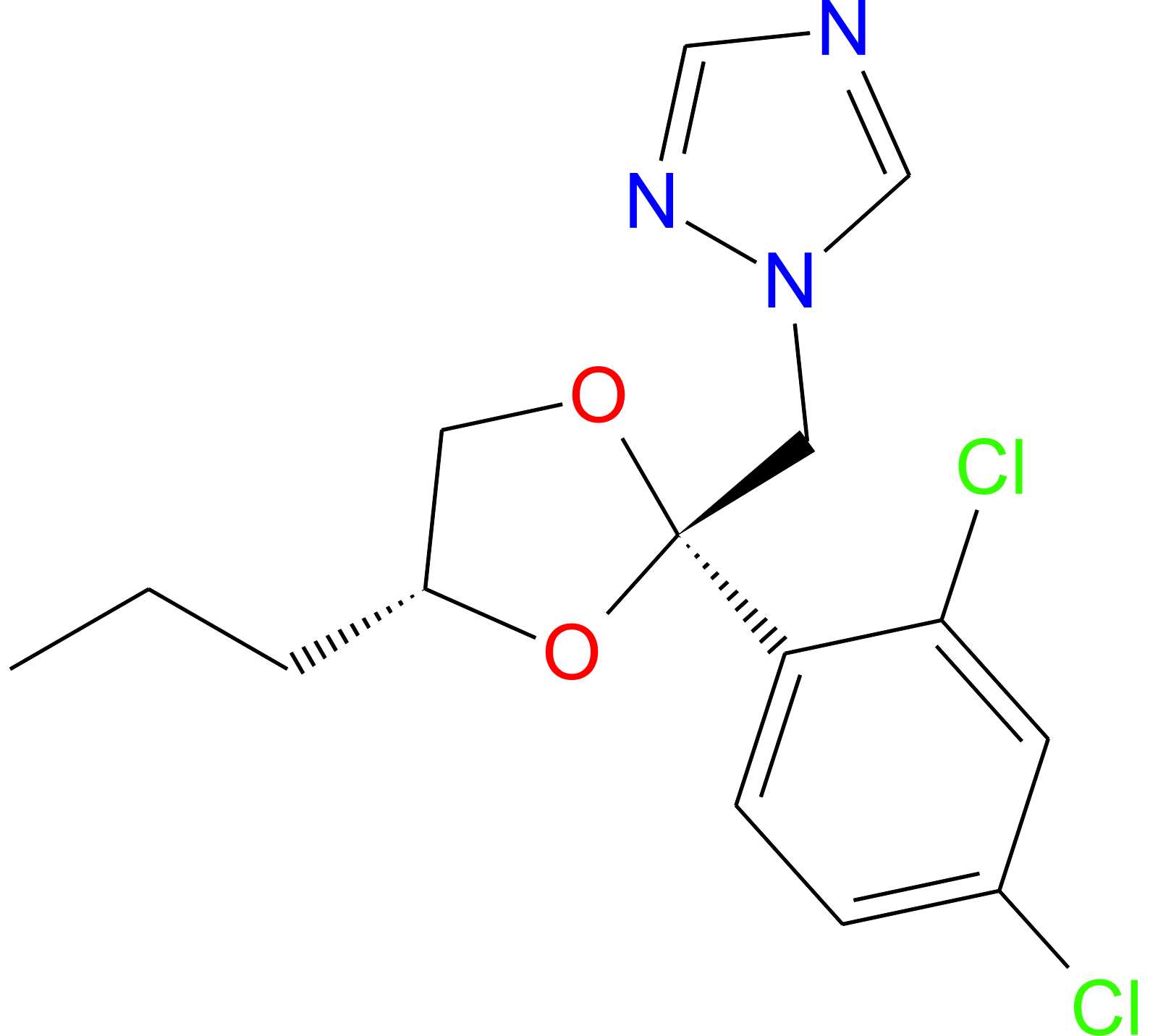

Supplement: Supplementary file 2 — ci4c02288_si_002.zip [file ci4c02288_si_002.zip › SI_v3_appendix/1212_RR_PROP_ER/images/L_2d_main.png]

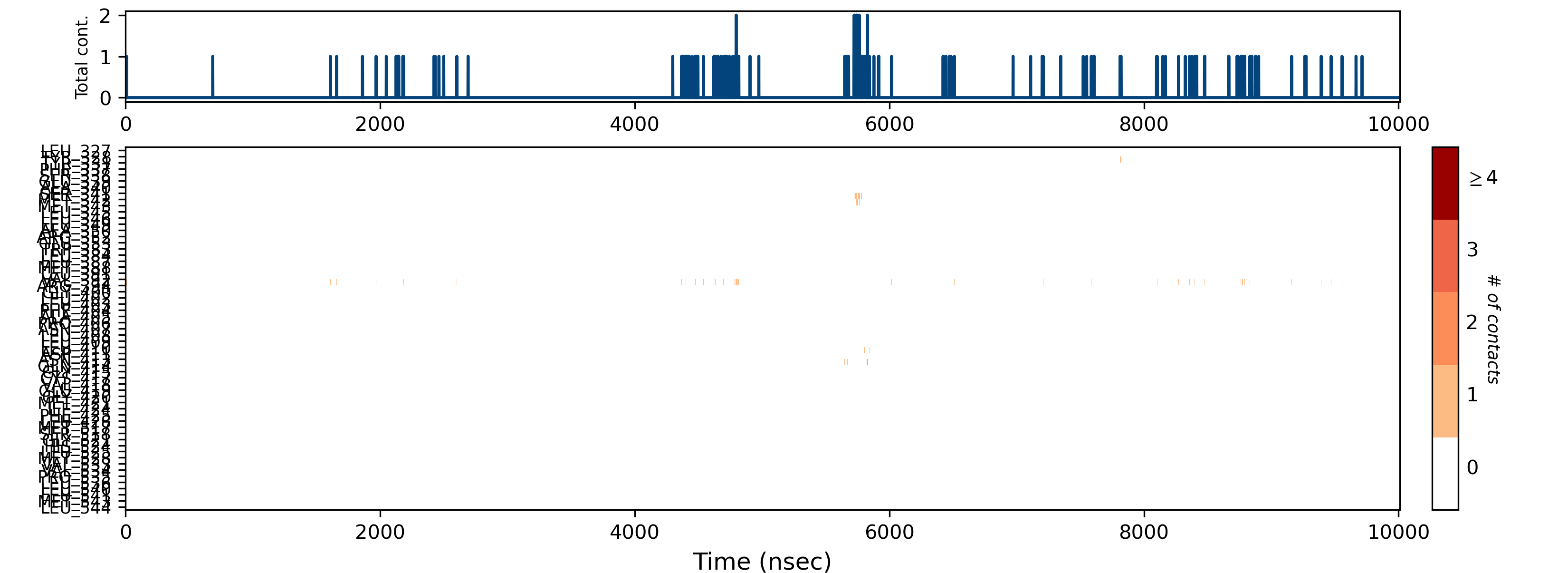

Supplement: Supplementary file 2 — ci4c02288_si_002.zip [file ci4c02288_si_002.zip › SI_v3_appendix/1212_RR_PROP_ER/images/PL-Contacts_Timeline.png]

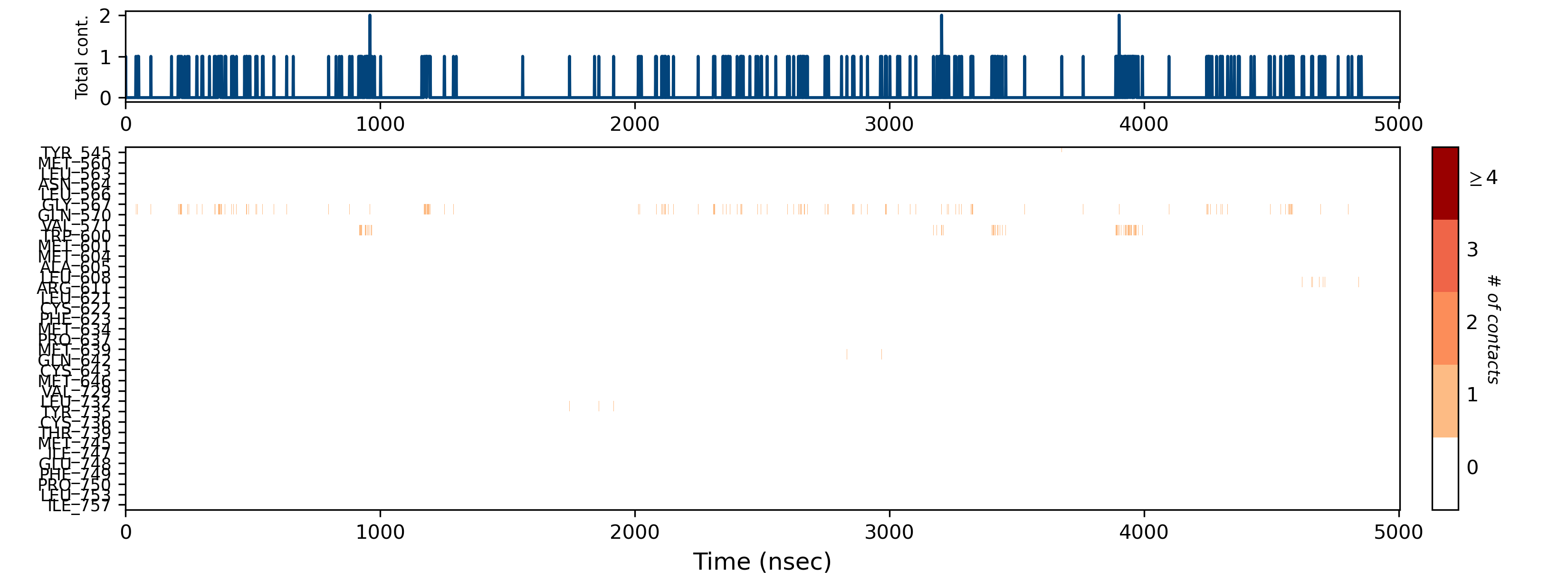

Supplement: Supplementary file 2 — ci4c02288_si_002.zip [file ci4c02288_si_002.zip › SI_v3_appendix/1255_RS_PROP_GR/images/PL-Contacts_Timeline.png]

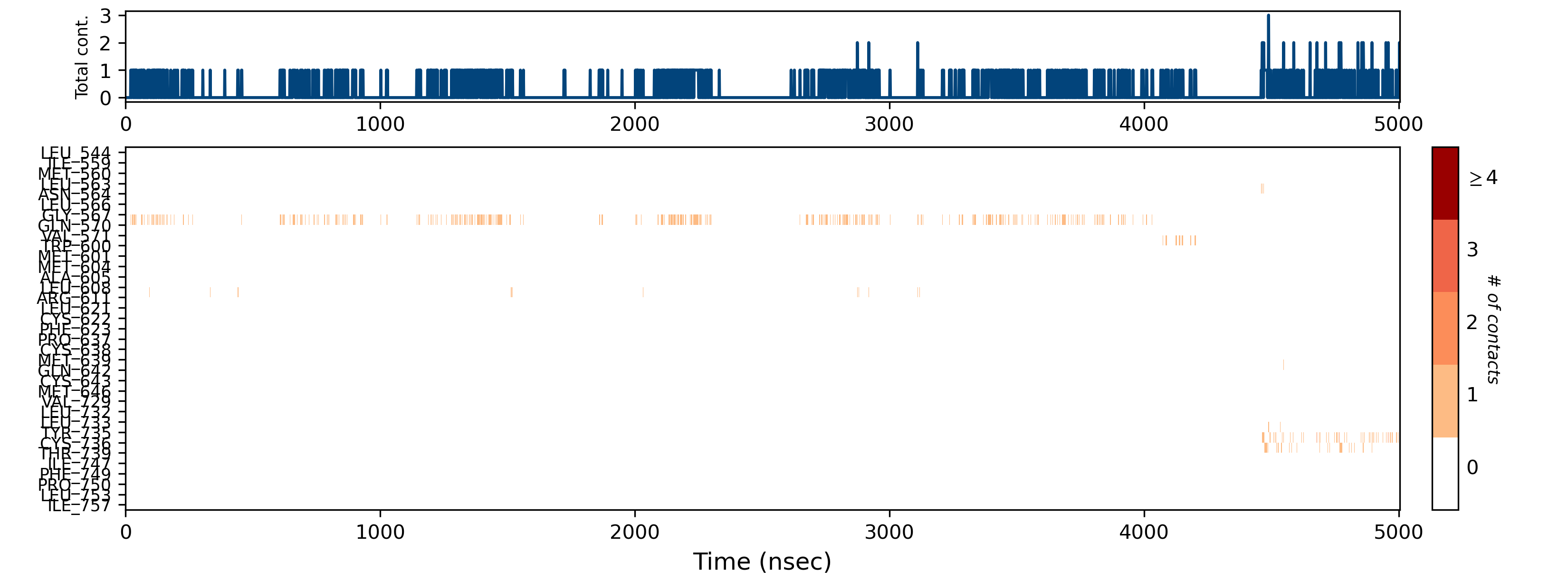

Supplement: Supplementary file 2 — ci4c02288_si_002.zip [file ci4c02288_si_002.zip › SI_v3_appendix/1256_RR_PROP_GR/images/PL-Contacts_Timeline.png]
