# Supplementary material for: Look What You Made Me Do: Discerning Feature for Classification of Endocrine-Disrupting Chemical Binding to Steroid Hormone Receptors
Source: J Chem Inf Model. 2025 Apr 9;65(8):4148–62. doi: 10.1021/acs.jcim.4c02288 (PMC12042260; doi:10.1021/acs.jcim.4c02288)
Supplement: Supplementary file 3 — ci4c02288_si_003.pdf [file ci4c02288_si_003.pdf]

SUPPORTING INFORMATION – Look what you made me do: Discerning feature for classification of Endocrine Disrupting Chemicals binding to Steroid Hormone Receptor.

Azam Rashidian[1,2*], Sini Pitkänen[3*], Vinicius Goncalves Maltarollo[4], Ulrich Schoppmeier[2], Ekaterina Shevchenko[1], Prasanthi Medarametla[5], Antti Poso[1,5], Jenni Küblbeck[3], Paavo Honkakoski[5#], Thales Kronenberger[1,2,5#]

[1] **a**. Department of Pharmaceutical and Medicinal Chemistry, Institute of Pharmaceutical Sciences, Eberhard-Karls-Universität, Tübingen, Auf der Morgenstelle 8, 72076 Tübingen, Germany. **b**. Tübingen Center for Academic Drug Discovery & Development (TüCAD$_2$), 72076 Tübingen, Germany.
[2.] Interfaculty Institute of Microbiology and Infection Medicine (IMIT), University of Tübingen, Tübingen, Germany; Partner-site Tübingen, German Center for Infection Research (DZIF), Tübingen, Germany.
[3.] A.I. Virtanen Institute for Molecular Sciences, University of Eastern Finland, P.O. Box 1627, FI-70210 Kuopio, Finland
[4] Departamento de Produtos Farmacêuticos, Faculdade de Farmácia, Universidade Federal de Minas Gerais, Av. Presidente Antônio Carlos, 6627, Pampulha, 31270-901 Belo Horizonte-MG, Brazil
[5] School of Pharmacy, Faculty of Health Sciences, University of Eastern Finland, 70211, Kuopio, Finland.

* Equal contribution, # Corresponding authors: T.K.: thales.kronenberger@uni-tuebingen.de, Elfriede-Aulhorn-Str. 6, 72076, Tübingen (Germany). P.H.: paavo.honkakoski@uef.fi

Table of contents **Page #**

**Figure S1**. Homology model concept for different simulated systems.	**S1**
**Table S1.** Summary of the simulated systems.	**S2**
**Figure S2.** Root mean square deviation (RMSD) of ERα's backbone	**S3**
**Figure S3.** Root mean square deviation (RMSD) of ERα's backbone with endoxifen	**S4**
**Figure S4.** Root mean square deviation (RMSD) of GR's backbone	**S5**
**Figure S5.** Potential binding modes for ERα- and GR-LBD bound to relevant ligands	**S6**
**Figure S6.** Potential binding modes for ERα- and GR-LBD bound to EDCs obtained from clustering the simulation trajectory. (BPA, BPA, DES)	**S7**
**Figure S7.** Potential binding modes for ERα- and GR-LBD bound to DEHP isomers	**S8**
**Figure S8.** Potential binding modes for ERα- and GR-LBD bound to MEHP and DDE	**S9**
**Figure S9.** Potential binding modes for ERα- and GR-LBD bound to 3OH-CF and CF	**S10**
**Figure S10.** Potential binding modes for ERα- and GR-LBD bound different propiconazole (PROP) isomers and CF	**S11**
**Table S2.** Population frequency for each cluster (C1-C5)	**S12**
**Figure S11** A timeline visulization of the H-bond for compounds bound to ER.17-β-Estradiol, $E2_{AGO}$ (A), 17-β-Estradiol, $E2_{AGO}$ (B).	**S13**
**Figure S12.** A timeline visulization of the H-bond for compounds bound to ER. MEHP (A), DES (B), BPA (C).	**S14**
**Figure S13.** A timeline visulization of H-bonds for compounds bound to ER. 3OH-CF (A). BPC_{AGO} (B). BPC_{ANT} (C).	**S15**
**Figure S14** A timeline visulization of the H-bond for compounds bound to ER. Endoxifen, $ENDO_{AGO}$ (A), Endoxiphen, $ENDO_{ANT}$ (B).	**S16**
**Figure S15.** A timeline visulization of the H-bond for compounds bound to GR. GR-DEX_{AGO} (A). GR-DEX_{ANT} (B).	**S17**
**Figure S16.** A timeline visulization of the H-bond for compounds bound to GR. MEHP (A). DES (B). BPA (C).	**S18**
**Figure S17.** A timeline visulization of the H-bond for compounds bound to GR. 3OH-CF (A) and CF (B).	**S19**
**Figure S18.** A timeline visulization of the H-bond for compounds bound to GR. RU-486_{AGO} (A) and RU-486_{ANT} (B).	**S20**
**Table S3.** Protein-ligand interaction in the GR.	**S21**
**Table S4.** ERα protein-ligand interactions.	**S22**
**Table S5**. Predicted binding energy for the EDCs in the ERα simulations	**S23**

SUPPORTING INFORMATION

**Table S6**. Predicted binding energy for the EDCs in the GR simulations.	**S24**
**Table S7**. Coulombic energy terms of the predicted binding energy (kcal/mol) for individual replicas	**S25**
**Figure S19**. Ligand efficiency prediction for the ERα and GR simulations displayed as violin plots.	**S26**
**Figure 20**. H12 folding plays an important role in the SHR activation.	**S27**
**Figure S21.** Root mean square fluctuation (RMSF) of the protein's backbone separated by residue and highlighted by secondary structure for ERα (A-C) and GR (D,E).	**S28**
**Figure S22** Root mean square fluctuation (RMSF) of the protein's backbone separated by residue and highlighted by helices for ERα with different EDC ligands.	**S29**
**Figure S23.** Root mean square fluctuation (RMSF) of the protein's backbone separated by residue and highlighted by helices for GR with different EDC ligands	**S30**
**Figure S24.** Protein's backbone root mean square fluctuation (RMSF) for specific helices.	**S31**
**Figure S25**. Distance plots between relevant helices.	**S32**
**Figure S26.** H12 relevant angle variation.	**S33**
**Figure S27.** Variation of the Ang1 vs Ang2 along the simulations of ERα bound to EDCs	**S34**
**Figure S28.** Variation of the Ang1 vs Ang2 along the simulations of GR bound to different EDCs	**S35**
**Table S8.** Mean and standard deviation values for the pair-wise distances (Å) between the center of masses of helices H10, H3 and H12 and Ang1 and Ang2 for simulations.	**S36**
**Table S9.** Ligand properties averaged along the simulation time.	**S37**
**Table S10.** Fractional contribution of the top 5 variables for each principal component.<br>H = helix, L = loop, βa = beta-sheet, h = hydrophobic interactions, p=polar	**S38**
**Figure S29**. Unsupervised neural network Self-Organizing Maps (SOM)	**S38**
**Figure S30.** Correlation between predicted binding energy and EC50 values**.**	**S39**
**Figure S31**. Amino acids with relevant interactions on SHRs-EDCs	**S39**
**Extended methods/results on the statistical analyses**	**S40 - Extra**

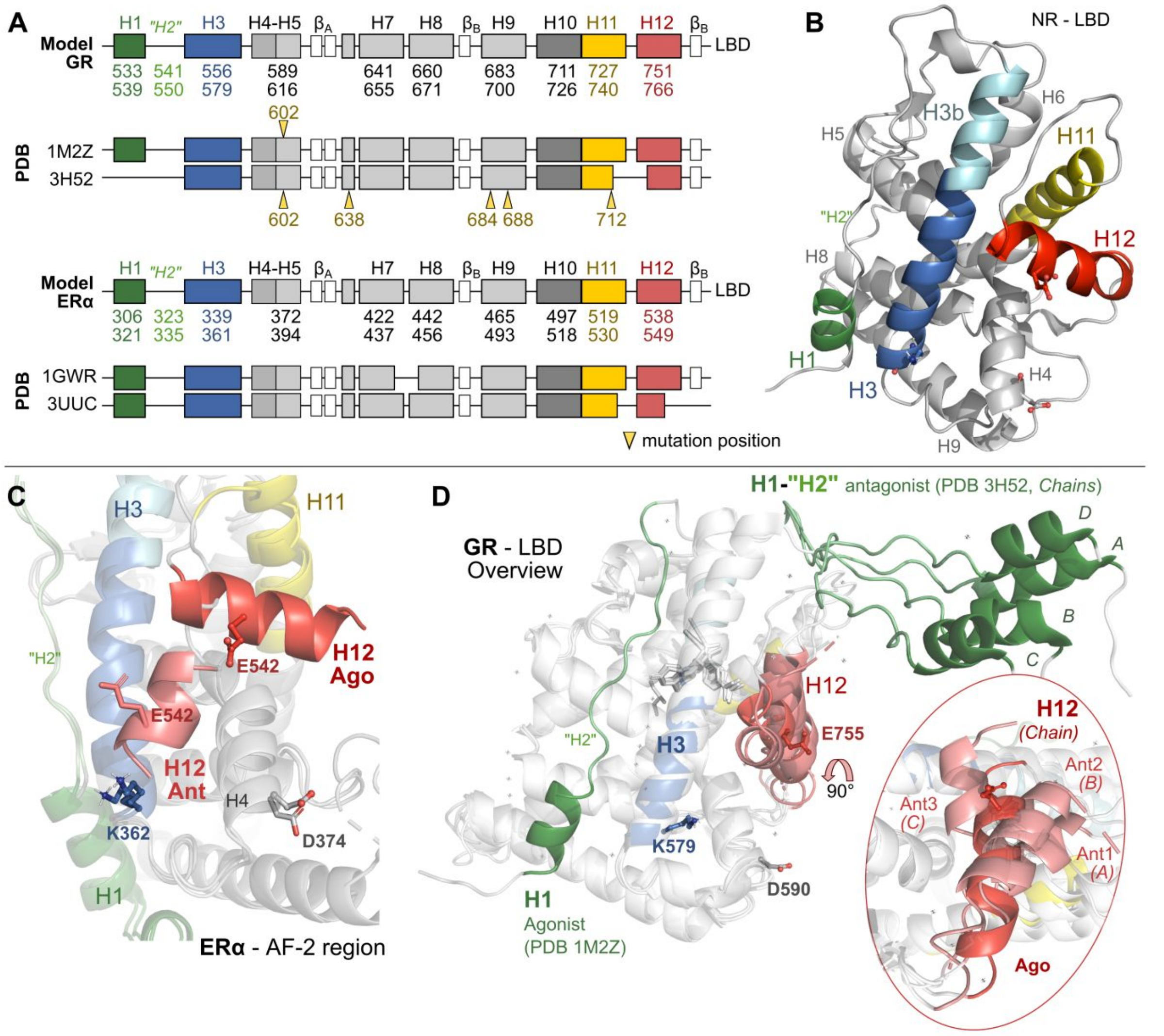

Figure S1. Homology model concept for different simulated systems. A) schematic representation of ERα and GR modelled sequences and their respective templates, colored according to their structural motifs (displayed in B). H2 is not present in the respective receptors, but its position in the general NR architecture's sequence is here highlighted. H3b depicts the half of H3 which is closer to the H11 and later used for references in distance calculations. Mutation sites deviating from the UniProt canonical sequences are highlighted in yellow. Overview of the different crystal structures employed to generate the antagonist models (C, ERα, PDB 3UU7, chain B) and (D, GR, PDB ID: 3H52), displaying the aligned antagonistic structure individual chains against a representative chain from the agonist-bound structure.

Table S1. Summary of the simulated systems displaying their respective CAS number and simulation time (in μs) in ERα and GR proteins. RU-486: mifepristone, E2: 7β-estradiol, DEX: dexamethasone, DES: diethylstilbestrol, DEHP: bis(2-ethylhexyl) phthalate, MEHP: mono(2-ethylhexyl) phthalate, DDE: dichlorodiphenyldichloroethylene, BPA: bisphenol A, BPC: bisphenol C, PROP: propiconazole, CF: carbofuran, 3OH-CF: 3OH-carbofuran, ENDO: endoxifen. Along with the text, AGO and ANT subscripts denotes for agonist and antagonist receptor conformation, respectively pointing to the agonist and antagonist receptor conformation.

**Ligand ERα**	**Ligand GR**	**Receptor conformation**	**CAS-Number**	**Sim. Time (μs)**	
				**ERα**	**GR**
17β-E2	DEX	Agonist		14 μs (7x2 μs)	10 μs (5x2 μs)
17β-E2	DEX	Antagonist		10 μs (5x2 μs)	10 μs (5x2 μs)
(*RR*)-DEHP	(*RR*)-DEHP	Agonist	117-81-7	10 μs (5x2 μs)	5 μs (5x 1μs)
(*RS*)-DEHP	(*RS*)-DEHP	Agonist	117-81-7	10 μs (5x2 μs)	5 μs (5x 1μs)
(SS)-DEHP	(SS)-DEHP	Agonist	117-81-7	10 μs (5x2 μs)	5 μs (5x 1μs)
MEHP	MEHP	Agonist	4376-20-9	5 μs (5x1 μs)	5 μs (5x 1μs)
DES	DES	Agonist	56-53-1	10 μs (5x2 μs)	10 μs (5x 2μs)
BPA	BPA	Agonist	80-05-7	10 μs (5x2 μs)	5 μs (5x 1μs)
DDE	DDE	Agonist	72-55-9	10 μs (5x2 μs)	10 μs (5x 2μs)
(RS)-PROP	(RS)-PROP	Agonist	60207-90-1	10 μs (5x2 μs)	5 μs (5x 1μs)
(RR)-PROP	(RR)-PROP	Agonist	60207-90-1	10 μs (5x2 μs)	5 μs (5x 1μs)
3OH-CF	3OH-CF	Agonist	16655-82-6	5 μs (5x2 μs)	10 μs (5x2 μs)
CF	CF	Agonist	1563-66-2	10 μs (5x2 μs)	5 μs (5x 1μs)
BPC	---	Agonist	137525-51-0	10 μs (5x2 μs)	---
BPC	---	Antagonist	137525-51-0	10 μs (5x2 μs)	---
ENDO	RU-486	Agonist	110025-28-0	25 μs (10x2.5 μs)	10 μs (5x2 μs)
ENDO	RU-486	Antagonist	110025-28-0	25 μs (10x2.5 μs)	10 μs (5x2 μs)

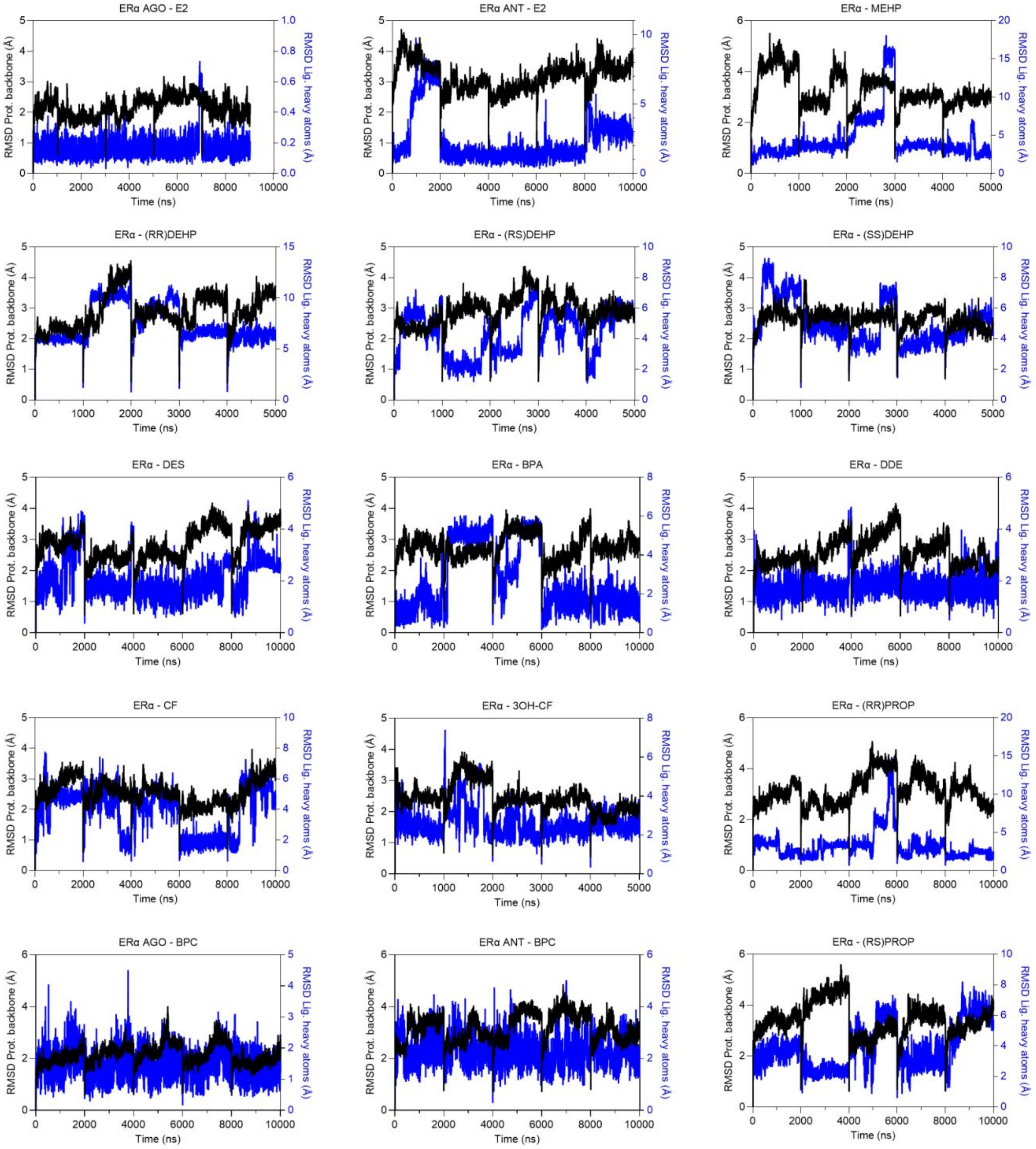

Figure S2. Root mean square deviation (RMSD) of ERα's backbone (black line) and each simulated ligand's heavy atoms (blue line) along the simulation time (ns). Data derived from the concatenated trajectory (see Table S1 for reference).

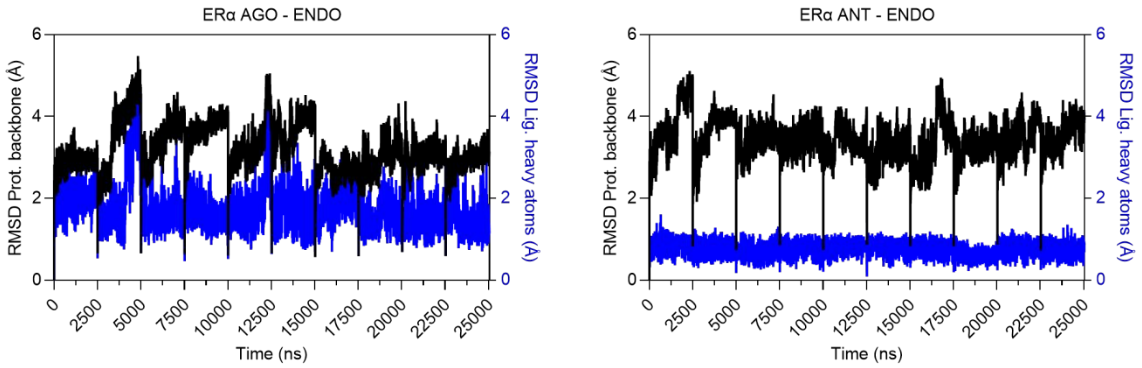

Figure S3. Root mean square deviation (RMSD) of ERα's backbone (black line) and each simulated ligand's heavy atoms (blue line) along the simulation time (ns). Data derived from the concatenated trajectory (10x2,500 ns). Endoxifen was simulated in both the modelled ERα agonist (PDB 1GWR, right) and in its original co-crystallized antagonist conformation (PDB 3ERT, left)

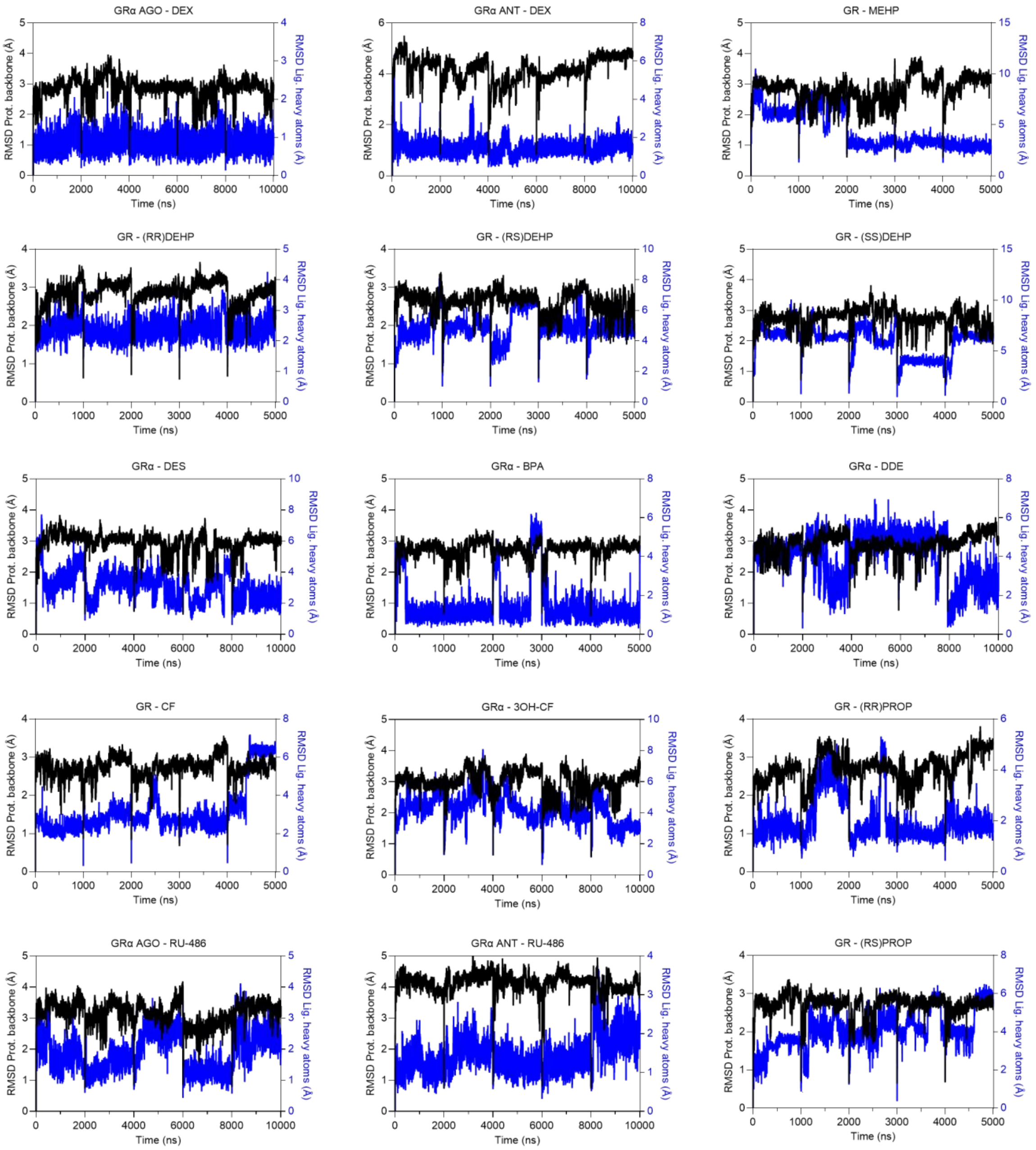

Figure S4. Root mean square deviation (RMSD) of GR's backbone (black line) and each simulated ligand's heavy atoms (blue line) along the simulation time (ns). Data derived from the concatenated trajectory (either 5x1 μs or 5x2 μs – see Table S1 for reference).

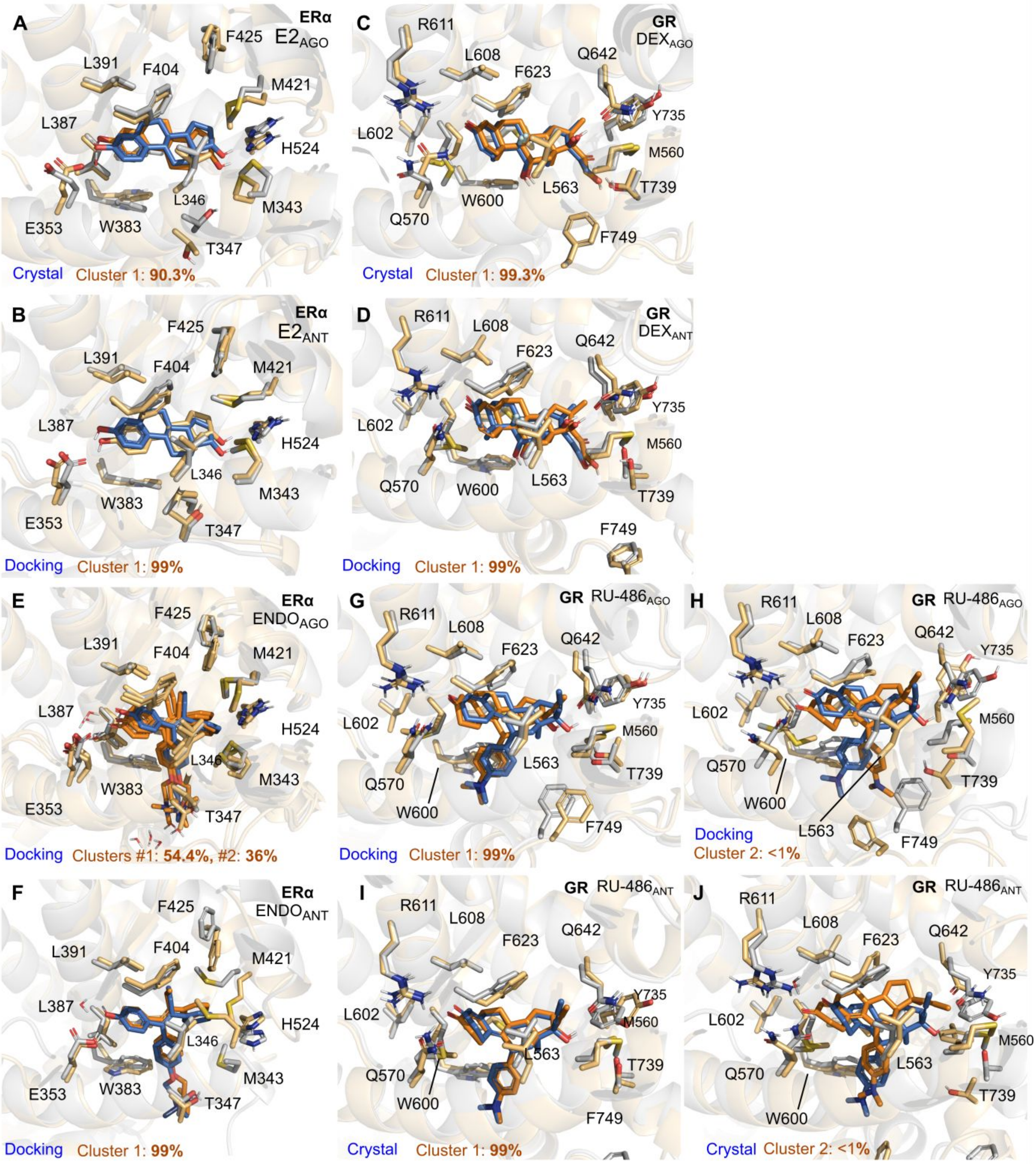

Figure S5. Potential binding modes for ERα- and GR-LBD bound to relevant ligands (E2, DEX, RU-486 and ENDO, see Table S1 for details on naming) obtained from clustering the simulation trajectory. Clusters (potential binding mode, in orange) superimposed over either crystal structure or the initial docking pose (in blue). (A) ERα-E2 (or 17β-estradiol) $_{AGO}$. (B) ERα-E2 $_{ANT}$. (C) GR-DEX $_{AGO}$. (D) GR-DEX $_{ANT}$. (E) ERα -ENDO $_{AGO}$. (F) ERα -ENDO $_{ANT}$. (G) GR-RU-486 $_{AGO}$ cluster 1. (H) GR-RU-486 $_{AGO}$ cluster 2. (I) GR-RU-486 $_{ANT}$ cluster 1. (J) GR-RU-486 $_{ANT}$ cluster 2.

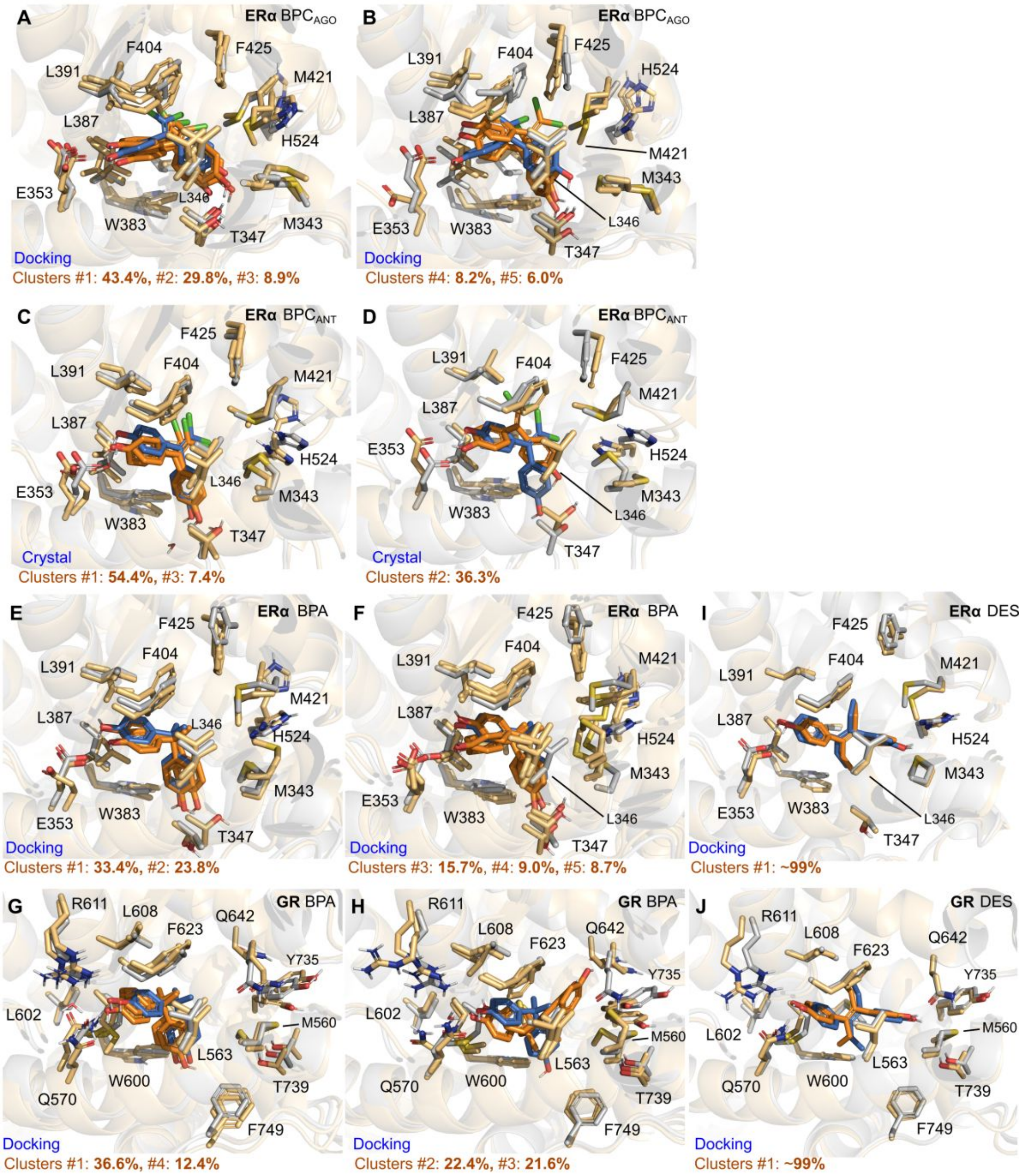

Figure S6. Potential binding modes for ERα- and GR-LBD bound to EDCs obtained from clustering the simulation trajectory. Clusters (potential binding mode in orange) superimposed over either crystal structure or the initial docking pose (in blue). Observed cluster conformation frequency is labelled in orange. (A, B) ERα-BPC $_{AGO}$. (C, D) ERα-BPC $_{ANT}$. (E, F) ERα-BPA. (G, H) GR-BPA. (I) ERα-DES. (J) GR-DES.

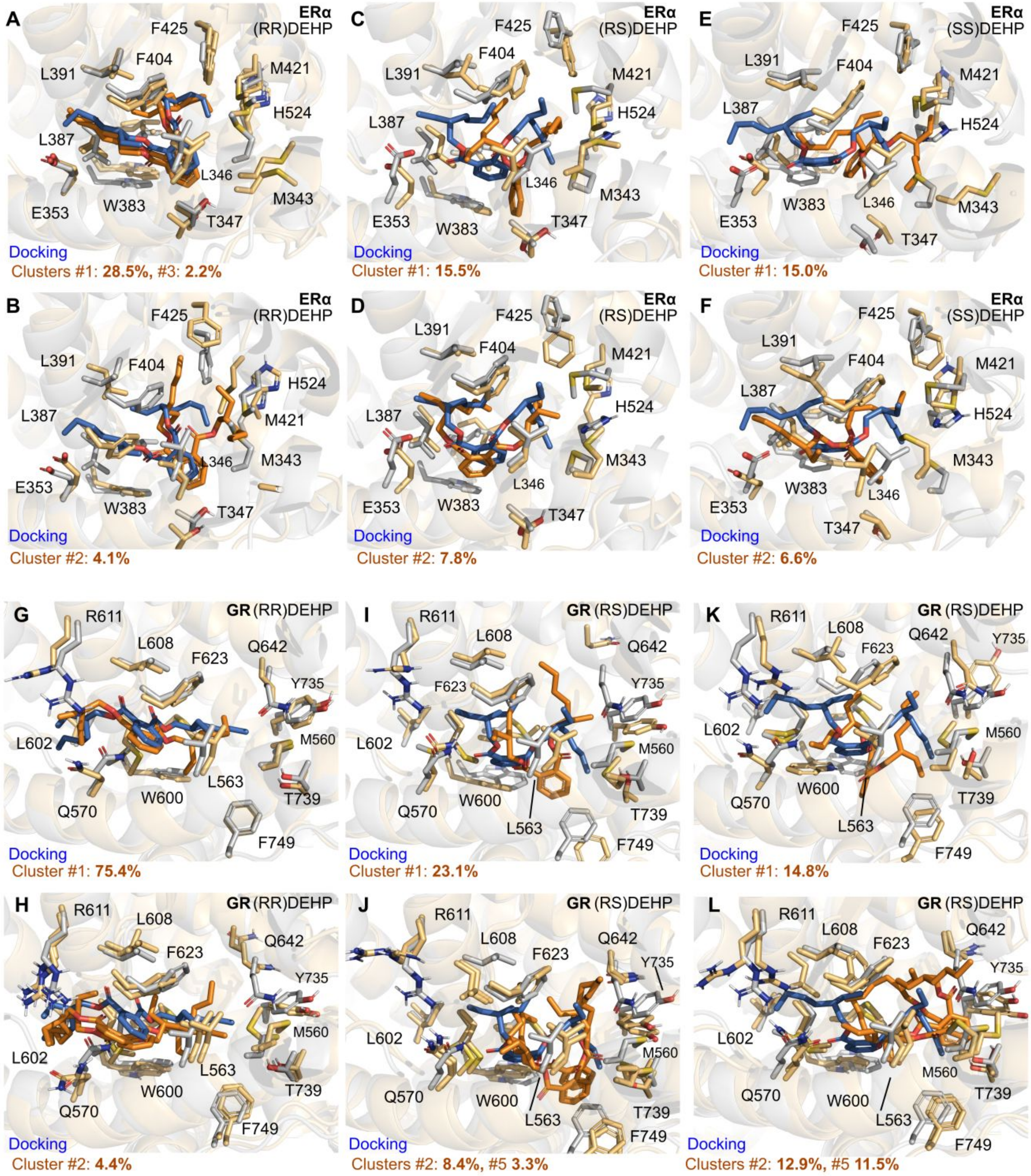

Figure S7. Potential binding modes for ERα- and GR-LBD bound to DEHP isomers obtained from clustering the simulation trajectory. Clusters (potential binding mode in orange) superimposed over either crystal structure or the initial docking pose (in blue). Observed cluster conformation frequency is labelled orange. Clusters superimposed over the initial docking pose. Labelled as follows: (A-F) ERα-DEHP and (G-L) GR-DEHP.

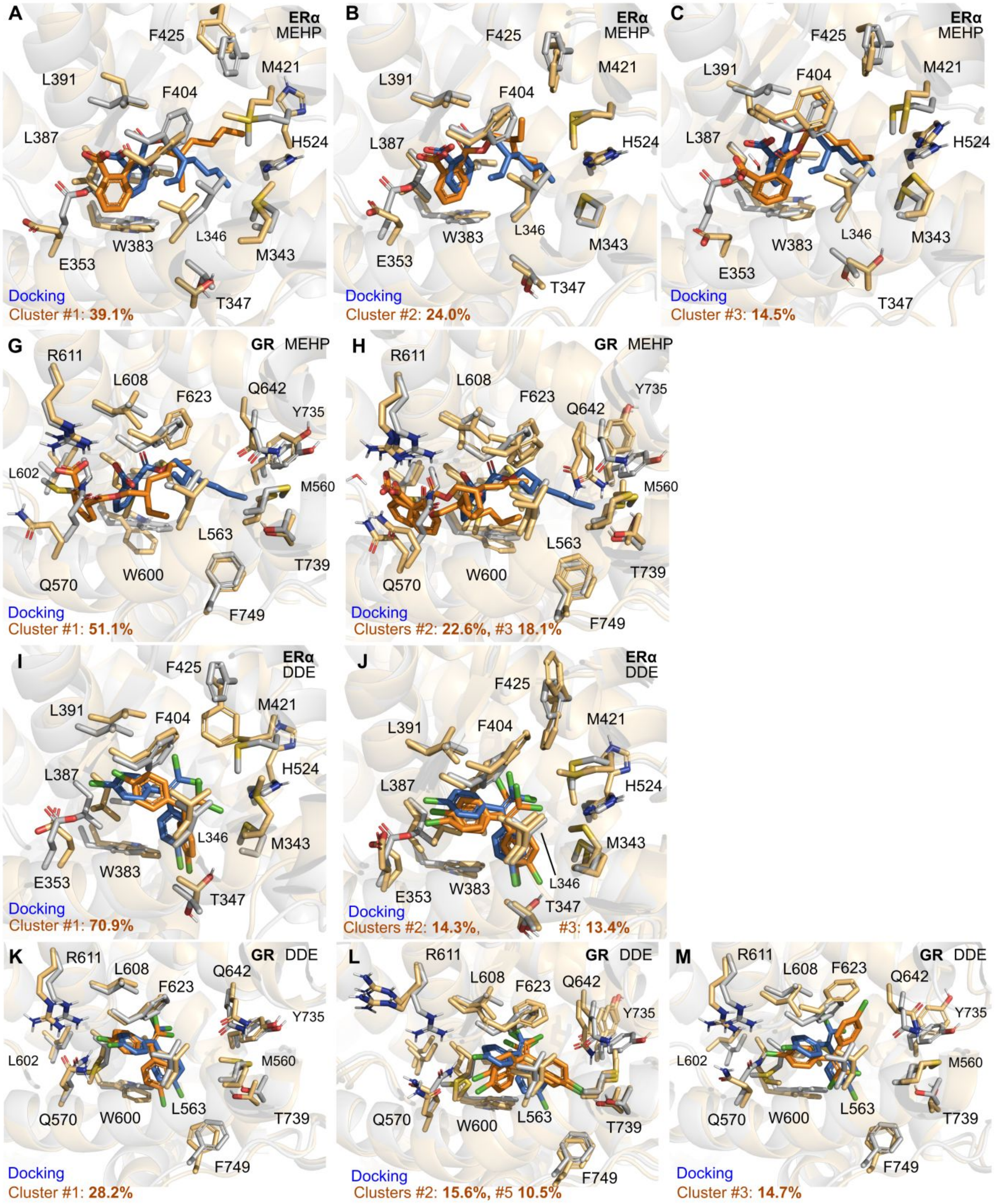

Figure S8. Potential binding modes for ERα- and GR-LBD bound to MEHP and DDE obtained from clustering the simulation trajectory. Clusters (potential binding mode in orange) superimposed over either crystal structure or the initial docking pose (in blue). Observed cluster conformation frequency is labelled orange. Labelled as follows (A-C) ERα-MEHP, (G,H) GR-MEHP, (I,J) ERα-DDE, (K-M) GR-DDE.

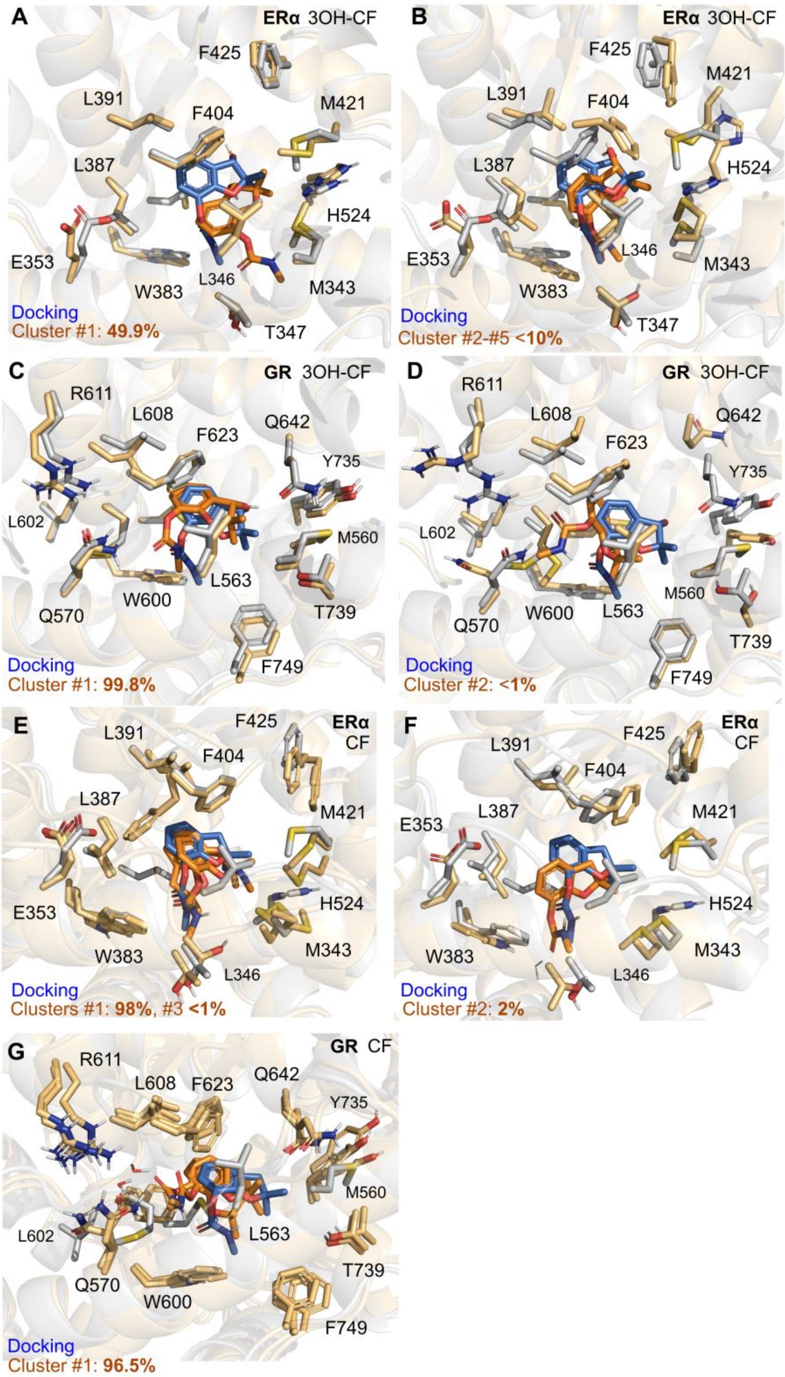

Figure S9. Potential binding modes for ERα- and GR-LBD bound to 3OH-CF and CF obtained from clustering the simulation trajectory. Clusters (potential binding mode in orange) superimposed over either crystal structure or the initial docking pose (in blue). Observed cluster conformation frequency is labelled orange. Labelled as follows: (A,B) ERα- 3OH-CF, (C,D) GR-3OH-CF, (E,F) ERα-CF and (G) GR-CF.

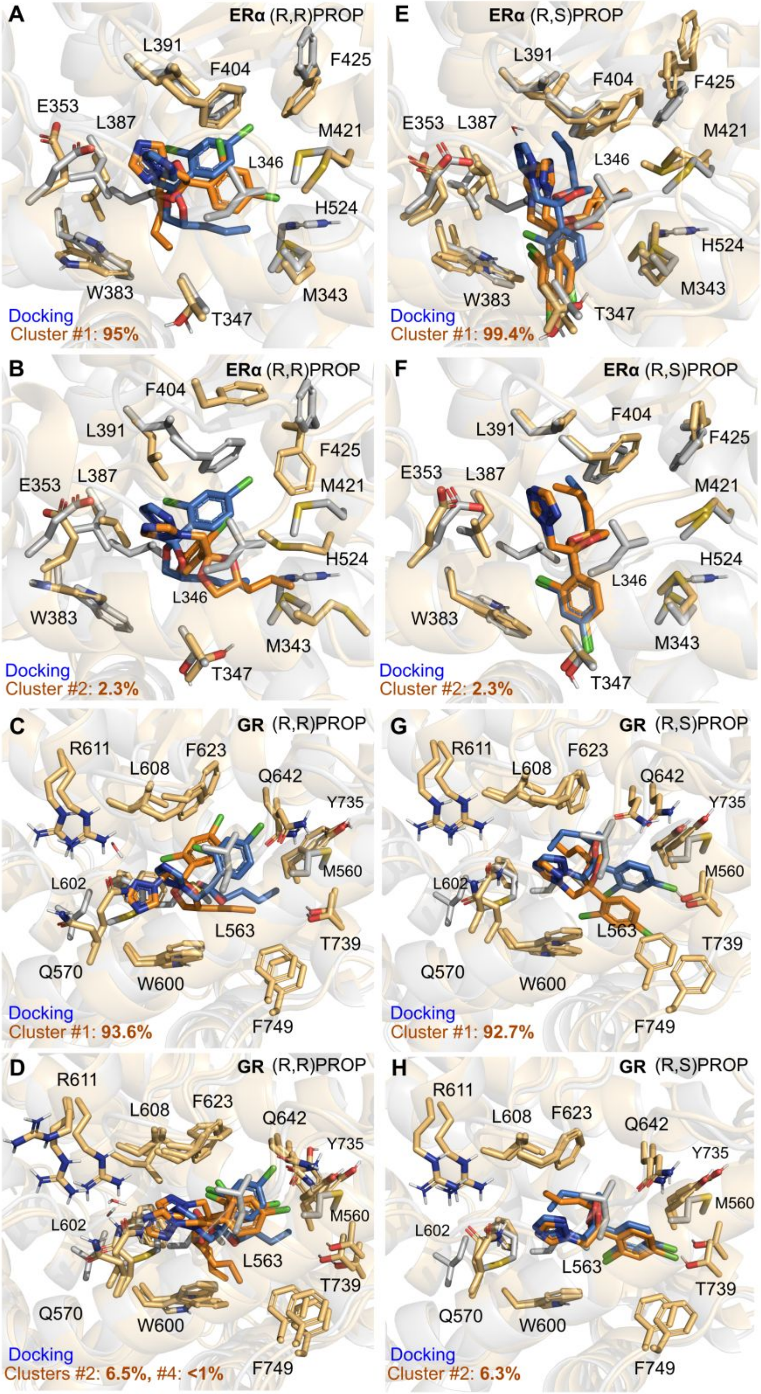

Figure S10. Potential binding modes for ERα- and GR-LBD bound different propiconazole (PROP) isomers and CF obtained from clustering the simulation trajectory. Clusters (potential binding mode in orange) superimposed over either crystal structure or the initial docking pose (in blue). Observed cluster conformation frequency is labelled in orange. Labelled as follows: (R,R)PROP bound to ERα (A,B) or GR (C,D) and (R,S)PROP bound to ERα (E,F) or GR-CF (G,H).

Table S2. Population frequency for each cluster (C1-C5), meaning, how often does that conformation appear in the simulation, and their RMSD comparison to the initial docking pose or crystal structure.

	C1		C2		C3		C4		C5			C1		C2		C3		C4		C5	
ERa	%	RMS	%	RMS	%	RMS	%	RMS	%	RMS	GR	%	RMS	%	RMS	%	RMS	%	RMS	%	RMS
17β-E2$_{AGO}$	90.3	0.6	9.8	1.5							DEX$_{AGO}$	100.1	0.3								
17β-E2$_{ANT}$	99.0	0.6									DEX$_{ANT}$	100.1	0.9								
(RR)-DEHP	28.5	0.9	4.1	6.3	2.2	1.0	1.7	6.8	1.4	5.0	(RR)-DEHP	75.4	2.2	4.4	2.8	1.6	2.9	0.8	3.1	0.8	1.9
(RS)-DEHP	15.5	4.0	7.8	2.2	5.6	5.2	4.3	5.9	4.1	5.9	(RS)-DEHP	23.1	4.0	8.4	4.5	8.4	6.3	8.0	3.9	3.3	4.6
(SS)-DEHP	15.0	5.6	6.6	3.9	5.6	5.6	5.3	4.7	2.0	6.7	(SS)-DEHP	14.8	4.6	12.9	6.8	11.5	6.6	5.3	6.4	5.1	6.4
MEHP	39.1	2.7	24.0	1.8	14.5	2.9	3.7	3.1	2.9	3.2	MEHP	51.1	5.1	22.6	5.4	18.1	6.5	1.0	3.9	0.7	7.0
DES	99.0	0.7	<1	4.0	<1	1.2	<1	2.0	<1		DES	100.1	1.0								
BPA	33.4	1.4	23.8	0.4	15.7	1.8	9.0	2.1	8.7	0.8	BPA	33.6	1.2	22.4	4.3	21.6	3.4	12.4	1.6	5.2	1.9
DDE	70.9	1.2	14.3	1.2	13.4	2.3	1.0	1.8	0.3	3.5	DDE	28.2	1.0	15.6	2.4	14.7	4.6	12.5	3.4	10.5	2.2
(RS)-PROP	99.4	2.5	0.4	0.8	0.2	3.1	0.1	2.6	0.1	3.1	(RS)-PROP	92.7	2.0	6.3	0.7	1.0	1.9				
(RR)-PROP	95.0	2.9	2.3	3.8	1.8	3.5	0.5	1.7	0.2	1.5	(RR)-PROP	93.6	2.9	6.5	3.8	<1	3.5	<1	1.7	<1	1.5
3OH-CF	49.9	3.1	0.1	1.9	<1	4.8	<1	3.0	<1	3.2	3OH-CF	99.8	1.8	0.2	3.8	<1	4.5	<1	4.5	<1	3.0
CF	98.0	1.1	2.0	2.4	<1	4.4	<1	3.6	<1	5.9	CF	96.5	2.4	2.2	3.1	1.0	2.6	0.1	5.1		
BPC$_{AGO}$	43.4	1.4	29.8	1.3	8.9	1.6	8.2	2.8	6.0	2.4	RU-486$_{AGO}$	99.0	2.4	<1	1.1	<1	2.0				
BPC$_{ANT}$	54.4	0.4	36.3	2.1	4.2	1.3	2.8	1.8	2.4	3.1	RU-486$_{ANT}$	99.0	0.4	<1	1.2	<1	1.5				
ENDO$_{AGO}$	56.0		36.0		7.4		0.3		0.2												
ENDO$_{ANT}$	100.0																				

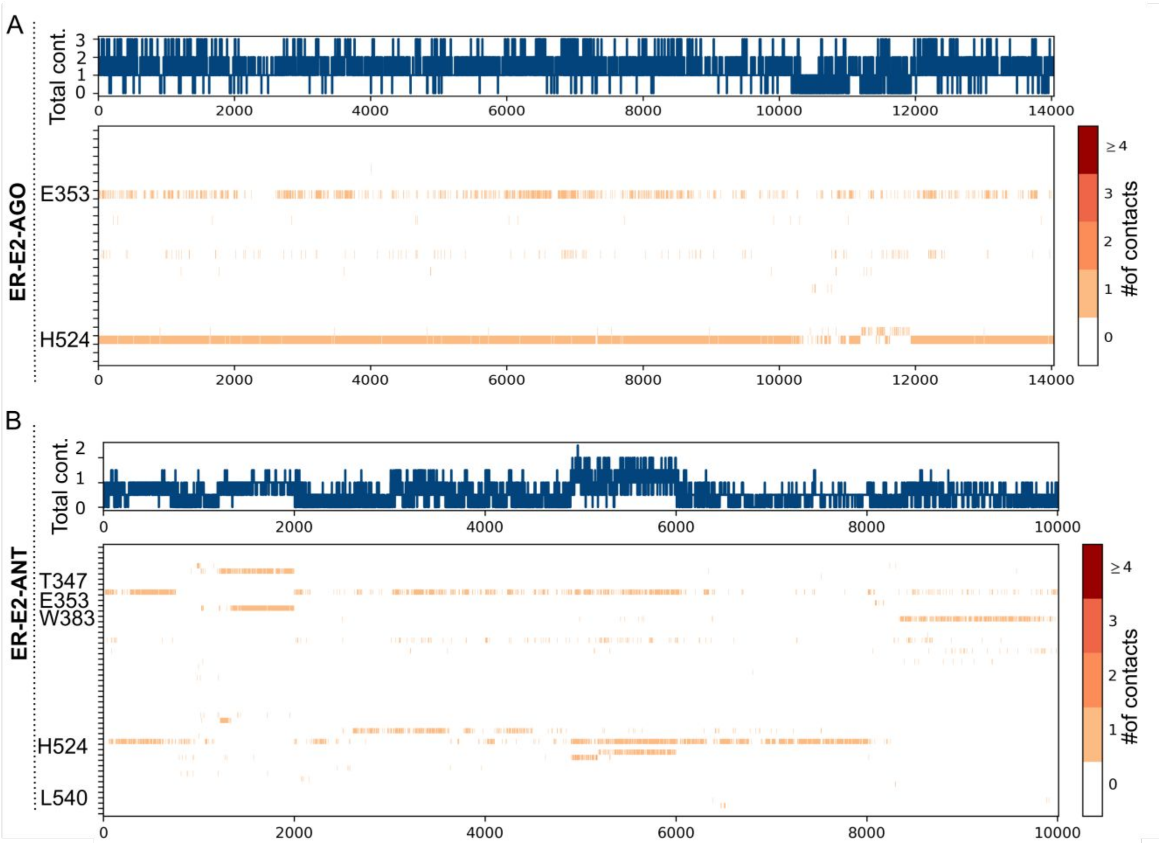

Figure S11. Timeline visulization of the H-bond for compounds bound to ER.17-β-Estradiol, $E2_{AGO}$ (A), 17-β-Estradiol, $E2_{AGO}$ (B). AGO and ANT denotes for agonist and agonist, respectively pointing to the agonist and antagonist receptor conformation. The top panel display the total number of contacts form between protein and ligand throughout the simulation time (graphs in blue). The bottom pannel highlight the residues that interact with the ligand at each trajectory frame. Residues that form multiple contacts with the ligand are indicated by darker shades of orange (shown on the scale to the right of plot).

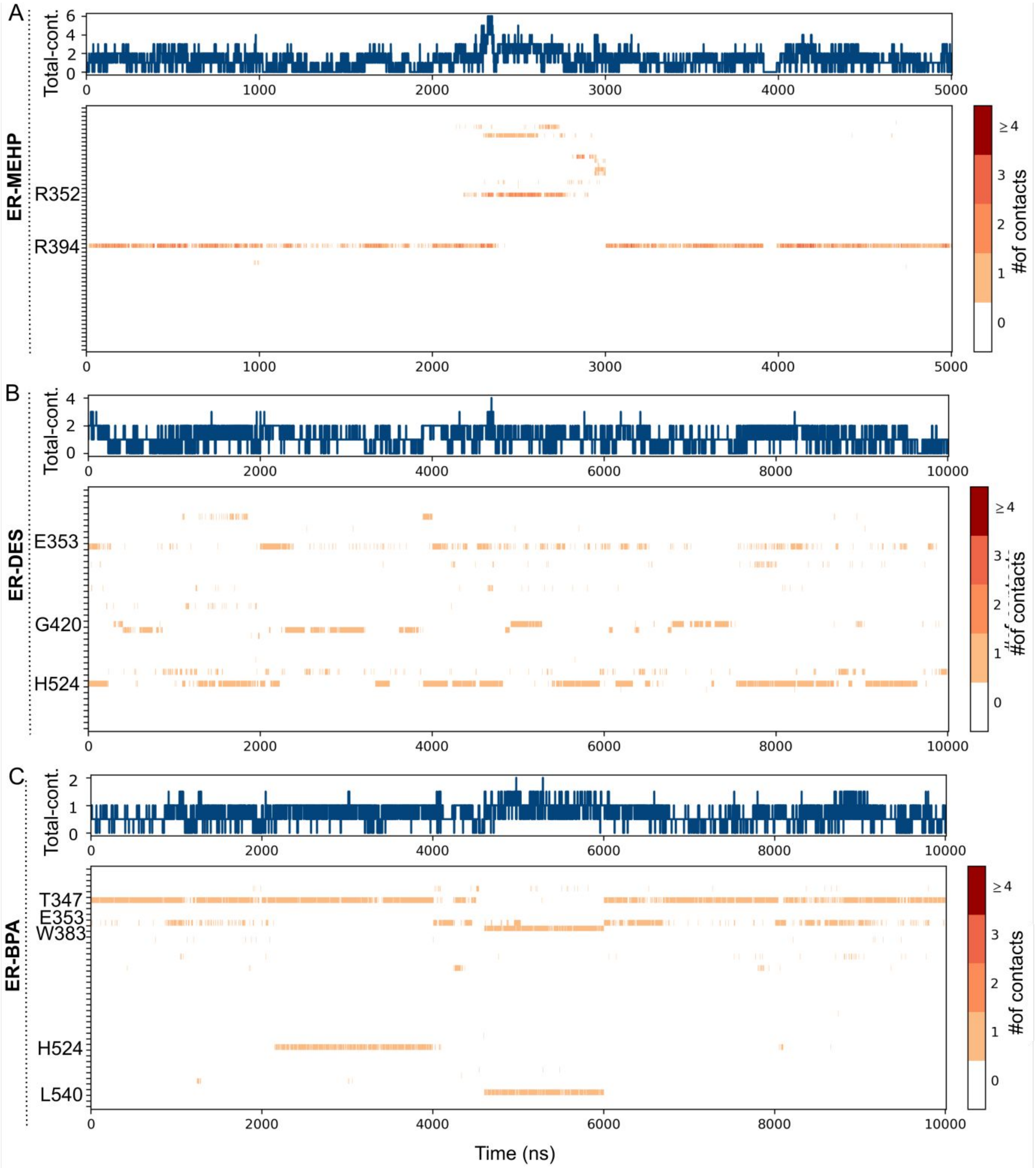

Figure S12. Timeline visulization of the H-bond for compounds bound to ER. MEHP (A), DES (B), BPA (C). The top panel display the total number of contacts form between protein and ligand throughout the simulation time (graphs in blue). The bottom pannel highlight the residues that interact with the ligand at each trajectory frame. Residues that form multiple contacts with the ligand are indicated by darker shades of orange (shown on the scale to the right of plot).

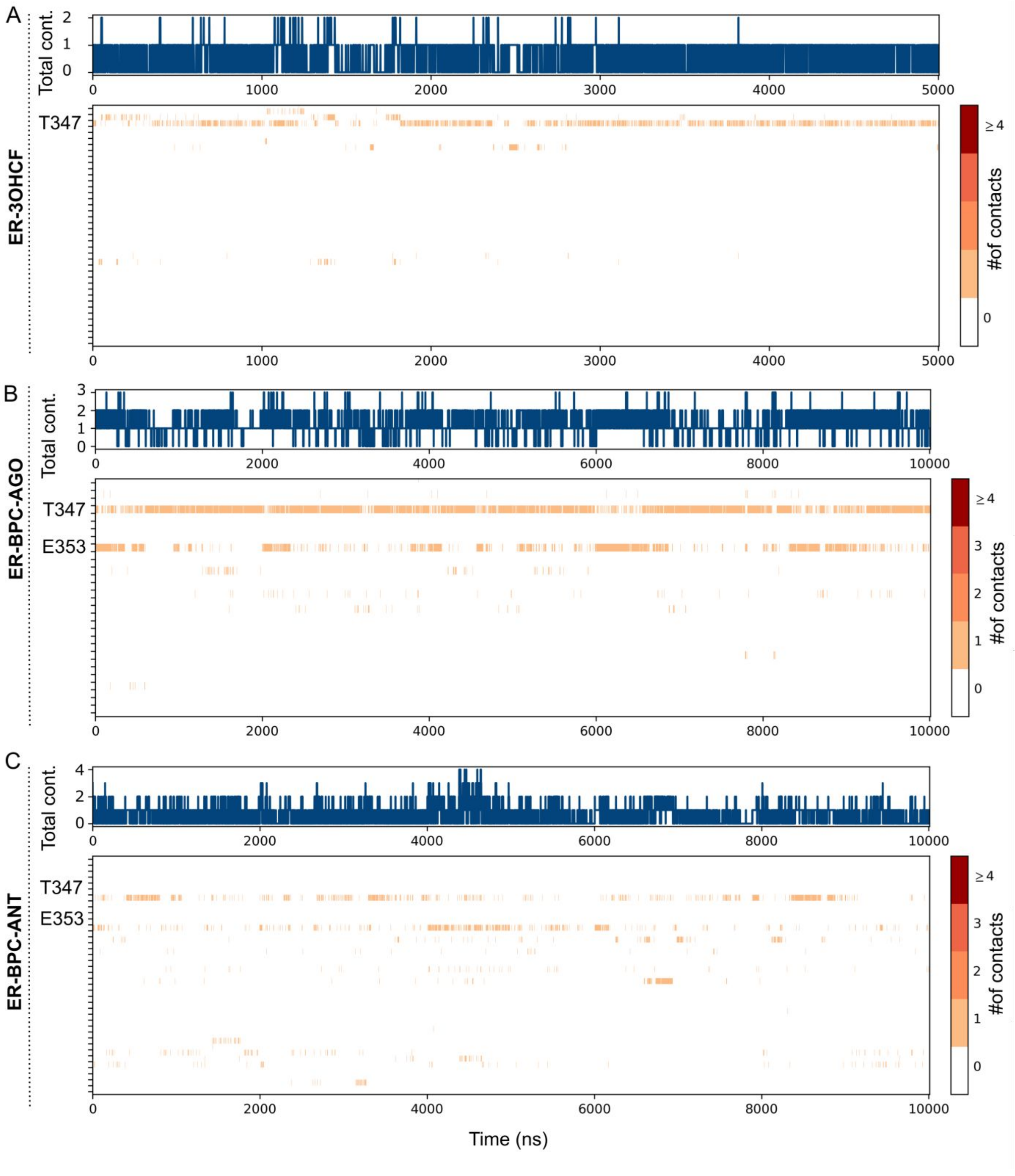

Figure S13. Timeline visulization of H-bonds for compounds bound to ER. 3OH-CF (A). BPC_{AGO} (B). BPC_{ANT} (C). The top panel display the total number of contacts form between protein and ligand throughout the simulation time (graphs in blue). The bottom pannel highlight the residues that interact with the ligand at each trajectory frame. Residues that form multiple contacts with the ligand are indicated by darker shades of orange (shown on the scale to the right of plot).

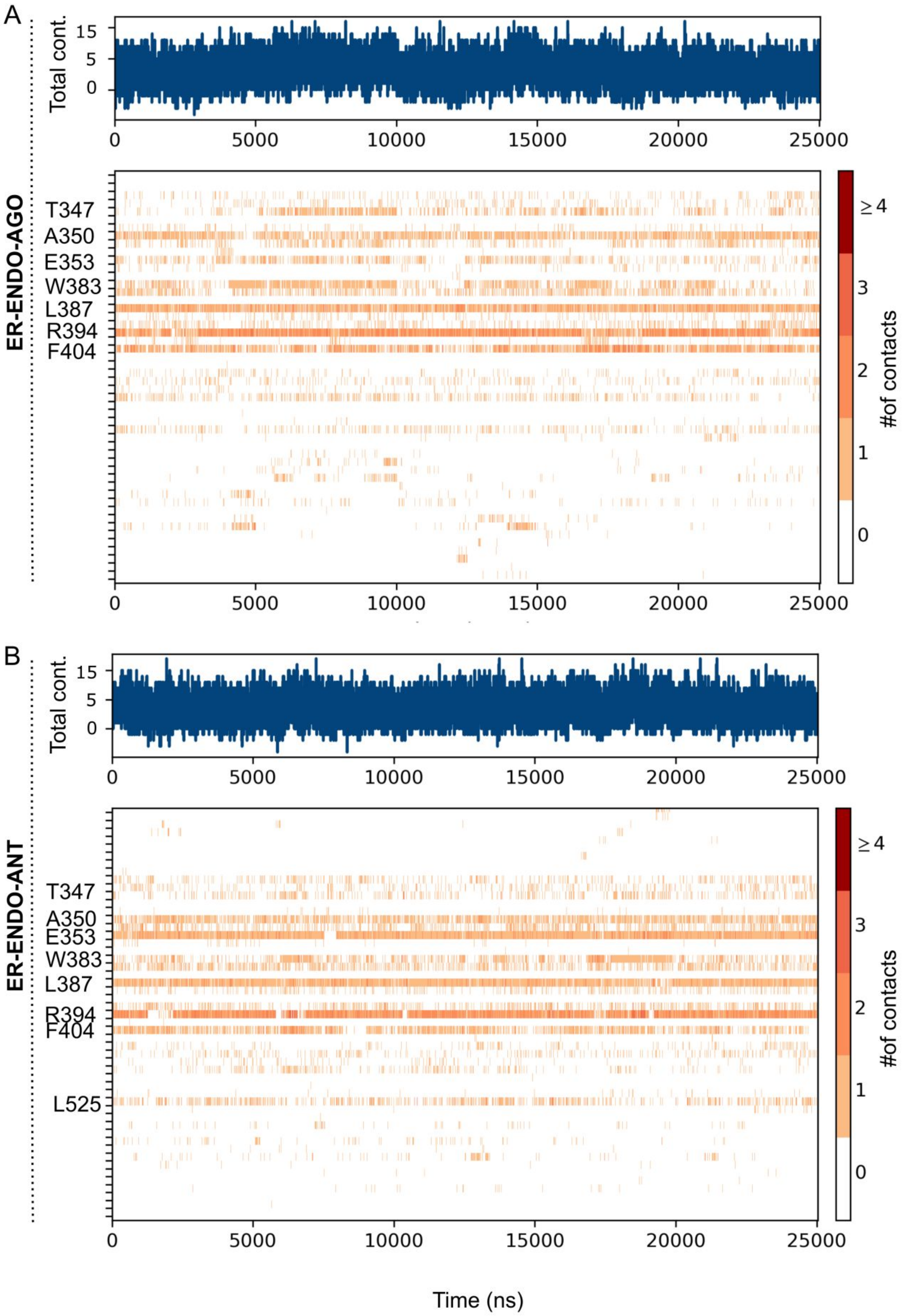

Figure S14. Timeline visulization of the H-bond for compounds bound to ER. Endoxifen, ENDO$_{AGO}$ (A), Endoxiphen, ENDO$_{ANT}$ (B). AGO and ANT denotes for agonist and antagonist, respectively pointing to the agonist and antagonist receptor conformation. The top panel display the total number of contacts form between protein and ligand throughout the simulation time (graphs in blue). The bottom pannel highlight the residues that interact with the ligand at each trajectory frame. Residues that form multiple contacts with the ligand are indicated by darker shades of orange (shown on the scale to the right of plot).

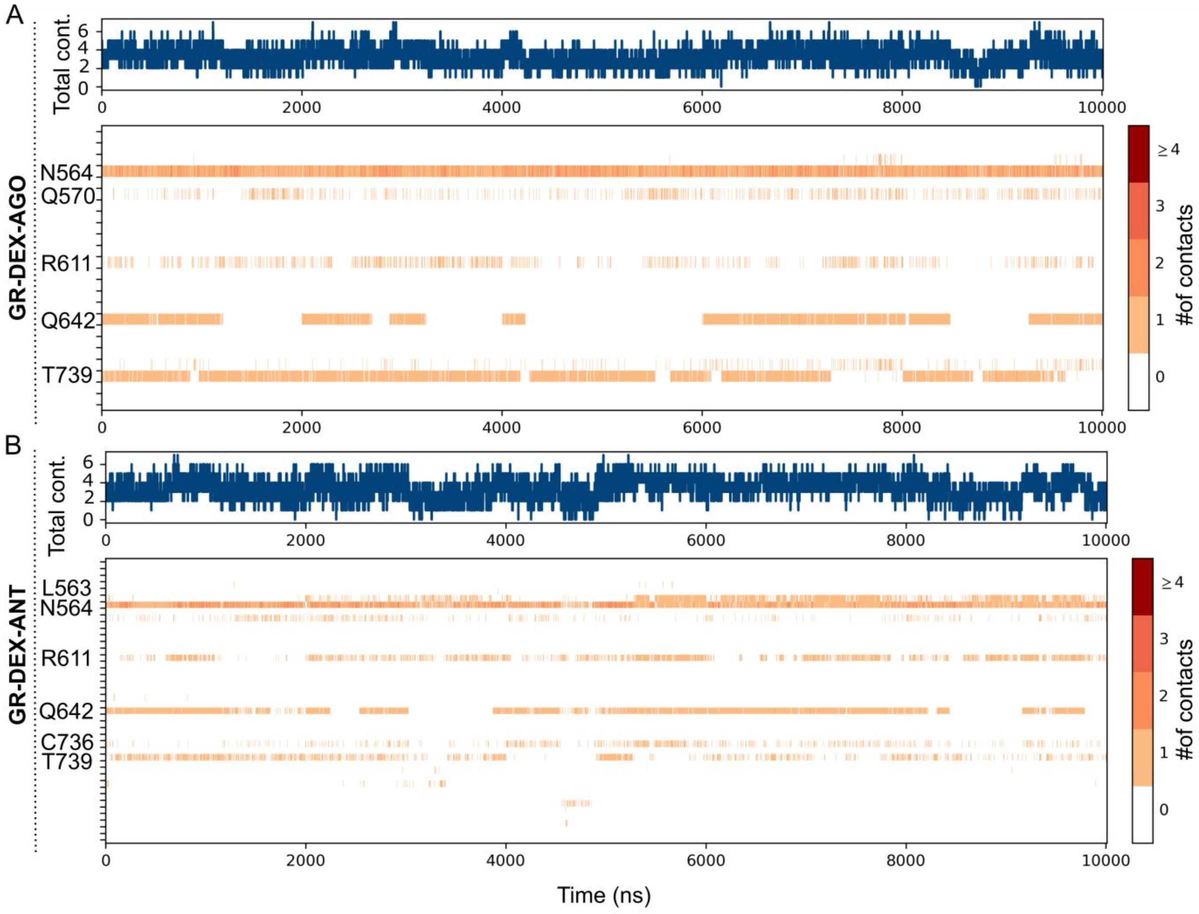

Figure S15. Timeline visulization of the H-bond for compounds bound to GR. GR-DEX$_{AGO}$ (A). GR-DEX$_{ANT}$ (B). The top panel display the total number of contacts form between protein and ligand throughout the simulation time (graphs in blue). The bottom pannel highlight the residues that interact with the ligand at each trajectory frame. Residues that form multiple contacts with the ligand are indicated by darker shades of orange (shown on the scale to the right of plot).

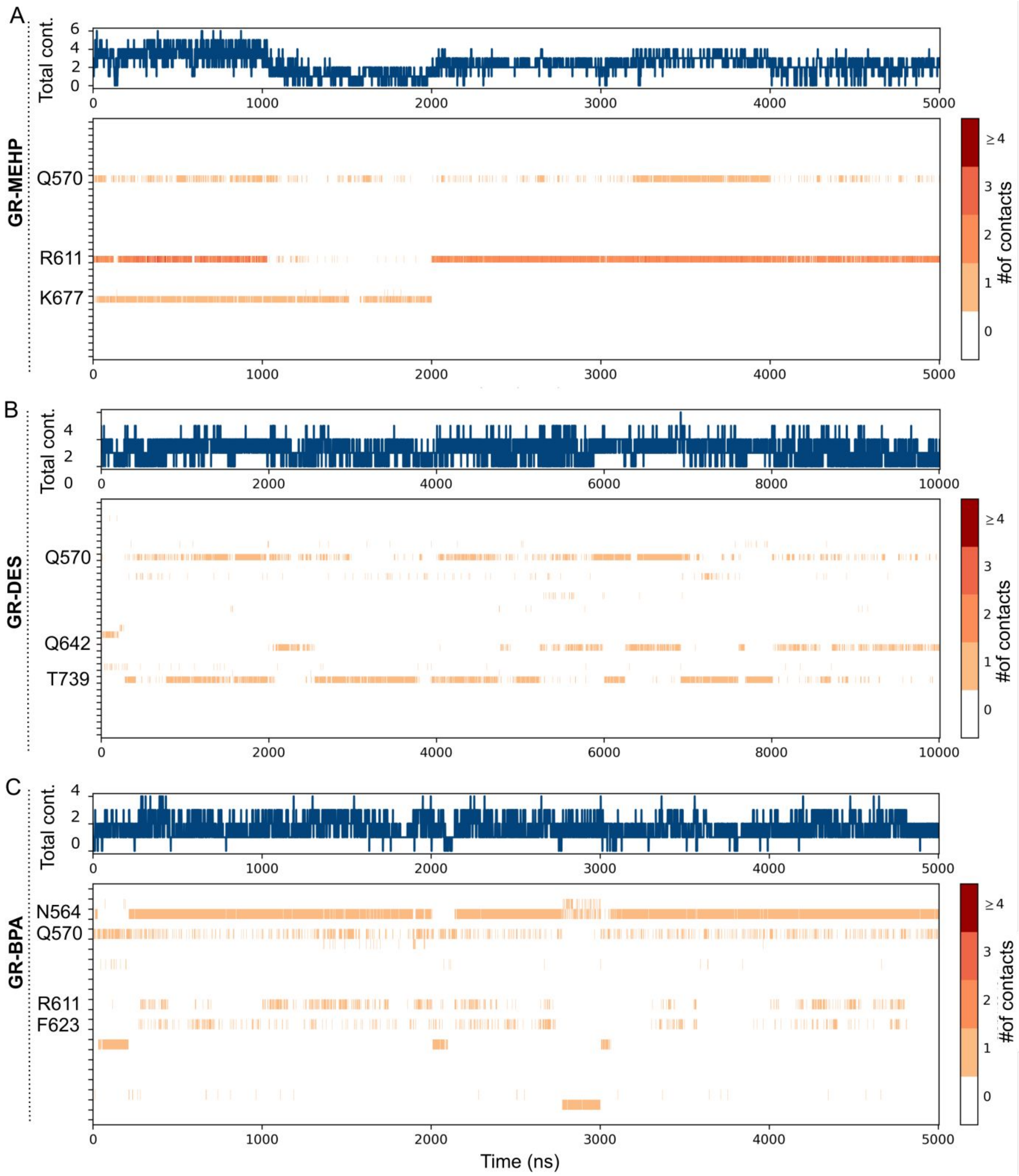

Figure S16. Timeline visulization of the H-bond for compounds bound to GR. MEHP (A). DES (B). BPA (C). The top panel display the total number of contacts form between protein and ligand throughout the simulation time (graphs in blue). The bottom pannel highlight the residues that interact with the ligand at each trajectory frame. Residues that form multiple contacts with the ligand are indicated by darker shades of orange (shown on the scale to the right of plot).

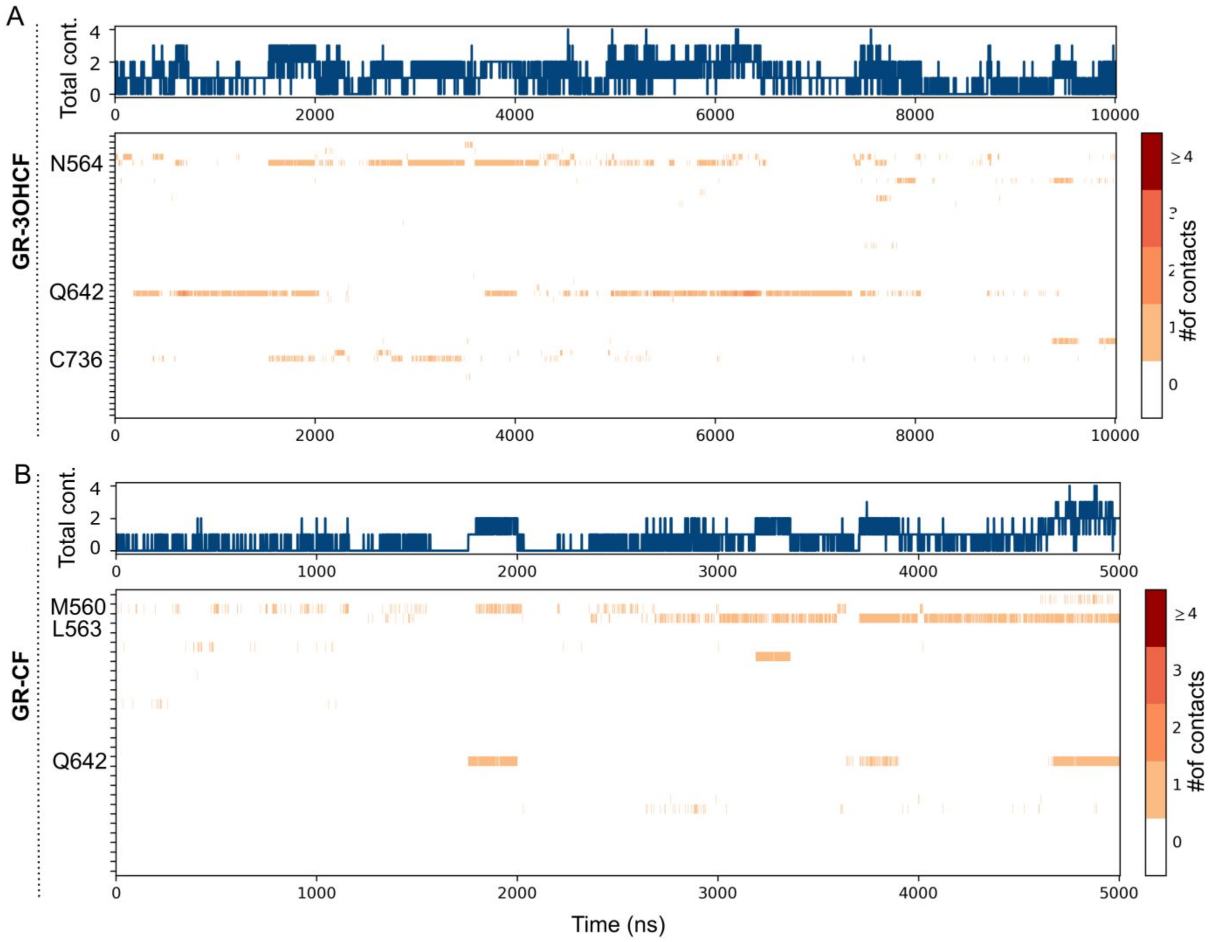

Figure S17. Timeline visulization of the H-bond for compounds bound to GR. 3OHCF (A) and CF (B). The top panel display the total number of contacts form between protein and ligand throughout the simulation time (graphs in blue). The bottom pannel highlight the residues that interact with the ligand at each trajectory frame. Residues that form multiple contacts with the ligand are indicated by darker shades of orange (shown on the scale to the right of plot).

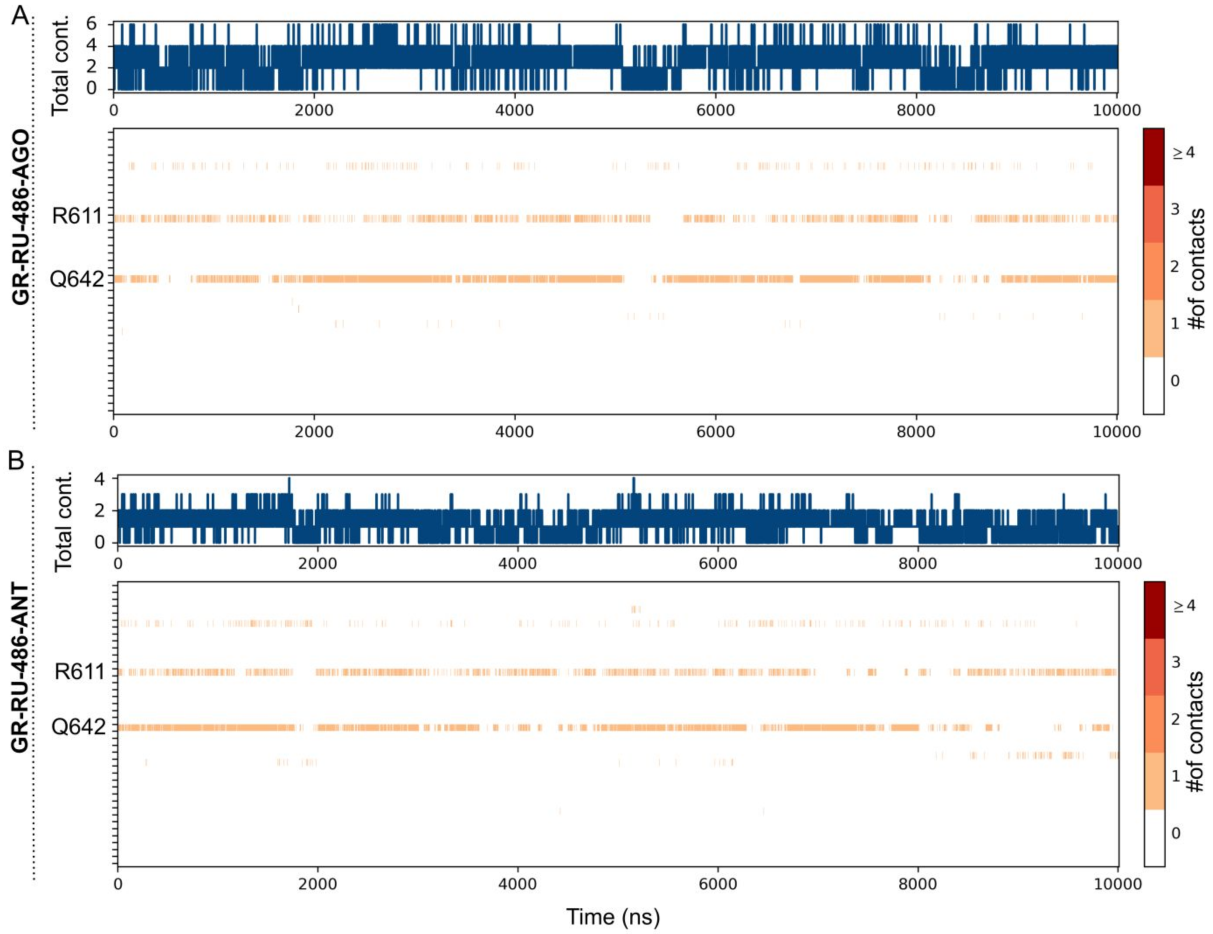

Figure S18. Timeline visulization of the H-bond for compounds bound to GR. RU-486$_{AGO}$ (A) and RU-486$_{ANT}$ (B). The top panel display the total number of contacts form between protein and ligand throughout the simulation time (graphs in blue). The bottom pannel highlight the residues that interact with the ligand at each trajectory frame. Residues that form multiple contacts with the ligand are indicated by darker shades of orange (shown on the scale to the right of plot).

Hel	Res.	DEX_{AGO}	DEX_{ANT}	(*RR*)DEHP	(*RS*)DEHP	(SS)DEHP	MEHP	DES	BPA	DDE	(RS)PROP	(RR)PROP	3OH-CF	CF	RU4-86_{AGO}	RU-486_{ANT}
H3	M560	7.9	6.0	1.0	10.2	24.0	0.5	8.7	4.8	7.0	6.6	0.9	5.9	3.5	0.6	0.6
H3	L563		38.0													
H3	L563	5.6	3.5	10.0	13.2	17.2	5.0	12.2	15.3	23.0	19.7	11.0	22.1	32.8	16.5	17.9
H3	N564	96.0	54.0						86.0		30.0	10.0				14.0
H3	L566	1.1	0.8	25.8	7.4	6.1	2.9	0.7	4.5	0.4	4.4	3.3	2.6	7.0	1.7	1.8
H3	Q570						21.0	39.0	17.0					15.0		
H4	W600					17.0						18.0				
H4	W600	6.3	6.8	1.2	6.0	9.5	16.8	2.3	4.9	0.7	1.8	5.7	3.3	8.2	16.1	6.9
H4	M601	7.5	3.8	5.4	9.6	9.3	0.2	12.9	19.6	11.1	8.3	6.9	9.2	15.6	8.1	3.2
H4	L603						10.0									
H5	M604	18.9	27.1	15.4	4.8	9.9	38.3	31.2	22.8	13.9	15.6	16.6	14.2	13.8	19.7	22.0
H5	L608	2.5	11.2	10.3	16.4	13.3	6.0	18.8	13.3	7.6	8.6	13.7	10.8	16.4	12.5	12.8
H5	R611	36.0	46.0						18.0			12.0		11.0	46.0	45.0
βa	F623			56.0			73.0		28.0	33.0		14.0			11.0	11.0
βa	F623	12.2	3.7	16.3	13.8	13.2	6.7	4.7	6.0	6.4	9.1	7.4	11.9	9.5	22.8	16.8
βa	M639	0.3	0.4	2.7	5.0	6.9	1.3	11.9	0.4	3.1	7.5	4.7	5.3	1.8	4.8	6.2
H7	Q642	53.0	68.0								11.0				72.0	57.0
H7	M646	2.0	2.1	5.2	6.7	6.3	1.8	12.5	4.0	8.2	10.7	4.7	10.8	10.8	7.2	7.1
H8	K667						17.0									
H10	T739	77.0	33.0					47.0								
H11	L732	3.9	5.9	2.3	13.2	13.1	2.0	4.5	5.4	10.9	14.2	9.4	8.0	9.0	18.2	14.5
H11	Y735	10.9	13.6	5.1	12.4	14.6	1.8	17.5	1.2	6.3	9.6	1.3	7.9	4.6	0.3	0.4
H11	C736	1.3	1.8	0.1	11.1	4.4	0.0	1.9	0.0	0.7	1.0	1.7	1.4	0.3	1.6	0.0
H11	F749	0.2	0.1	1.2	23.7	11.0	1.4	0.6	0.0	0.7	3.4	6.9	1.2	7.8	25.4	9.7

Table S3. Protein-ligand interaction in the GR. The two columns on the left refer to the GR helices (GR-Hel) and the corresponding amino acids involved in interactions, respectively. Yellow rows are hydrophobic contacts and polar contacts (water bridges and H bonds) are blue.

Hel	Res	E2AGO	E2ANT	(*RR*)DEHP	(*RS*)DEHP	(SS)DEHP	MEHP	DES	BPA	DDE	(RS)PROP	(RR)PROP	3OH-CF	CF	BPC$_{AGO}$	BPC$_{ANT}$	ENDO$_{AGO}$	ENDO$_{ANT}$
H3	M343	1.9	0.8	3.0	6.7	4.3	0.3	5.0	5.6	2.1	0.9	1.4	4.3	2.9	6.1	5.5	19.22	11.4
H3	L346	0.9	3.5	32.1	20.2	23.5	16.8	11.9	26.6	8.7	15.0	21.0	17.5	23.8	19.4	18.6	14.95	11.1
H3	T347								70.0				45.0	15.0	81.0		0	16
H3	L349	4.5	6.4	9.5	6.9	8.7	22.3	3.1	1.2	6.9	4.7	8.7	2.1	3.2	2.4	2.8	1.12	1.5
H3	A350	1.3	3.9	20.1	29.2	26.4	7.7	20.1	29.7	42.2	7.6	9.9	17.8	9.9	38.8	58.6	65.8	71.7
H3	E353	38.0	31.0				44.0		28.0		22.0	33.0			33.0	53.0	94	37
H4	W383								13.0				19.0	15.0			16	29
H4	L384	2.9	12.3	9.4	13.4	15.6	3.7	8.5	13.3	20.6	4.7	9.6	4.3	10.6	6.8	12.3	40.1	39.8
H4	L387	41.0	32.0				23.0		13.0			14.0			16.0		88	82
H4	L387	21.4	19.9	16.6	14.5	19.3	24.6	10.0	16.5	21.5	24.3	25.5	14.9	13.3	18.0	27.3	15.5	25.7
H4	M388	6.6	9.1	9.8	11.1	9.6	5.4	12.8	4.4	1.0	6.7	6.2	3.2	11.2	0.3	1.2	16.8	11.4
H5	L391	10.2	10.4	11.4	11.5	13.1	6.7	41.1	16.1	21.0	14.7	21.3	10.5	9.1	17.9	17.6	29.6	24.1
H5	R394	14.0					47.0					14.0					79	70
βa	F404	65.0	50.0				22.0		28.0	46.0		16.0			42.0	51.0	45	47
βa	F404	5.1	6.6	15.2	21.6	16.2	16.9	11.3	2.2	0.9	9.8	9.6	8.2	5.5	8.2	3.4	5.04	4.8
βa	M421	1.5	3.1	8.3	8.9	8.5	3.9	14.8	4.4	0.4	5.3	6.9	4.6	9.9	3.1	0.7	9.5	6.3
H7	I424	11.4	3.6	8.2	6.9	7.8	2.4	10.9	2.3	0.0	5.3	0.6	1.8	7.9	0.0	0.0	14.4	15.1
H7	F425	0.0	0.4	9.8	12.1	7.3	3.5	4.8	1.4	0.0	1.9	2.5	0.7	7.5	0.1	0.0	5.2	4.5
H11	H524	90.0						45.0	15.0								0	0
H11	L525	2.0	5.6	21.1	29.5	30.0	4.0	17.8	24.7	26.0	31.8	18.4	15.7	17.5	19.1	19.9	37.6	20.8
H12	L540								12.0								0	0

Table S4. ERα protein-ligand interactions. The two columns on the left refer to the ERα helices (ER-Hel) and the corresponding amino acids involved in interactions, respectively. Yellow rows are hydrophobic contacts and polar contacts (water bridges and H bonds) are blue.

Table S5. Predicted binding energy for the EDCs in the ERα simulations. Ligand efficiency normalized by heavy atoms count (LN) scales the ligand efficiency to better fit the experimental data for maximal affinity of ligands[1], TOTAL represents the cumulative average followed by average of each simulation replica (#1 - #5) Hbond, lipophilic and Coulombic represents the binding energy (kcal/mol) for those individual terms. The terms were calculated using MM/GBSA along the simulation trajectory, but only every 10th frame, the total number of analysed frames is stated. Heatmap for LN (red, white, blue – colors are autoscaled within each variable).

ERα	# Frames	Ligand Efficiency (LN)												Hbond		Lipophilic		Coulombic	
		Total		#1		#2		#3		#4		#5		Total		Total		Total	
		Mean	SD	Mean	SD	Mean	SD	Mean	SD	Mean	SD	Mean	SD	Mean	SD	Mean	SD	Mean	SD
17β-E2$_{AGO}$	1000	-15.52	0.89	-15.64	0.90	-15.52	0.92	-15.48	0.84	-15.48	0.95	-15.60	0.94	-1.01	0.46	-27.69	1.63	-9.78	2.54
17β-E2$_{ANT}$	1000	-15.11	1.53	-15.22	1.49	-15.35	1.28	-16.05	1.63	-14.87	1.15	-14.09	1.10	-0.86	0.47	-27.21	2.44	-9.16	3.31
(*RR*)-DEHP	500	-19.65	1.28	-19.72	0.91	-19.31	1.23	-19.53	1.47	-19.53	1.13	-20.12	1.43	-0.02	0.01	-34.45	2.64	-1.71	0.95
(*RS*)-DEHP	500	-19.60	1.27	-20.53	1.21	-19.35	1.09	-19.37	1.05	-19.13	0.96	-19.07	1.18	-0.03	0.16	-33.77	2.39	-2.24	1.90
(SS)-DEHP	500	-19.37	1.14	-19.27	1.25	-19.93	1.03	-19.36	1.02	-19.34	1.21	-18.96	0.93	-0.02	0.12	-34.15	2.24	-2.01	1.84
MEHP	500	-12.01	1.91	-11.27	1.14	-10.86	0.87	-13.89	2.91	-11.95	0.97	-12.00	1.13	-1.51	0.85	-20.86	2.97	11.66	8.70
DES	1000	-15.11	1.35	-15.13	1.34	-14.93	1.36	-15.13	1.14	-15.19	1.46	-15.18	1.39	-0.74	0.48	-27.60	1.94	-10.90	4.08
BPA	1000	-14.44	1.27	-14.13	0.96	-15.46	1.09	-14.76	1.47	-13.93	0.91	-13.91	1.10	-0.99	0.29	-24.45	1.59	-13.71	1.87
DDE	1000	-17.55	0.99	-17.81	0.88	-17.31	0.98	-17.50	1.02	-17.77	0.92	-17.35	1.04	0.00	0.00	-31.74	1.77	-2.26	1.02
(*RS*)-PROP	1000	-16.43	1.15	-17.14	1.17	-17.25	1.15	-15.83	0.92	-16.04	0.92	-15.93	1.07	-0.12	0.19	-28.16	1.55	-4.31	2.80
(*RR*)-PROP	1000	-15.69	0.80	-15.84	0.90	-15.89	0.90	-15.19	1.42	-15.46	1.11	-16.06	1.12	-0.03	0.02	-27.15	1.67	-3.35	1.59
3OH-CF	1000	-12.15	0.87	-12.42	1.00	-12.00	0.91	-12.09	0.83	-12.00	0.70	-12.23	0.75	-0.33	0.22	-17.99	1.38	-5.68	2.79
CF	500	-12.37	1.05	-12.94	1.30	-12.57	0.94	-11.99	1.07	-11.96	0.74	-12.38	0.93	-0.23	0.25	-17.33	1.24	-5.33	3.32
BPC$_{AGO}$	1000	-15.48	1.06	-15.37	1.07	-15.31	1.12	-15.62	1.01	-15.55	1.09	-15.53	0.98	-0.46	0.22	-26.43	1.70	-13.36	3.63
BPC$_{ANT}$	1000	-21.14	1.16	-14.06	1.23	-14.02	1.34	-13.37	1.24	-14.84	1.68	-14.54	1.32	-0.60	0.40	-26.33	1.96	-8.03	3.76
ENDO$_{AGO}$	500	-13.73	1.18	-13.39	1.18	-13.93	1.08	-13.83	1.26	-13.74	1.00	-13.82	1.17	-0.72	0.50	-32.99	2.14	-34.67	6.61
ENDO$_{ANT}$	500	-14.94	1.17	-14.66	1.17	-15.15	1.15	-14.90	1.13	-15.33	1.24	-14.66	1.38	-1.58	0.40	-32.06	2.17	-48.10	5.97

[1] Kuntz *et al*., (1999). The maximal affinity of ligands. https://doi.org/10.1073/pnas.96.18.9997

Table S6. Predicted binding energy for the EDCs in the GR simulations. Ligand efficiency normalized by heavy atoms count (LN) scales the ligand efficiency to better fit the experimental data for maximal affinity of ligands[1], TOTAL represents the cumulative average followed by average of each simulation replica (#1 - #5) Hbond, lipophilic and Coulombic represents the binding energy (kcal/mol) for those individual terms. The terms were calculated using MM/GBSA along the simulation trajectory, but only every 10th frame, the total number of analysed frames is stated. Heatmap for LN (red, white, blue – colors are autoscaled within each variable).

		Ligand Efficiency (LN)												Hbond		Lipophilic		Coulombic	
GR	# Frames	Total		#1		#2		#3		#4		#5		Total		Total		Total	
		Mean	SD	Mean	SD	Mean	SD	Mean	SD	Mean	SD	Mean	SD	Mean	SD	Mean	SD	Mean	SD
DEX_{AGO}	1000	-20.06	1.18	-20.02	1.03	-19.85	1.02	-19.60	1.00	-19.99	1.30	-19.81	1.10	-1.80	0.35	-26.81	1.42	-25.33	3.28
DEX_{ANT}	1000	-18.59	1.26	-18.76	1.24	-18.32	1.56	-18.54	1.43	-18.90	1.00	-18.43	1.24	-1.97	0.57	-24.72	1.69	-24.6	4.79
(*RR*)-DEHP	500	-19.94	1.01	-19.39	1.00	-19.87	0.98	-20.27	0.92	-19.93	1.00	-20.27	0.86	-0.01	0.06	-32.01	1.55	-5.56	1.12
(*RS*)-DEHP	500	-20.27	1.35	-19.72	1.24	-20.15	1.21	-20.67	1.21	-19.96	1.42	-20.84	1.29	-0.06	0.01	-33.23	2.47	-4.35	1.37
(SS)-DEHP	500	-20.37	1.48	-19.81	1.21	-20.36	1.63	-19.86	1.53	-20.57	1.24	-21.26	1.27	-0.04	0.09	-33.68	2.34	-3.79	1.93
MEHP	500	-14.09	1.03	-14.29	0.96	-13.53	0.93	-14.08	0.87	-14.68	0.94	-13.90	1.06	-2.52	0.85	-20.68	1.43	-18.73	8.14
DES	1000	-15.87	1.58	-16.09	1.68	-15.77	1.54	-15.81	1.63	-16.39	1.40	-15.29	1.43	-0.89	0.37	-25.78	2.25	-15.79	4.94
BPA	500	-15.27	1.28	-15.35	1.25	-15.07	1.20	-15.38	1.38	-15.09	1.21	-15.45	1.29	-1.14	0.38	-24.29	1.72	-18.83	4.55
DDE	1000	-17.06	1.10	-16.82	1.03	-16.84	1.12	-17.41	1.00	-17.21	0.96	-17.02	1.26	0.00	0.00	-29.02	2.04	-2.96	1.26
(*RS*)-PROP	500	-13.01	0.99	-12.11	0.99	-12.91	1.46	-11.92	1.13	-13.50	1.27	-14.59	2.01	-0.38	0.22	-17.18	1.10	-8.48	2.45
(*RR*)-PROP	500	-15.95	0.89	-15.89	0.89	-16.24	1.03	-15.69	1.10	-15.73	0.98	-16.21	1.02	-0.05	0.18	-24.20	1.27	-6.46	1.81
3OH-CF	500	-13.63	1.57	-14.18	1.59	-14.16	1.25	-13.68	1.70	-13.28	1.53	-12.87	1.30	-0.60	0.34	-17.25	1.70	-13.77	4.03
CF	500	-16.48	1.29	-16.33	1.29	-16.73	0.86	-16.53	1.05	-16.10	1.12	-16.72	1.10	-0.11	0.20	-26.34	1.42	-6.95	2.17
RU-486_{AGO}	1000	-21.14	1.16	-21.33	1.37	-21.45	1.12	-20.93	1.08	-21.05	0.96	-20.97	1.14	-0.81	0.34	-36.82	1.92	-13.86	2.87
RU-486_{ANT}	1000	-20.66	1.29	-19.96	1.11	-20.90	1.18	-21.14	1.18	-20.90	1.40	-20.42	1.23	-0.77	0.30	-35.50	2.47	-13.39	3.15

[1] Kuntz *et al*., (1999). The maximal affinity of ligands. https://doi.org/10.1073/pnas.96.18.9997

Table S7. Coulombic energy terms of the predicted binding energy (kcal/mol) for individual replicas (#1 - #5) for relevant systems of ERα and GR simulations where the standard deviation (SD) was deviant. The terms were calculated using MM/GBSA along the simulation trajectory, but only every 10th frame. Heatmap for LN (red, white, blue – colors are autoscaled within each variable). Replicas with fonts in red are highlighted as a warning.

	**ERα**				**GR**	
Charge	MEHP	DES	ENDO-ant	ENDO-ago	GR-MEHP	GR-DES
AVG	11.7	-10.9	-48.1	-34.7	-18.7	-15.8
SD	8.7	4.1	6.0	6.6	8.1	4.9
AVG1	14.1	-11.1	-46.1	-33.4	-24.7	-17.0
SD1	8.7	4.6	6.0	6.6	8.1	5.4
AVG2	**22.0**	-10.7	-47.4	-33.5	-8.3	-13.8
SD2	**11.2**	3.7	5.9	5.2	7.4	5.0
AVG3	**-4.8**	-12.0	-49.8	-36.7	-20.4	-16.8
SD3	**16.5**	3.4	5.7	4.9	5.9	5.0
AVG4	13.0	-10.2	-48.7	-34.3	-24.0	-17.3
SD4	10.1	4.4	6.7	4.9	5.9	4.4
AVG5	14.5	-10.6	-48.5	-35.4	-16.4	-14.1
SD5	8.4	3.5	5.3	5.1	7.0	5.2

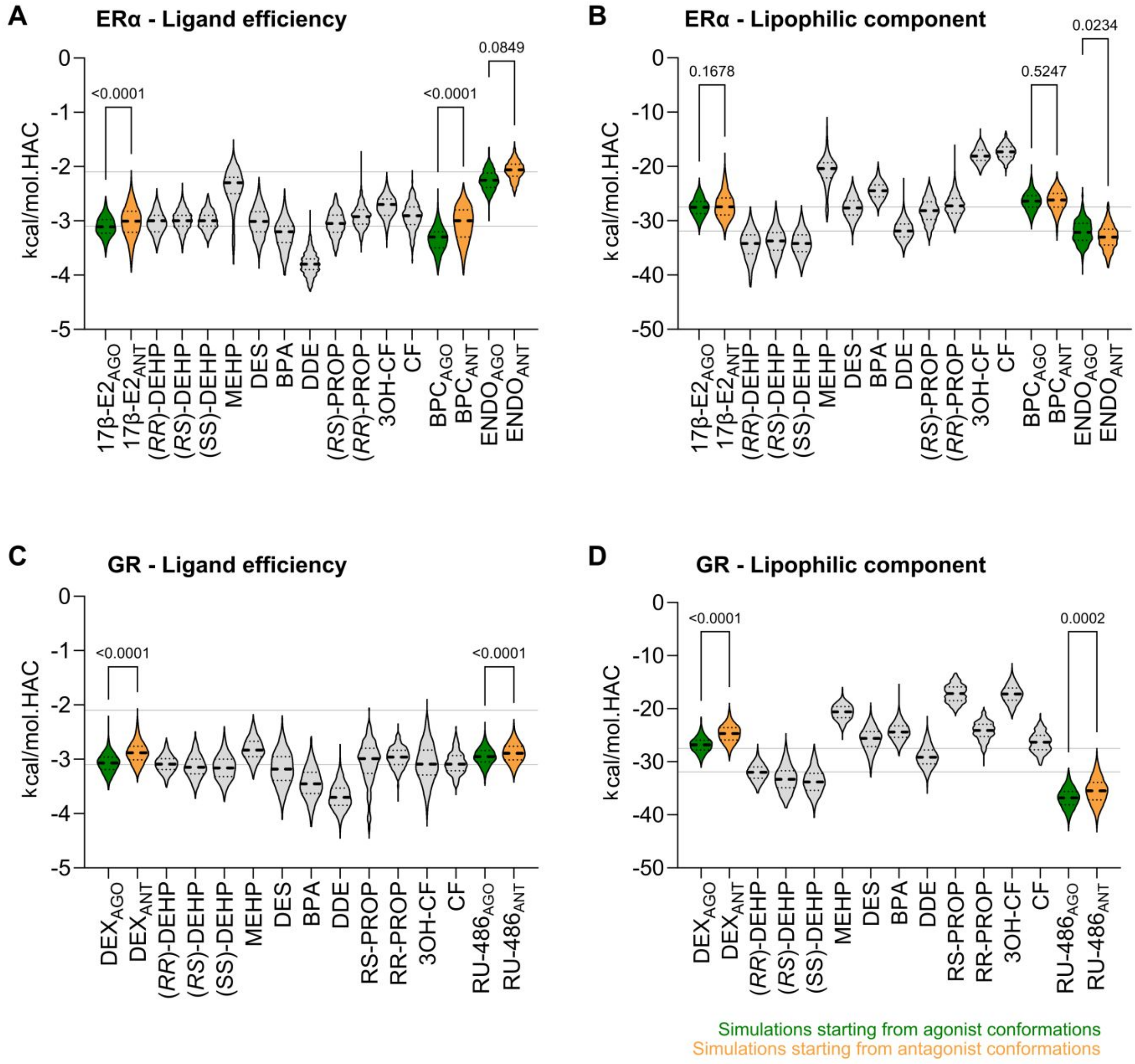

Figure S19. **Ligand efficiency prediction for the ERα and GR simulations displayed as violin plots**. Ligand efficiency (A,C) and specifically their lipophilic component (B,D) were calculated using MM/GBSA's predicted binding energy (see methods) for ERα (A,B) and GR (C,D). HAC: heavy atom count. Kruskal-Wallis H (KW) with Dunn's post hoc tests were performed to compare simulations starting from agonistic (green) and antagonistic conformations (orange) for each ligand, exact p-values are depicted when available as black numbers above the curves.

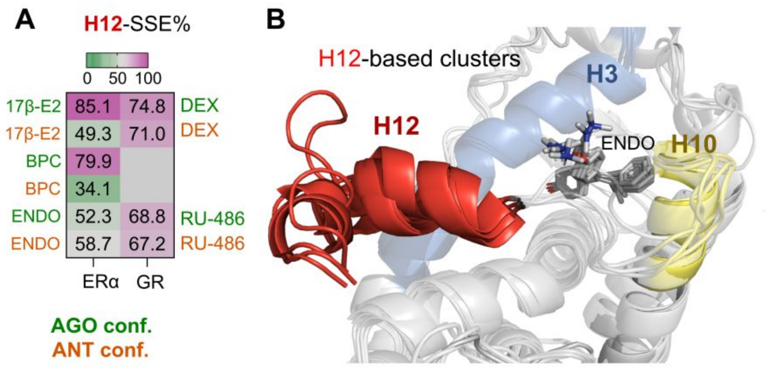

Figure 20. H12 folding plays an important role in the SHR activation. (A) The fraction of secondary structure elements (SSE%) in H12 appears more stable with agonistic compounds than with antagonists. (B) H12-based clusters of ERα conformation illustrating the H12 secondary structure. Structures were selected using hierarchical clustering based on the backbone's RMSD of H12.

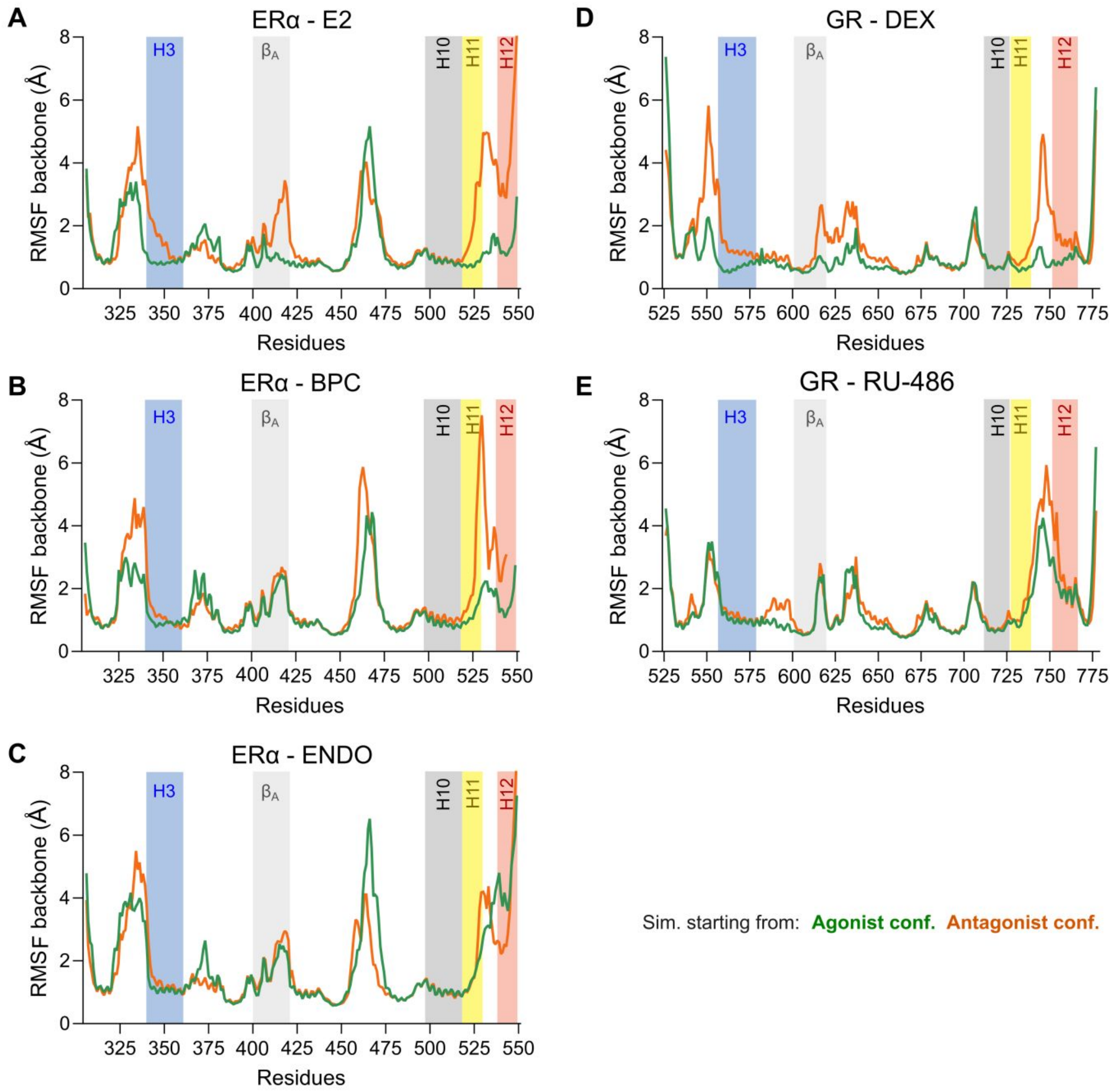

Figure S21. Root mean square fluctuation (RMSF) of the protein's backbone separated by residue and highlighted by secondary structure for ERα (A-C) and GR (D,E). Solid lines represent simulations starting from agonist (green) and antagonist (orange) conformations.

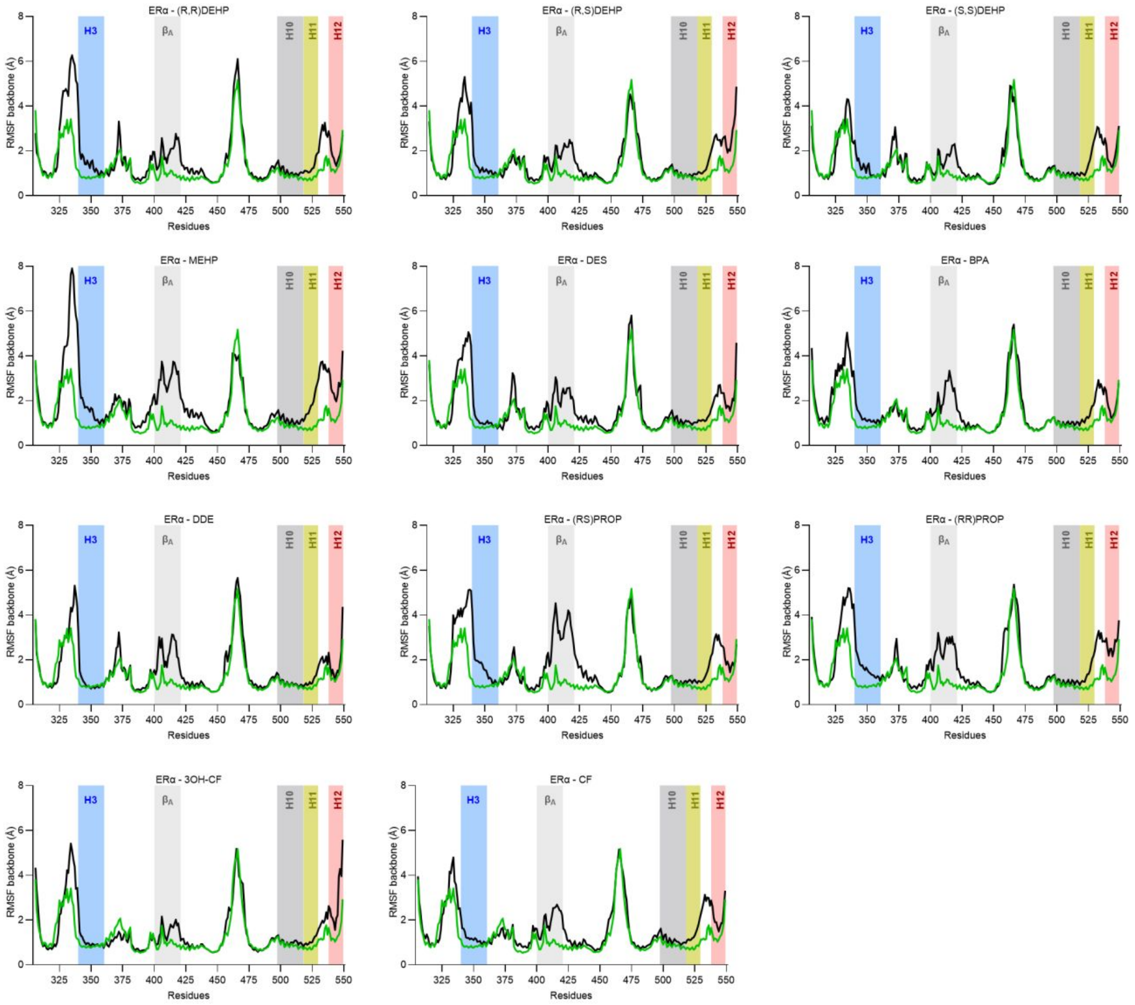

Figure S22. Root mean square fluctuation (RMSF) of the protein's backbone separated by residue and highlighted by helices for ERα with different EDC ligands. Solid lines represent simulations for standard E2 agonist (green) and respective EDC compound (black) starting from agonistic conformations.

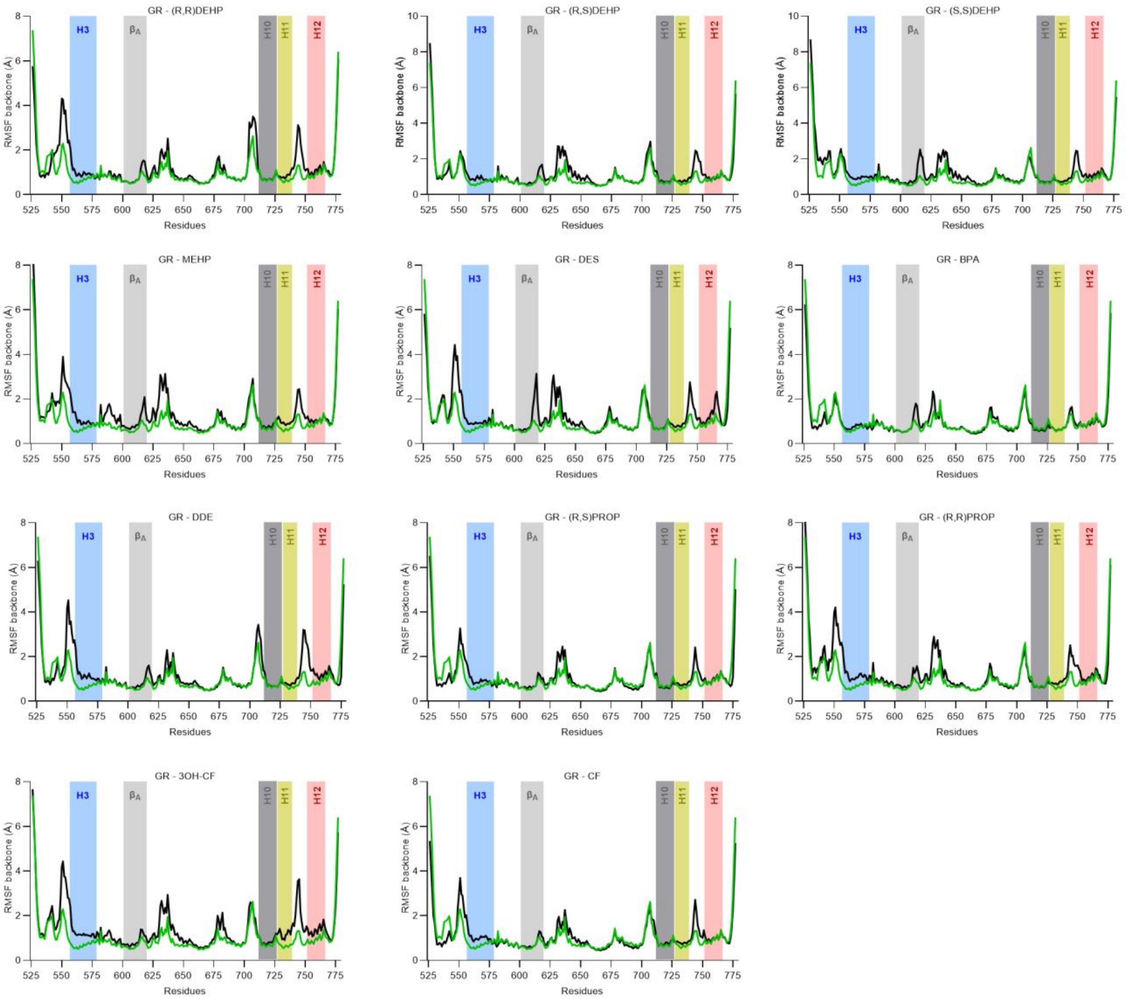

Figure S23. Root mean square fluctuation (RMSF) of the protein's backbone separated by residue and highlighted by helices for GR with different EDC ligands. Solid lines represent simulations for standard DEX agonist (green) and respective EDC compound (black) starting from agonistic conformations.

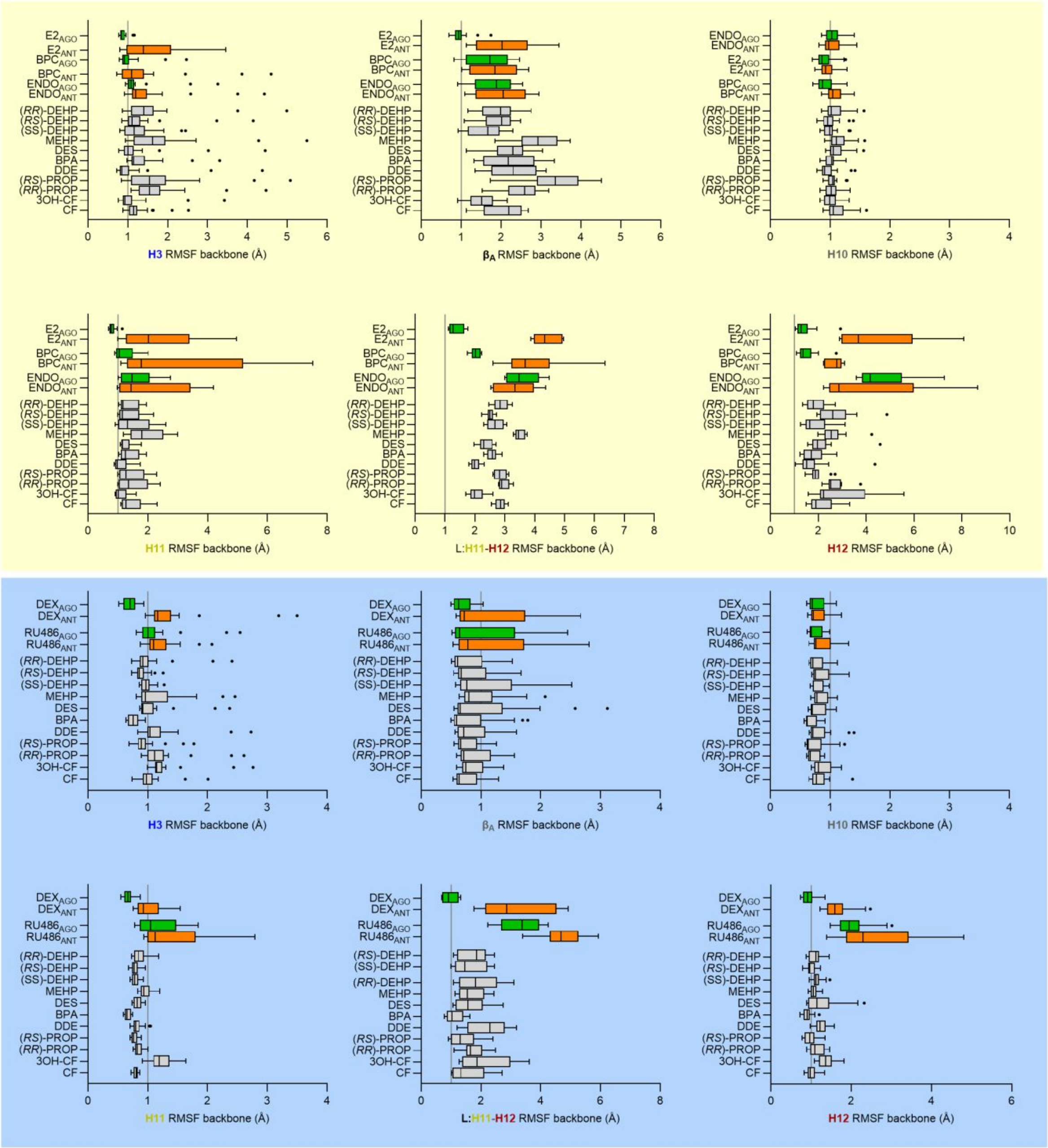

Figure S24. Protein's backbone root mean square fluctuation (RMSF) for specific helices. RMSF separated by residue and highlighted by helices H3 (blue labels), beta-sheet A (dark grey), H10 (light grey), H11 (yellow), L:H11-H12 and H12 (red) for ERα (upper graphics with yellow background) and GR (lower graphics with blue background). Box plots represent simulations starting from agonist (green) and antagonist (orange) conformations, outliers were determined using Tukey.

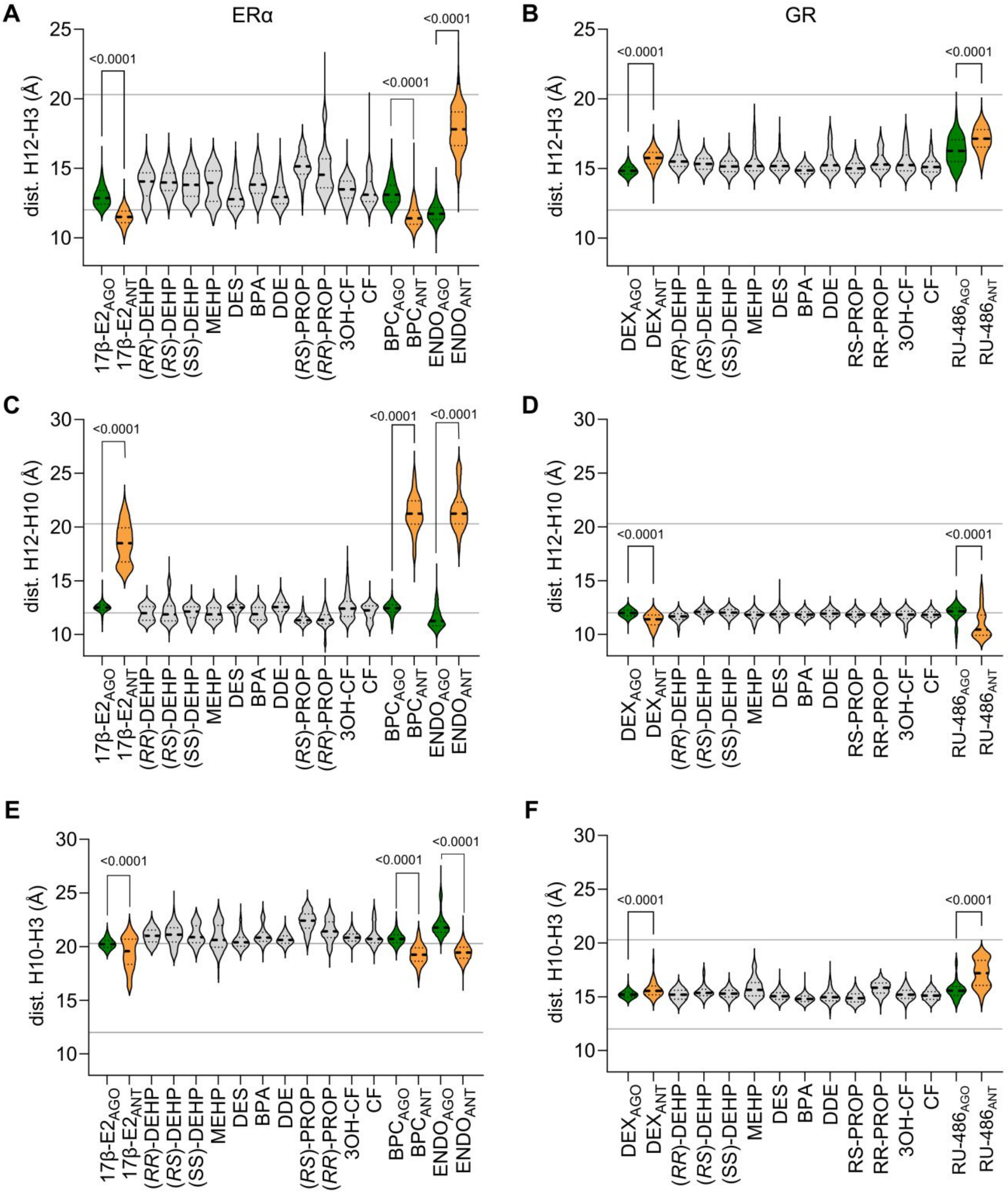

Figure S25. Distance plots between relevant helices are depicted as violin plots for the H12-H3 (A,B) and H12-H10 (C,D) and H10-H3 (E,F), for ERα (left) and GR (right) simulations. Kruskal-Wallis H (KW) with Dunn's post hoc tests were performed to compare simulations starting from agonistic (green) and antagonistic conformations (orange) for each ligand, exact p-values are depicted when available as black numbers above the curves.

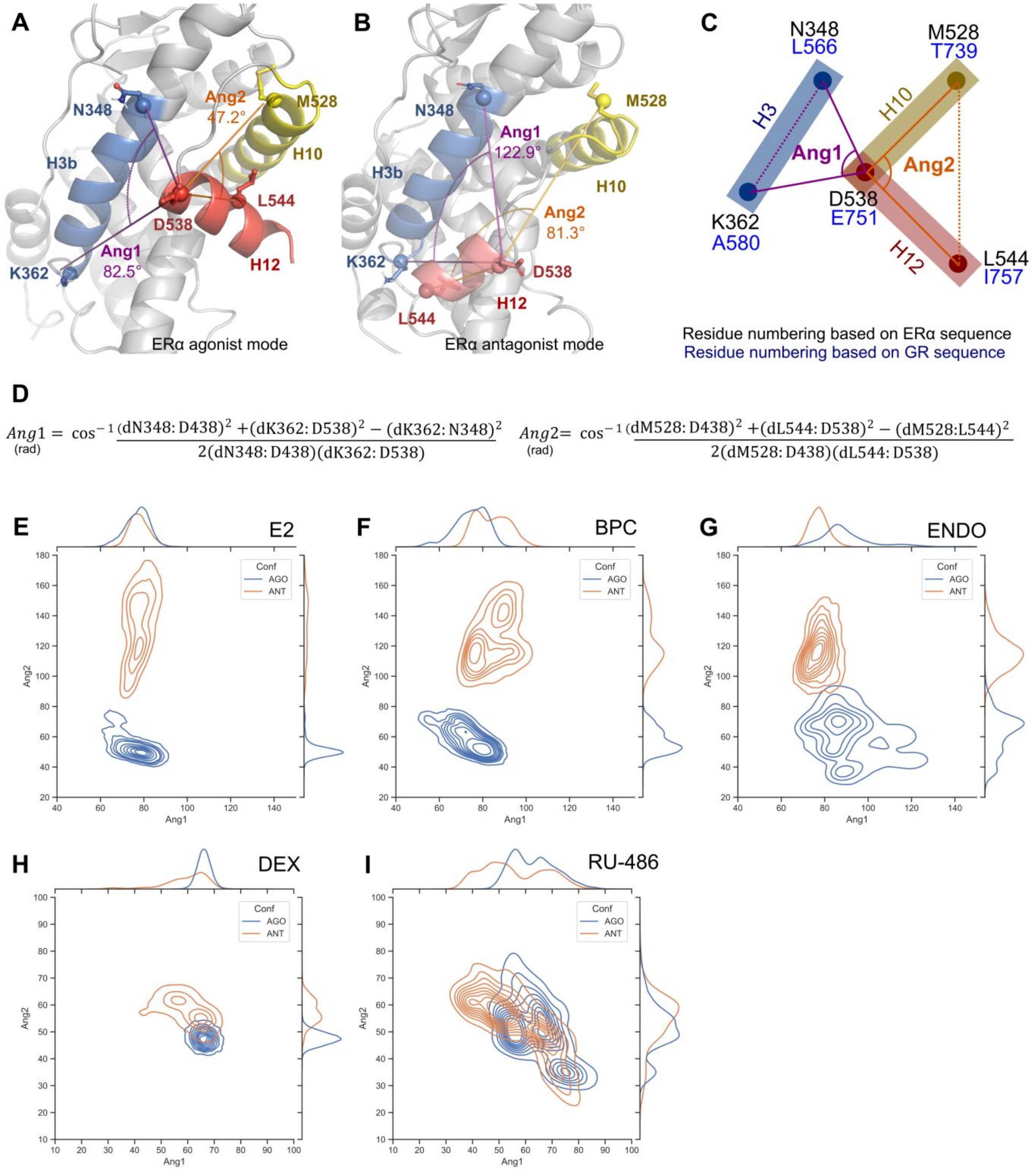

Figure S26. H12 relevant angle variation. Cartoon representation (A,B) and illustration (C) of angles 1 and 2 (Ang1,2) monitoring the conformational changes in the H12 highlighting the involved residues and distances used for the angle calculation (D, displays the relevant formula). Variation of the Ang1 vs Ang2 along the simulations of ERα bound to estradiol (E2, E), BPC (F) and endoxifen (ENDO, G) as well as GR's DEX (H) and RU-486 (I) simulations. In those angle distribution graphics blue represents simulations starting from agonist conformations while orange is starting from antagonist.

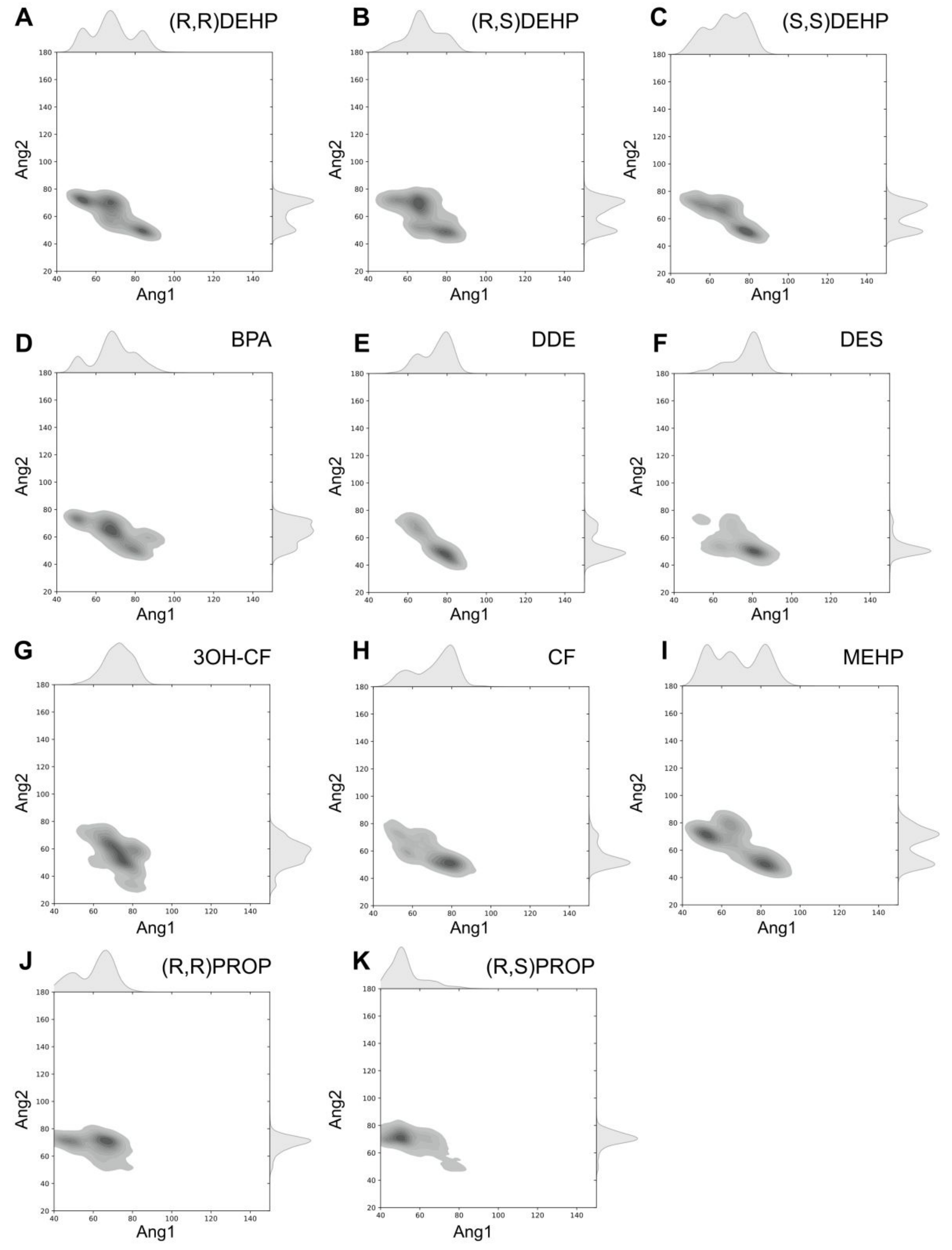

Figure S27. Variation of the Ang1 vs Ang2 along the simulations of ERα bound to different EDCs (A-K), namely: (R,R)DEHP (A), (R,S)DEHP (B), (S,S)DEHP (C), BPA (D), DDE (E), DES (F), 3OH-CF (G), CF (H), MEHP (I), (R,R)PROP (J) and (R,S)PROP (K).

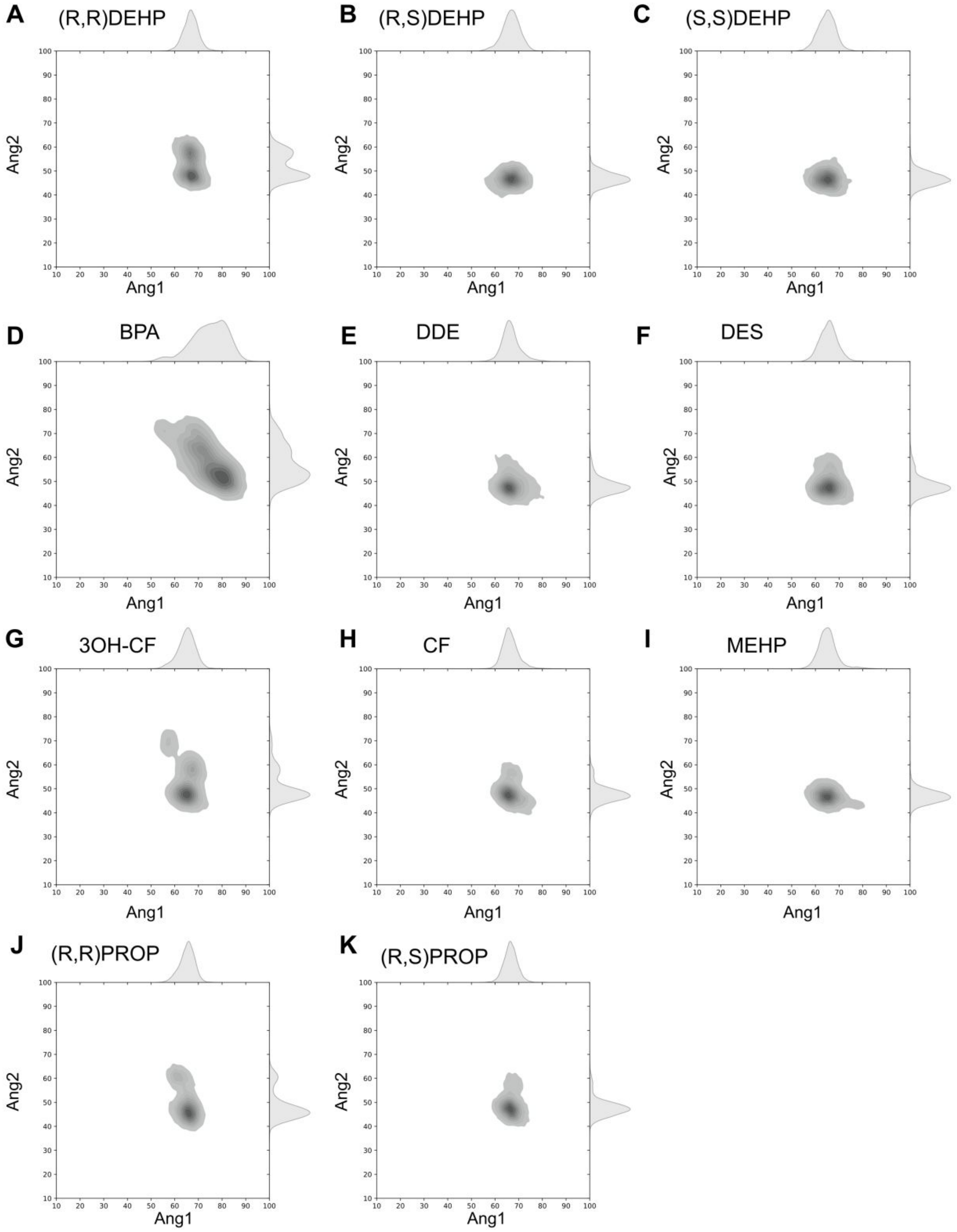

Figure S28. Variation of the Ang1 vs Ang2 along the simulations of GR bound to different EDCs (A-K), namely: (R,R)DEHP (A), (R,S)DEHP (B), (S,S)DEHP (C), BPA (D), DDE (E), DES (F), 3OH-CF (G), CF (H), MEHP (I), (R,R)PROP (J) and (R,S)PROP (K).

Table S8. Mean and standard deviation values for the pair-wise distances (Å) between the center of masses of helices H10, H3 and H12 for simulations, as well as the Ang1 and Ang2 defined in the methods section, of ERα and GR with different EDCs.

ERα	H10-H3		H12-H3		H12-H10		Ang1		Ang2	
	AVG	SD	AVG	SD	AVG	SD	AVG	SD	AVG	SD
17β-E2$_{AGO}$	20.3	0.5	13.0	0.7	12.5	0.4	76.90	5.59	51.1	5.3
17β-E2$_{ANT}$	19.4	1.5	11.5	0.6	18.5	1.9	77.95	4.63	133	21.1
(*RR*)-DEHP	21.0	0.7	13.9	1.1	12.0	0.7	68.16	10.89	63.2	9.7
(*RS*)-DEHP	21.1	1.0	14.0	0.9	12.1	1.1	68.19	9.25	61.9	10.4
(SS)-DEHP	21.1	1.0	13.8	1.0	12.0	0.7	68.64	10.09	61.8	9.5
MEHP	20.9	1.3	13.8	1.3	11.9	0.7	67.96	13.22	63.5	11.8
DES	20.5	0.8	13.0	1.0	12.4	0.6	76.35	8.52	53.1	7.1
BPA	21.1	0.9	13.9	1.0	11.9	0.7	69.25	10.68	63.0	8.6
DDE	20.7	0.5	13.1	0.9	12.6	0.7	74.40	7.92	54.0	9.9
(*RS*)-PROP	22.4	1.0	15.2	1.0	11.4	0.5	53.20	8.99	70.0	6.1
(*RR*)-PROP	21.5	1.0	14.9	1.7	11.4	0.8	60.63	9.89	69.1	6.3
3OH-CF	20.9	0.5	13.5	0.8	12.6	1.2	72.63	7.25	56.7	9.9
CF	21.0	1.0	13.4	1.1	12.1	0.8	71.40	10.62	56.4	8.8
BPC$_{AGO}$	20.8	0.6	13.2	0.9	12.4	0.6	74.60	7.59	57.5	7.8
BPC$_{ANT}$	19.3	0.9	11.5	0.8	21.3	1.8	84.35	7.90	122.9	16.4
ENDO$_{AGO}$	22.0	1.2	17.8	1.7	11.49	0.98	90.22	12.76	61.9	13.9
ENDO$_{ANT}$	19.4	0.8	11.77	0.7	21.51	1.86	77.20	4.45	115.5	11.9

GR	H10-H3		H12-H3		H12-H10		Ang1		Ang2	
	AVG	SD	AVG	SD	AVG	SD	AVG	SD	AVG	SD
DEX$_{AGO}$	15.2	0.4	14.9	0.4	12.0	0.4	66.20	2.62	47.6	2.4
DEX$_{ANT}$	15.7	0.8	15.7	0.6	11.3	0.7	59.47	8.48	57.4	4.7
(*RR*)-DEHP	15.2	0.6	15.6	0.7	11.6	0.5	66.83	3.19	52.2	5.4
(*RS*)-DEHP	15.5	0.6	15.3	0.5	12.1	0.4	66.75	3.79	46.7	2.9
(SS)-DEHP	15.3	0.5	15.2	0.5	12.0	0.4	64.83	3.78	46.7	3.0
MEHP	15.8	1.0	15.5	1.0	11.8	0.5	65.22	4.13	46.8	2.9
DES	15.1	0.5	15.3	0.6	11.9	0.5	65.69	3.95	48.4	4.2
BPA	14.8	0.4	14.9	0.4	11.9	0.4	65.83	2.95	47.9	2.5
DDE	15.0	0.6	15.5	0.9	11.9	0.4	66.73	4.02	48.1	3.8
(*RS*)-PROP	14.9	0.5	15.0	0.5	11.9	0.4	66.62	2.91	48.0	4.0
(*RR*)-PROP	15.8	0.6	15.5	0.8	11.9	0.4	65.20	3.16	48.5	6.0
3OH-CF	15.2	0.6	15.5	0.9	11.8	0.6	64.99	3.90	51.3	6.9
CF	15.1	0.5	15.2	0.6	11.9	0.4	66.49	3.47	48.0	4.0
RU-486$_{AGO}$	15.6	0.9	16.3	1.1	12.0	0.8	63.16	8.34	51.0	9.6
RU-486$_{ANT}$	17.2	1.3	17.1	0.9	11.0	1.4	55.75	11.93	54.2	9.9

Table S9. Ligand properties averaged along the simulation time.

		ERα					GR				
		**RMSD**	**rGyr**	**MolSA**	**SASA**	**PSA**	**RMSD**	**rGyr**	**MolSA**	**SASA**	**PSA**
17β-E2/DEXAGO	AVG	0.2	3.7	262.7	3.8	97.3	0.4	4.0	323.5	2.1	173.4
	SD	0.1	0.0	1.3	2.8	1.5	0.1	0.0	1.8	2.2	3.7
17β-E2/DEXANT	AVG	0.3	3.7	262.2	7.8	97.2	0.4	4.0	324.5	6.6	171.2
	SD	0.1	0.0	1.4	11.8	1.5	0.1	0.0	1.8	6.4	3.1
(*RR*)-DEHP	AVG	3.1	4.9	414.7	10.0	58.2	1.8	5.6	430.4	11.9	50.4
	SD	0.6	0.4	12.7	8.3	6.2	0.2	0.2	4.6	9.5	5.1
(*RS*)-DEHP	AVG	2.2	4.6	405.0	4.7	55.3	2.4	4.6	405.6	2.9	61.3
	SD	0.5	0.2	11.6	4.8	6.4	0.4	0.1	8.4	4.1	6.0
(SS)-DEHP	AVG	2.6	4.7	409.3	6.7	54.8	2.5	4.6	405.8	4.0	58.6
	SD	0.4	0.3	12.0	7.3	7.3	0.4	0.2	10.9	5.6	6.3
MEHP	AVG	1.7	4.3	295.6	31.9	114.0	1.3	4.3	296.9	16.8	111.9
	SD	0.5	0.2	3.9	36.7	3.7	0.3	0.1	3.6	8.0	4.8
DES	AVG	0.5	3.8	276.4	4.7	108.5	0.5	3.8	276.5	5.6	108.5
	SD	0.1	0.0	1.9	3.8	1.0	0.2	0.0	1.9	4.4	1.0
BPA	AVG	0.4	3.4	231.8	4.1	108.6	0.3	3.4	231.5	5.8	108.5
	SD	0.3	0.0	1.2	4.0	1.0	0.2	0.0	1.2	4.8	1.0
DDE	AVG	0.2	3.7	257.9	4.1	0.0	0.5	3.7	257.8	5.3	0.0
	SD	0.1	0.0	1.4	3.8	0.0	0.3	0.0	1.5	4.6	0.0
(RS)-PROP	AVG	1.0	4.0	291.4	5.5	50.8	1.1	3.5	230.1	5.1	65.9
	SD	0.2	0.1	4.0	6.1	5.1	0.2	0.1	2.6	4.9	2.9
(RR)-PROP	AVG	1.5	3.9	292.5	7.3	48.2	0.9	3.9	290.3	3.4	47.3
	SD	0.2	0.1	3.2	15.3	5.2	0.2	0.1	4.2	3.7	4.2
3OH-CF	AVG	0.5	3.6	236.3	4.2	105.6	1.1	3.6	235.8	6.7	104.0
	SD	0.2	0.1	1.7	4.5	3.1	0.2	0.1	2.3	6.2	3.5
CF	AVG	0.5	3.6	230.8	3.2	65.9	1.6	4.0	292.9	5.3	49.1
	SD	0.3	0.1	1.9	3.7	3.0	0.1	0.1	2.8	4.5	4.9
BPC/RU-486$_{AGO}$	AVG	0.4	3.6	243.6	4.4	108.5	0.5	4.6	408.9	9.9	81.5
	SD	0.1	0.0	1.4	4.0	1.0	0.1	0.0	2.3	6.3	1.5
BPC/RU-486$_{ANT}$	AVG	0.9	3.6	243.6	11.5	108.5	0.5	4.6	409.5	18.8	81.6
	SD	0.2	0.0	1.4	9.4	1.0	0.1	0.0	2.2	11.3	1.5
ENDO$_{AGO}$	AVG	0.8	5.0	395.5	32.2	53.5					
ENDO$_{ANT}$	SD	0.2	0.1	2.6	18.6	2.4					

Table S10. Fractional contribution of the top 5 variables for each principal component.
H = helix, L = loop, βa = beta-sheet, h = hydrophobic interactions, p=polar

**ERα**			
**PC1 (23.50%)**		**PC2 (16.91%)**	
RMSF_H12	0.25	H7_F425h	0.28
H5_R394p	0.24	L_RMSD	0.27
H4/5_L387p	0.24	L_MolSA	0.26
H4/5_L384h	0.23	L_rGyr	0.24
L_SASA	0.23	Ba_F404h	0.23
**GR**			
**PC1 (28.95%)**		**PC2 (16.97%)**	
H12H3	0.240	dG Hydro	0.28
RMSF_L:H11H12	0.24	H11_L732h	0.22
RMSF_H12	0.24	H11_F749h	0.22
RMSF_H11	0.22	Ba_F623h	0.19
H10H3	0.22	H11_C736h	0.18

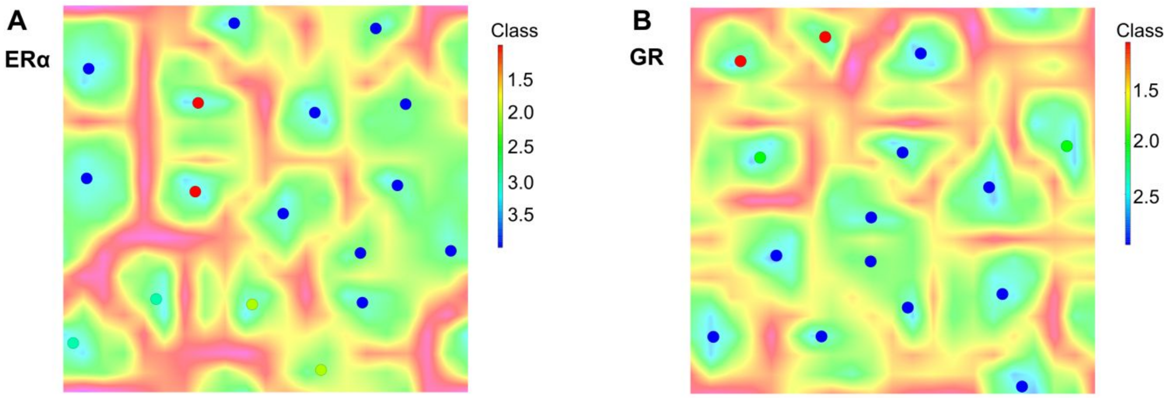

Figure S29. Unsupervised neural network Self-Organizing Maps (SOM) using default parameters (10 neurons per axis and the Gaussian Neighborhood function) were carried out using DataWarrior 5.2.1 software and the same set of variables from PCA, for ERα (A) and GR (B).

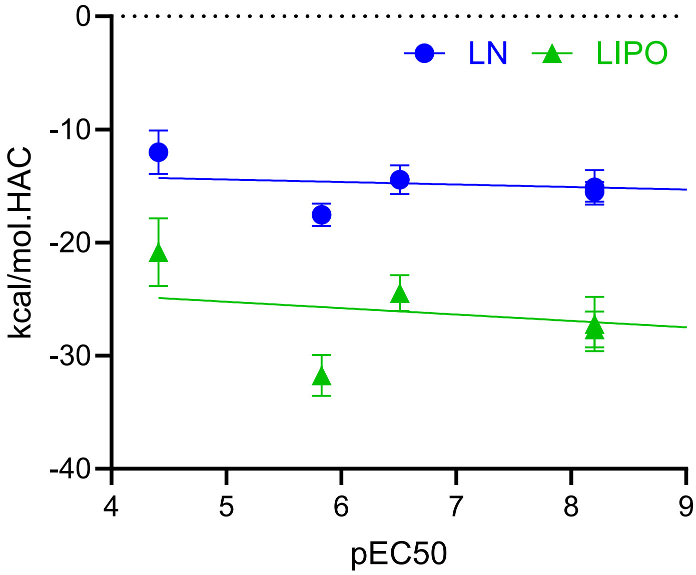

Figure S30. Correlation between predicted binding energy (LN) and lipophilic component (Lipo) and EC50 values (expressed as pEC50) determined by reporter gene assays for ERα

	ERα	ERβ	GR	AR	MR	PR
H3	T347	T299	N564	N705	N770	N719
H3	E353	E305	Q570	Q711	Q776	Q725
H5	R394	R346	R611	R752	R817	R766
H7	M421	I373	Q642	M780	M845	F794
H11	H524	H475	T739	T877	T945	T894

Figure S31. Amino acids with relevant interactions on SHRs-EDCs are shown as sticks in the upper panels and listed in the lower table (E).
